# Evaluating the risk of digestive system cancer in autoimmune disease patients: a systematic review and meta-analysis focusing on bias assessment

**DOI:** 10.1016/j.eclinm.2025.103410

**Published:** 2025-08-07

**Authors:** Julia Reizner, Simone Fischer, Jakob Linseisen, Christa Meisinger, Dennis Freuer

**Affiliations:** Epidemiology, Faculty of Medicine, University of Augsburg, University Hospital of Augsburg, Stenglinstr. 2, 86156, Augsburg, Germany

**Keywords:** Digestive system cancer, Autoimmune diseases, Type 1 diabetes, Lupus erythematosus, Celiac disease

## Abstract

**Background:**

There is emerging evidence that certain autoimmune diseases can modulate the risk for digestive system cancer. However, limitations of non-experimental studies may lead to diverging results. Thus, the aim was to evaluate the available evidence and provide bias-minimized estimates for the associations between celiac disease (CD), systemic lupus erythematosus (SLE), multiple sclerosis (MS), and type 1 diabetes (T1D) and different digestive system cancers.

**Methods:**

Systematic review (PROSPERO: CRD42024553216) was conducted according to PRISMA guidelines. Scientific publications were searched in PubMed, Web of Science, Embase, and Cochrane Library from inception up to May 2, 2025, with no restrictions on publication date. ROBINS-E tool was used for examining the study-specific risk of bias. Inverse-variance weighted random-effects models were performed as the primary meta-analytic approach. Heterogeneity was quantified and adjusted for in a comprehensive bias assessment including several analyses.

**Findings:**

This study included 237 estimates from 47 studies covering over 1.5 million cases of any ethnicity. CD, SLE, and T1D were positively associated with pancreatic, esophageal, colon, liver, and hepatobiliary cancers. Additionally, T1D was positively associated with stomach and colorectal cancers. The strongest bias-corrected association was found between CD and small intestine cancer (RR = 4.19; 95% CI: [2.71; 6.50]). MS was inversely associated with pancreatic, esophageal, rectal, and colorectal cancers.

**Interpretation:**

This study provides new insights into the evidence for digestive system cancer risk related to autoimmune diseases by adjusting for multiple sources of bias. As a next step, potential mechanisms responsible for the different associations should be investigated.

**Funding:**

The study received academic funding from the Faculty of Medicine, University of Augsburg, Germany.

**Research in context**

**Evidence before this study**

The results of previous studies on digestive system cancer risk in patients with autoimmune diseases that occur frequently early in life, that is celiac disease, systemic lupus erythematosus, multiple sclerosis, and type 1 diabetes, have been inconsistent. A literature search was conducted without restriction on publication date up to May 2, 2025, in the PubMed, Embase (via Ovid), Cochrane Library, and Web of Science databases to identify English or German written peer-reviewed publications of observational studies (without cross-sectional studies) presenting risk estimates on any scale. Finally, 237 estimates from 47 studies, of which 2, 5, and 40 had low, moderate, and high risk of bias, respectively, could be used in subsequent meta-analyses.

**Added value of this study**

In this systematic review and meta-analysis, the association between autoimmune diseases and the risk of developing digestive system cancers was evaluated by considering various sources of bias. Bias corrected estimates were calculated for the risk of digestive system cancer in celiac disease, systemic lupus erythematosus, multiple sclerosis, and type 1 diabetes patients. New insights into the link between the mentioned autoimmune diseases and the risk of various types of digestive system cancers were provided.

**Implications of all the available evidence**

The risk of developing cancer of the stomach, esophagus, small intestine, colorectum, hepatobiliary tract, and pancreas in individuals with celiac disease, systemic lupus erythematosus, multiple sclerosis, and type 1 diabetes should be further investigated. If further evidence will confirm these findings, clinical surveillance strategies and personalized cancer screening should be implemented for patients with these autoimmune diseases.

## Introduction

Worldwide, digestive system cancers account for 26% of cancer incidence and 35% of all cancer-related deaths, but there are geographical differences.^1^^,^^2^ The most common digestive system cancers cancer sites of the GI tract are malignancies of the colorectum, stomach, liver, esophagus, and pancreas, respectively.^2^ A significant increase in the crude numbers of new cases and deaths is expected for each of the five cancers in almost all regions of the world.^2^ As there are usually no specific symptoms for early digestive system cancers, many tumors are only diagnosed at an advanced stage, when treatment options are often limited.^2^ Therefore, paying special attention to vulnerable groups can enable targeted screening and thus contribute to the early detection of these cancers.^2^ Prior studies reported that more than half of all digestive system cancers are due to modifiable risk factors, such as alcohol consumption, smoking, unbalanced diet, and obesity.^3^ There is increasing evidence that autoimmune diseases are also associated with an elevated risk.^4^ In autoimmune diseases, the body's own structures are attacked by components of the immune system leading to systemic inflammation in the affected tissue and permanent immune reactions, necessitating lifelong treatment.^5^ Long-term stress on organs and tissue due to the autoimmune reactions and drug treatment may explain a connection between autoimmune disease and the development of tumors.^6^ In particular, for adolescents and young adults with autoimmune diseases an increased risk of cancer deserves particular attention,^7^ because they often have a more severe disease phenotype and live for many years with the disorder and the associated immunomodulatory treatments.^7^^,^^8^ While for some autoimmune diseases (e.g. inflammatory bowel disease^9^^,^^10^), which occur at a young age, the risk of digestive system cancer is well understood, previous results have been inconsistent for other autoimmune diseases, e.g. due to insufficient statistical power, patient selection, disease definitions and follow-up duration. In addition, for several autoimmune diseases (e.g. type 1 diabetes (T1D)), it is still unclear whether there is an association with cancers of the digestive system.^11^ The inconclusive results of previous studies may also be due to bias caused by systematic errors or limitations in design or analysis, which are likely to lead to erroneous conclusions. Nevertheless, gaining knowledge is essential to develop clinical surveillance strategies for these patients and to plan and implement personalized cancer prevention. Therefore, this systematic review and meta-analysis aimed to summarize the evidence from observational studies on whether the presence of some selected autoimmune diseases with a manifestation frequently in young age is associated with the development of the most common digestive system cancers. More precisely: Based on the PICO framework, we aimed to determine the risk of developing cancer of the stomach, esophagus, small intestine, colorectum, hepatobiliary tract, and pancreas (outcomes) in individuals with celiac disease (CD), type 1 diabetes mellitus (T1D), multiple sclerosis (MS), and systemic lupus erythematosus (SLE) (exposures) compared to individuals without these conditions (comparator) from the general population. Moreover, reliability of estimates and discrepancies between study-specific results were analyzed in a comprehensive bias assessment and bias-adjusted estimates were presented, incorporating all the evidence from these analyses.

## Methods

### Scope of the review

Scoping the literature identified a sufficient number of studies assessing specific digestive system cancers, namely cancers of the pancreas, stomach, esophagus, small intestine, colon, rectum, liver, and gallbladder. As some studies reported associations only for colorectal cancer without subgroup differentiation (possibly due to low number of cancer-cases), further analyses were conducted, combining estimates for colon and rectal cancers, which were previously analyzed individually, with colorectal cancer. The same applied to liver and gallbladder cancers, which were combined with hepatobiliary cancer in additional analyses. Therefore, the analysis strategy in this work comprised the relationships of CD, SLE, MS, and T1D with the following cancers:

(1) Stomach
(2) Esophagus
(3) Pancreas
(4) Small intestine
(5) Colorectum
  a. Colon
  b. Rectum
(6) Hepatobiliary
  a. Liver
  b. Gallbladder

The study protocol was registered at PROSPERO (international prospective register of systematic reviews; Registration ID: CRD42024553216) and the systematic review was performed according to the PRISMA statement guidelines.^12^

### Search strategy and selection criteria

The literature search was conducted in the PubMed, Embase (via Ovid), Cochrane Library, and Web of Science databases and considered publications from inception up to May 2, 2025, with no restrictions on publication date. A search string was developed and adapted in advance for each database [Supplementary Table S1]. The results were specified by using controlled vocabulary, Boolean operators, free-text terms and Medical Subject Headings (MeSH terms). According to the method of Bramer et al. the references were entered and deduplicated in the EndNote literature management software.^13^ Finally, handsearching (i.e. backward and forward citation searching) was conducted in relevant publications (original articles and reviews) to identify further studies.

Based on the PICO-framework the inclusion criteria were defined as follows. Population: Men and women of any ethnicity. Exposures: Diagnosis of an autoimmune disease (CD, T1D, MS, SLE). Comparator: Individuals without any autoimmune disease from the respective population. Outcomes: Diagnosis of digestive system cancer (pancreas, stomach, esophagus, intestine, colorectum, or hepatobiliary system).

The eligibility criteria for studies comprised the following requirements: (1) Prospective or retrospective quantitative study design (e.g. cohort and case–control studies). (2) Peer reviewed articles (i.e. no case reports, conference abstracts or other non-peer reviewed articles). (3) Full text available in English or German language. (4) Diagnoses of exposure before the diagnosis of outcome (i.e. only incident cancer cases, no cross-sectional studies). (5) Point estimates and confidence intervals on any scale. Existing reviews and meta-analyses were excluded to avoid double inclusion of original studies.

Titles and abstracts were each screened independently by two of three investigators (J.R, D.F, and C.M.). Potentially relevant studies were subjected to full-text screening with regard to the inclusion criteria. Disagreements about inclusion of studies were discussed between the responsible reviewers.

### Data extraction and evaluation of studies

Estimates and study-characteristics were extracted and validated independently by two of three investigators (J.R, D.F, and C.M.). As far as possible, the following information was obtained using a standardized form: title, authors, year of publication, study design, exposure and outcome assessed, country where the study was conducted, definition of the control group, number of cases with the respective autoimmune disease, number of controls, number of incident cancer-cases, study duration, type and strength (i.e. point estimate and confidence interval) of an effect estimate, adjustment variables, time window between diagnosis of exposure and outcome, and study-specific characteristics that were helpful in evaluating the reliability of a reported estimate. Sex-specific results were included in the analyses, if no overall estimate was reported. When multiple estimates were given, the more precise one (e.g. adjusted HR preferred over SIR) or the more reliable one (e.g. a sufficient time window) was used.

Estimates with a lower confidence interval (CI) limit of 0 on the RR-scale undergo a multi-stage procedure. Initially, the lower limit was estimated based on the point estimate and the upper CI limit. If there was a significant difference between 0 and the estimate (most likely due to rounding reasons of the reported point estimate and upper CI limit), the corresponding authors were contacted. If no answer was given or the exact result could not be reconstructed by the corresponding author, the respective estimate was excluded from the meta-analyses, as it would lead to infinitesimal variances.

The reliability of each included study was assessed using the ROBINS-E tool (Risk Of Bias in Non-randomized Studies of Exposures) by evaluating the study-specific bias based on the predefined default definitions due to the following domains: Unmeasured confounding, measurement accuracy of exposure and outcome, selection of participants, post-exposure interventions, presence and handling of missing data, and selection of reported results.^14^ Discrepancies were resolved by discussion between the two responsible investigators.

### Data synthesis and statistics

Due to differences in the study designs, the considered studies provided their results in form of hazard ratios (HR), odds ratios (OR), standardized incidence ratios (SIR), incidence rate ratios (IRR), and relative risks (RR). Since each of the cancer entities under investigation is among the low-prevalence diseases (i.e. prevalence below 10%) in the general population, all of these ratios approximate the underlying RR. The conversion to RR using the formulas $RR=\frac{OR}{\left(1-p\right)+\left(p\cdot OR\right)}$ for ORs and $RR=\frac{1-e^{HR.\ln\left(1-p\right)}}{p}$ for HRs confirmed this assumption.^15^^,^^16^ Thus, all estimates in this study were presented on the RR-scale.

The inverse-variance weighted (IVW) random-effects model was the primary method used in meta-analyses to pool the effect estimates from different studies. Higgins' I^2^, $\tau^{2}$, and Cochran's Q statistics were calculated to quantify and assess the between-study heterogeneity. The $\tau^{2}$ was calculated by the Paule-Mandel estimator, as this has been shown to be the recommended method for estimating heterogeneity variance in meta-analyses of binary outcomes.^17^

### Sensitivity analyses

Sources of heterogeneity were analyzed in a comprehensive bias assessment based on a series of sensitivity analyses, starting with an outlier analysis to adjust for studies that differed significantly from the others. The influence analysis aimed to identify high leverage studies mainly responsible for the variability in effect estimates and included the Baujat Diagnostics as well as leave-one-out analyses.

For associations based on at least 10 included studies, further heterogeneity structures were examined. In this context the multi-model inference and meta-regression were performed to quantify the importance of study-specific characteristics that explained the variability of effect estimates. Subgroup effects and differences were analyzed using several mixed-effects models to investigate specific patterns of heterogeneity. This modelling strategy follows the fixed-effects plural model outlined in Borenstein and Higgins, where the pooled effect size for each subgroup is calculated using a random-effects model and the test for subgroup differences is performed using a fixed-effect model.^18^ Small-study effects (as proxy for publication bias) were assessed using Egger's and Begg's tests for funnel plot asymmetry and, if necessary, bias was corrected applying the trim and fill method.

For each investigated association, the evidence from all approaches mentioned above was used to evaluate the plausibility of the pooled estimates and thus the presence of an effect. Both unadjusted and bias-corrected estimates were presented.

The Meta-analyses followed the MOOSE (Meta-analyses Of Observational Studies in Epidemiology) reporting guidelines. Statistical tests were performed two-sided based on a type I error α = 0.05. In this context, $P_{Q}$ denotes the P-value for the test of the Cochran's Q statistic as a measure of heterogeneity. Point estimates and corresponding 95% CIs were presented on the multiplicative RR-scale, where RR > 1 meaning an increased risk for a cancer-outcome in the presence of a specific exposure compared to the general population. Analogously, an RR between 0 and 1 represents a decreased risk. To account for multiple testing, the false discovery rate (FDR) correction was applied to P-values in the primary analyses (denoted as $P_{FDR}$). All analyses were performed using the open-source statistical software R (version 4.4.2). The packages dplyr (version 1.1.4), meta (8.0–1), metafor (4.6–0), dmetar (0.1.0), weightr (2.0.2), RoBMA (3.2.0), writexl (1.5.1), and ggplot2 (3.5.1) were mainly used for data handling, meta-analyses, and the creation of tables and illustrations, respectively. During the systematic review process, the references were managed using Endnote 21 literature management software. The online tool robviz (Risk-Of-Bias VISualization) was used to visualize the study-specific risk of bias.^19^

### Ethics

As this systematic review and meta-analysis based on data extracted from previously published studies, ethical approval and informed consent were not required.

### Role of funding source

The study received academic funding from the Faculty of Medicine, University of Augsburg, Augsburg, Germany. The funder had no role or influence in the design, data collection, data analysis, and reporting of this study. There was no additional funding from any agency in the public, commercial, or not-for-profit sectors.

## Results

**Fig. 1:**
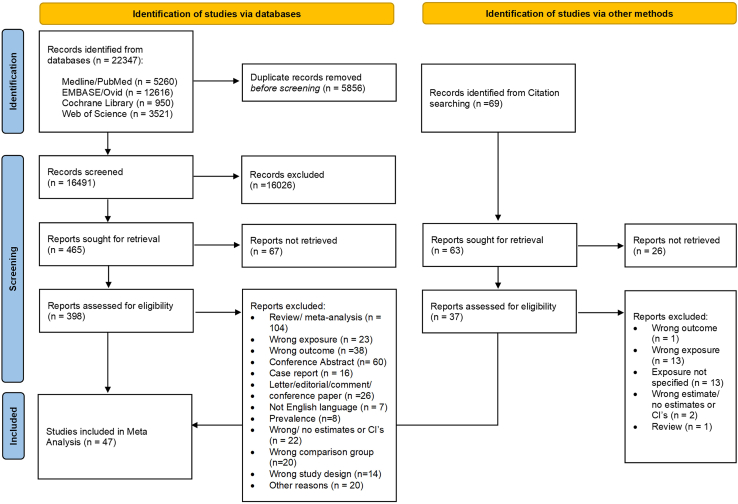
PRISMA flowchart showing the study selection process for the associations between selected autoimmune diseases and digestive system cancers.

After removing duplicates, the conducted search in the databases PubMed, Embase, Cochrane Library, and Web of Science yielded 16,491 records [Fig. 1]. Sixty-nine further studies were identified through the forward and backward citation search. The screening process, consisting of title/abstract and full-text screening, identified 47 studies meeting the inclusion criteria and therefore being reliable for the subsequent meta-analyses [Supplementary Table S2, Supplementary References S1].

The most common reasons for exclusion in the full-text screening were that publications were reviews (n = 104), conference abstracts (n = 60), or case reports (n = 16) or that they investigated the wrong exposures or outcomes (n = 61) [Fig. 1, Supplementary Table S3].

In total, the included studies comprised over 1,518,106 autoimmune cases of interest (Landgren et al. did not report specific frequencies) [Supplementary Table S3]. However, the number of controls was not provided in most of the studies assessed.

Based on the ROBINS-E algorithm, 40 of the 47 studies had a serious, 5 had moderate, and 2 had low overall risk of bias [Supplementary Figure S1]. Common deficiencies were insufficiently controlled confounding (high risk in the first domain) and some concerns in the third domain due to the selection of participants (uncertainties in the definition of T1D) [Supplementary Figure S2]. As most associations were based on observational evidence with high risk of bias, our confidence in the precise magnitude of risk was low to moderate.

The meta-analyses revealed 14 models with and 22 models without notable heterogeneity [Supplementary Figures S3–S6]. For two associations only one study was found that met the inclusion criteria. The main factors influencing differences in study-estimates were the number autoimmune diseases and cancer cases, study duration, and the time window between the diagnosis of an exposure and an outcome [Supplementary Figure S7]. In the following, results from main and sensitivity analyses are reported, grouped according to the individual exposures.

### Associations with celiac disease

**Fig. 2:**
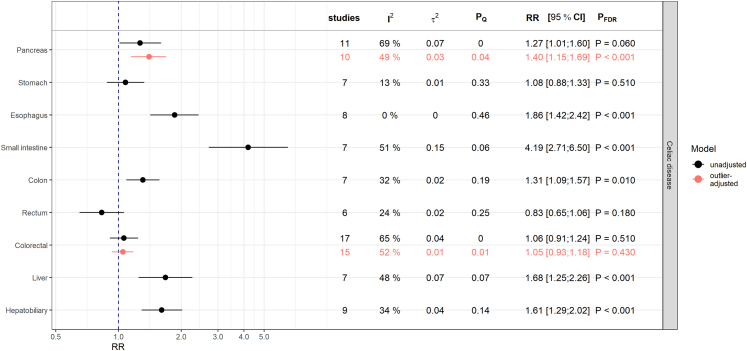
Results from meta-analyses on the relative risk (RR) scale for the associations between celiac disease and digestive system cancers. The estimates (RR and corresponding 95% confidence intervals) derived from inverse-variance weighted random-effects models are shown in black and red for associations before and after adjustment for outliers, respectively. Presented P-values (P_FDR_) are adjusted for multiple testing using the false discovery rate (FDR) correction. P_Q_ denotes the P-value testing the Cochran's Q statistic as a measure of between-study heterogeneity. I^2^ represents another measure of heterogeneity and $\tau^{2}$ is an estimate for the variance of true effect sizes.

CD was positively associated with esophageal cancer (RR = 1.86; 95% CI: [1.42; 2.42]; $P_{FDR}\;<$ 0.001), small intestinal cancer (RR = 4.19; 95% CI: [2.71; 6.50]; $P_{FDR}\;<$ 0.001), colon cancer (RR = 1.31; 95% CI: [1.09; 1.57]; $P_{FDR}$ = 0.010), liver cancer (RR = 1.68; 95% CI: [1.25; 2.26]; $P_{FDR}\;<$ 0.001), and hepatobiliary cancer (RR = 1.61; 95% CI: [1.29; 2.02]; $P_{FDR}\;<$ 0.001) [Fig. 2].

After excluding the study by Ilus et al.,^20^ which was identified as an outlier and the most influential study, the pooled estimate for the association with pancreatic cancer increased to RR = 1.40 (95% CI: [1.15; 1.69]; $P_{FDR}\;<$ 0.001), indicating a positive relationship [Supplementary Figures S8 and S9]. The heterogeneity in this model decreased to I^2^ = 49% ($\tau^{2}$ = 0.03, $P_{Q}$ = 0.04) [Fig. 2]. Meta-regression and subsequent subgroup analyses revealed differences in the type of effect estimates, with studies reporting HRs unanimously (I^2^ = 0%, $P_{Q}$ = 0.89) suggesting positive associations (HR = 1.57; 95% CI: [1.35; 1.83]; P< 0.0001) while the other estimate types did not [Supplementary Table S4, Supplementary Figure S10].

Looking at the association with colorectal cancer, the meta-regression revealed that heterogeneity could be completely attributed to the different estimates for colon and rectum cancers as well as the time window between diagnosis of CD and the outcome [Supplementary Table S4]. In a subgroup analysis, studies with a sufficient time window (larger than one year) found no association between CD and colorectal cancer [Supplementary Figure S10].

### Associations with systemic lupus erythematosus

**Fig. 3:**
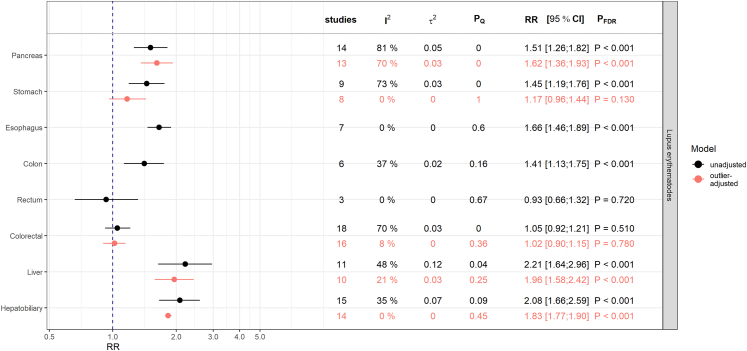
Results from meta-analyses on the relative risk (RR) scale for the associations between systemic lupus erythematosus and digestive system cancers. The estimates (RR and corresponding 95% confidence intervals) derived from inverse-variance weighted random-effects models are shown in black and red for associations before and after adjustment for outliers, respectively. Presented P-values (P_FDR_) are adjusted for multiple testing using the false discovery rate (FDR) correction. P_Q_ denotes the P-value testing the Cochran's Q statistic as a measure of between-study heterogeneity. I^2^ represents another measure of heterogeneity and $\tau^{2}$ is an estimate for the variance of true effect sizes.

SLE was positively associated with pancreatic cancer (RR = 1.51; 95% CI: [1.26; 1.82]; $P_{FDR}\;<$ 0.001), esophagus cancer (RR = 1.66; 95% CI: [1.46; 1.89]; $P_{FDR}\;<$ 0.001), colon cancer (RR = 1.41; 95% CI: [1.13; 1.75]; $P_{FDR}\;<$ 0.001), liver cancer (RR = 2.21; 95% CI: [1.64; 2.96]; $P_{FDR}\;<$ 0.001), and hepatobiliary cancer (RR = 2.08; 95% CI: [1.66; 2.59]; $P_{FDR}\;<$ 0.001) [Fig. 3].

Although SLE seemed to be associated with stomach cancer, the evidence for a relationship disappeared (RR = 1.17; 95% CI: [0.96; 1.44]; $P_{FDR}$ = 0.130) after removing the study of Chen et al.^21^ identified as an extreme outlier [Supplementary Figure S11]. The estimate of this outlier (RR = 2.08; 95% CI: [1.97; 2.19]) differed notably from other study-specific results with point estimates in a range between 1.09 and 1.53 (I^2^ = 0%) [Supplementary Figure S12].

For the association with pancreatic cancer the overall estimates before and after outlier-removal were consistent. However, there was still a considerable degree of heterogeneity (I^2^ = 70.4%, $\tau^{2}$ = 0.03, $P_{Q}\;<$ 0.001), which can be explained away by adjusting for the study-specific region, with positive associations in Asian (RR = 2.00, 95% CI: [1.81; 2.20]; P< 0.001) and European studies (RR = 1.44, 95% CI: [1.34; 1.56]; P< 0.001) but not in the studies from North America (RR = 1.24, 95% CI: [0.87; 1.78]; P = 0.230) [Supplementary Table S4, Supplementary Figure S13].

### Associations with multiple sclerosis

**Fig. 4:**
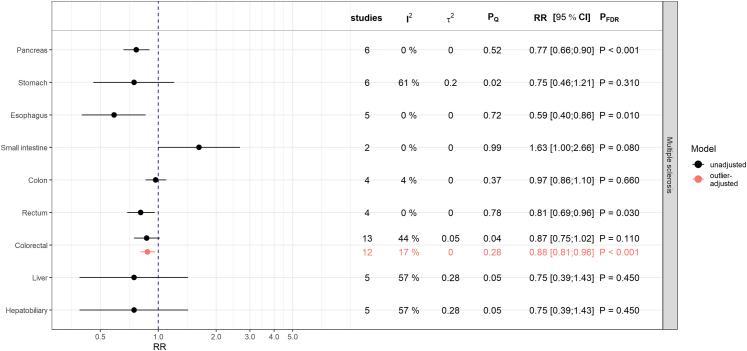
Results from meta-analyses on the relative risk (RR) scale for the associations between multiple sclerosis and digestive system cancers. The estimates (RR and corresponding 95% confidence intervals) derived from inverse-variance weighted random-effects models are shown in black and red for associations before and after adjustment for outliers, respectively. Presented P-values (P_FDR_) are adjusted for multiple testing using the false discovery rate (FDR) correction. P_Q_ denotes the P-value testing the Cochran's Q statistic as a measure of between-study heterogeneity. I^2^ represents another measure of heterogeneity and $\tau^{2}$ is an estimate for the variance of true effect sizes.

MS was inversely associated with pancreatic cancer (RR = 0.77; 95% CI: [0.66; 0.90]; $P_{FDR}\;<$ 0.001), esophageal cancer (RR = 0.59; 95% CI: [0.40; 0.86]; $P_{FDR}$ = 0.010), and rectal cancer (RR = 0.81; 95% CI: [0.69; 0.96]; $P_{FDR}$ = 0.030) [Fig. 4].

Additionally, there was an inverse association between MS and colorectal cancer (consisting of four colon, rectal, and colorectal cancer studies, respectively) (RR = 0.88; 95% CI: [0.81; 0.96]; $P_{FDR}\;<$ 0.001, I^2^ = 16.7%) after omitting the outlier-study of Buttmann et al.,^22^ which differed notably in direction and magnitude from the other estimates [Supplementary Figures S14 and S15].

### Associations with type 1 diabetes

**Fig. 5:**
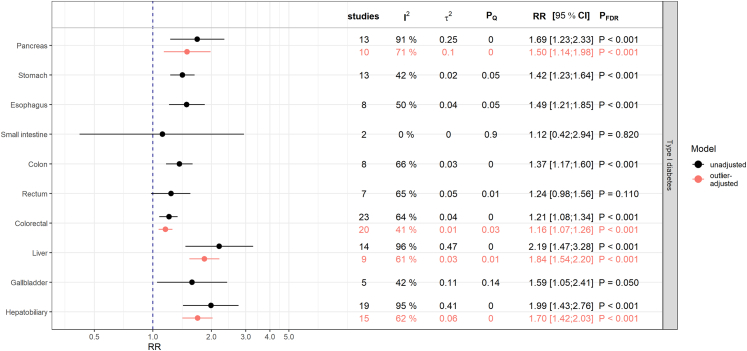
Results from meta-analyses on the relative risk (RR) scale for the associations between type 1 diabetes mellitus and digestive system cancers. The estimates (RR and corresponding 95% confidence intervals) derived from inverse-variance weighted random-effects models are shown in black and red for associations before and after adjustment for outliers, respectively. Presented P-values (P_FDR_) are adjusted for multiple testing using the false discovery rate (FDR) correction. P_Q_ denotes the P-value testing the Cochran's Q statistic as a measure of between-study heterogeneity. I^2^ represents another measure of heterogeneity and $\tau^{2}$ is an estimate for the variance of true effect sizes.

T1D was strongly associated with all cancers considered, with the exception of cancers of the small intestine and rectum [Fig. 5]. However, some models exhibited substantial heterogeneity, which could be considerably reduced after omitting outliers with a high leverage [Supplementary Figures S16 and S17]. The only exception was the model for the association with pancreatic cancer. Neither the outlier-adjustment nor the influence analysis could explain a notable proportion of the heterogeneity. However, the meta-regression revealed that the study-region and the type of estimate were responsible for the heterogeneity [Supplementary Table S4]. Subgroup analyses showed strong positive associations ($I^{2}$ = 18.1%, $P_{Q}$ = 0.27) in Asian studies (RR = 3.37; 95% CI: [2.17; 5.22]; P< 0.001) and consistently positive but heterogeneous associations for the individual estimate types [Supplementary Figure S18].

### Publication bias

While the Begg's test was not able to identify any funnel plot asymmetry, the Egger's test indicated small-study bias in three models (SLE with colorectal cancer as well as T1D with both pancreatic and hepatobiliary cancers) [Supplementary Table S5, Supplementary Figure S19]. For all of these associations, the small-study bias was only detected in the initial models ($P_{Egger}$ between 0.025 and 0.042), but not in the models after outlier-adjustment, indicating the outliers were responsible for the funnel plot asymmetries. There was no further evidence of publication bias for any of the remaining associations [Supplementary Table S5].

### Sensitivity analyses

To summarize, after excluding outlier or high-leverage studies, which differed notably from the other studies and thus accounted for a considerable amount of heterogeneity, all key associations remained directionally consistent. Similarly, trim-and-fill adjustment did not substantially change the estimates from main analyses. In models without outliers no small-study bias could be detected. The subgroup analyses revealed region-specific differences for some associations with consistent results regarding the direction of estimates. The same states for the different types of estimates in the studies assessed. Studies with a time window between exposure and outcome diagnosis of less than one year often yielded stronger but consistent estimates.

**Fig. 6:**
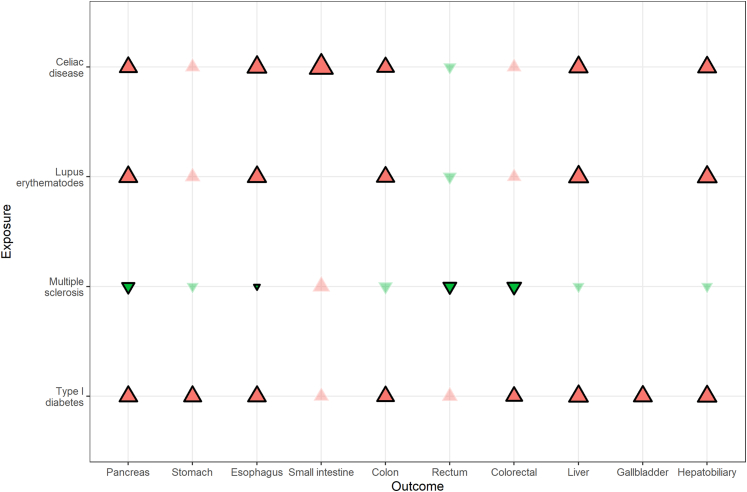
Summary of bias-corrected results after adjusting for all identified biases. Exposures are shown on the vertical axis, the outcomes on the horizontal axis. Red and green triangles represent positive and negative effect estimates, respectively. The size of the triangles represents the log (RR). Found associations were highlighted by black outlined and stronger colored triangles.

From all these approaches, it can be summarized that CD, SLE, and T1D were positively associated with pancreatic, esophageal, colon, liver, and hepatobiliary cancers [Fig. 6, Supplementary Table S6]. In addition, T1D was associated with stomach and colorectal cancers, while CD was associated with small intestine cancer. MS was the only autoimmune disease showing inverse associations with cancers of the pancreas, esophagus, rectum, and colorectum.

## Discussion

In this study, a comprehensive systematic review and meta-analysis of selected autoimmune diseases (CD, SLE, MS, and T1D) and the risk of common digestive system cancers (pancreas, stomach, esophagus, small intestine, colon, rectum, liver, and gallbladder) were conducted. Meta-analyses with a comprehensive assessment of bias corroborated known or found several new associations between the autoimmune diseases considered and various cancers of the digestive system. In doing so, this work summarized the current stage of research in this field.

In the present study, CD was positively associated with pancreatic, liver, hepatobiliary tract, esophageal, colon, and small intestine cancers. There was no association with stomach, rectal, or colorectal cancers. The lack of association between CD and stomach cancer has also been confirmed by other studies.^4^^,^^23^ In a recent meta-analysis by Gromny et al.,^24^ which included 5 studies, CD was positively associated with pancreatic cancer (HR = 1.46; 95% CI: [1.26; 1.70]). This result agrees very well with our pooled estimate across 10 studies (RR = 1.40; 95% CI: [1.15; 1.69]). Furthermore, our results confirm the findings of a meta-analysis by Han et al. who found positive associations with esophageal and small intestine cancers and no associations with rectal and stomach cancers.^25^ However, the pooled estimates in the study by Han et al. for esophageal cancer (HR = 3.72; 95% CI: [1.90; 7.28]) and small intestine cancer (HR = 14.41; 95% CI: [5.53; 37.60]) were considerably higher than our results (esophageal cancer: RR = 1.86; 95% CI: [1.42; 2.42] and small intestine cancer: RR = 4.19; 95% CI: [2.71; 6.50]). The positive associations with liver (RR = 1.68; 95% CI: [1.25; 2.26]) and colon cancers (RR = 1.31; 95% CI: [1.09; 1.57]) in CD patients identified in our study has not yet been reported in prior meta-analyses. Despite consistent point estimates, Han et al. found no evidence for an increased risk of colon, liver, and pancreatic cancer in CD patients. This may be due to the large heterogeneity in their models because of including studies that did not meet our quality criteria. The $I^{2}$ for these associations in their study ranged from 58% to 76%, compared with $I^{2}$ values of 32%–49% in our analyses.^25^

Our findings of positive associations between SLE and cancers of the pancreas, esophagus, colon, liver, and hepatobiliary tract and no associations with rectal and colorectal cancers are consistent with the results of the study by Zhang et al.^26^ The pooled estimates for pancreatic, esophageal, and colorectal cancers in our study were very similar to those from the study by Zhang et al., however, the estimates for liver (HR = 2.93; 95% CI: [1.55; 5.49]) and hepatobiliary cancer (HR = 2.45; 95% CI: [1.54; 3.88]) were higher in Zhang's study than in ours (liver cancer: RR = 1.96; 95% CI: [1.58; 2.42] and hepatobiliary cancer: RR = 1.83; 95% CI: [1.77; 1.90]) after adjustment for outliers. The same applies to the study by Cao et al., who reported significantly positive associations with esophageal (HR = 1.86; 95% CI: [1.21; 2.88]) and liver cancer (HR = 3.21; 95% CI: [1.70; 6.05]) in patients with SLE.^27^ In accordance with Zhang et al.^26^ but contrary to some previous systematic reviews and meta-analyses^4^^,^^28^^,^^29^ we did not find an association with stomach cancer in SLE patients (RR = 1.17; 95% CI: [0.96; 1.44]). The contradictory finding of these previous studies with point estimates ranging from 1.31 to 1.37 is likely to be due to the outlier-adjustment performed in our study. The present result regarding a positive association between SLE and pancreatic cancer (RR = 1.62; 95% CI: [1.36; 1.93]) is not in agreement with the findings by Clarke et al. who reported no association (HR = 1.26; 95% CI: [0.97; 1.63]).^29^ The discrepancy may be since we were able to include 13 studies in our meta-analysis, in contrast to Clarke, who included 7 studies. This fact in addition enabled us to conduct region-specific analyses. While we found positive associations in Asian and European studies, there was no association in North American studies. The regional differences in cancer susceptibility could be due to environmental factors, lifestyle, and differences in genetic predisposition.^30^ Inconsistencies in risk assessment can also be caused by differences in SLE treatment guidelines in terms of general indication, drug type and dosage in the respective countries.^31^

In the present study, MS was the only autoimmune disease showing inverse associations with pancreatic, esophageal, rectal, and colorectal cancers, while there was no relationship with the other digestive system cancers investigated considering the bias-corrected estimates. However, the observed associations were somewhat weaker (due to the point estimates, confidence intervals, and P-values) compared to the other investigated autoimmune diseases in this study. The findings for an inverse association with pancreatic cancer (RR = 0.77; 95% CI: [0.66; 0.90]) and no significant association with stomach cancer (RR = 0.75; 95% CI: [0.46; 1.21]) were in line with the results of two previous meta-analyses, which roughly reported comparable point estimated.^23^^,^^32^ However, the result regarding stomach cancer (HR = 0.64; 95% CI: [0.45; 0.92]) provided by Song et al. based on two studies was contradictory to our finding, which was based on 6 studies.^4^ So far, there have been no prior systematic reviews and meta-analyses which reported inverse relationships with esophageal and rectal cancers in MS patients compared to the general population.^33^, ^34^, ^35^, ^36^ Whether the inverse association between MS and cancer of the digestive system, including rectal cancer, is a genuine phenomenon or is due to lifestyle factors or medication use needs to be the subject of further research.

In individuals with T1D, we found positive associations with all cancers examined except for small intestine and rectum. The positive associations with stomach^4^^,^^23^^,^^37^^,^^38^ and pancreatic cancers^38^^,^^39^ reported in prior systematic reviews and meta-analyses were supported; the point estimates from our analyses were also largely consistent with those found in previous studies. The present results of an positive association with esophageal cancer (RR = 1.49; 95% CI: [1.21; 1.85]) and liver cancer (RR = 1.84; 95% CI: [1.54; 2.20]) are consistent with a systematic review and meta-analysis by Sona et al.^38^ (26). However, the relative risks calculated by Sona et al. for both cancer types were slightly higher than our results. The present finding of an association between T1D and colorectal cancer (RR = 1.16; 95% CI: [1.07; 1.26]) was not in line with the results by Sona et al. (HR = 0.90; 95% CI: [0.61; 1.31]).^38^ This might be due to the small number of only two studies included in that former meta-analysis, whereas we were able to include 20 studies. For the first time, we found positive associations between T1D and gallbladder (RR = 1.59; 95% CI: [1.05; 2.41]), hepatobiliary (RR = 1.70; 95% CI: [1.42; 2.03]), and colon cancers (RR = 1.37; 95% CI: [1.17; 1.60]) compared to the general population.

Although autoimmune processes presumably play an important role in the development of various types of cancers, the underlying mechanisms remain largely unknown.^6^ Chronic inflammation, a hallmark of autoimmune diseases, can promote tumor development, progression, and invasion.^40^ In addition, similar patterns of immune dysregulation characterize these conditions. Common factors such as infections, poor dietary habits, and environmental factors can cause chronic cell damage and trigger either autoimmune diseases or cancer.^41^ Furthermore, cancer and autoimmunity seem to be bidirectionally related, with both conditions perpetuating each other through inflammatory processes.^42^ Type 2 inflammation involving cytokines like IL-4 and IL-13, may initiate epigenetic reprogramming of epithelial cells, leading to metaplastic differentiation and malignant transformation.^6^ Shared mechanisms in the pathogenesis of autoimmune diseases and cancer, such as serum autoantibodies and genomic susceptibility may also contribute to their association.^6^^,^^43^ Furthermore, autophagy, a cellular process involved in both immune responses and tumor progression, may play a crucial role in this regard, although its specific functional mechanism remains unclear.^44^ Finally, immunosuppressive drugs may play a role in determining neoplastic potential.^43^

The exact mechanisms explaining the inverse associations with some cancers in MS patients remain unclear, but it could be hypothesized that behavioral changes in MS patients may play a role.^34^ In addition, prior studies suggested that the immunologic characteristics of MS disease activity may improve antitumor surveillance.^34^ However, patients with MS are also subject to immunomodulatory therapies, potentially altering cancer risk.^22^^,^^45^ Earlier studies suggested an increased risk of cancer in patients treated with immunosuppressants for their MS.^36^^,^^45^ Disease-modifying agents introduced later that have been shown to alter the natural history of MS include interferon (IFN)-β1b, IFN-β1a, glatiramer acetate, mitoxantrone and natalizumab.^46^ IFN-β, for example, blocks the potent proinflammatory agent IFN-γ and other inflammatory cytokines that can cause immune-mediated damage to myelin cells.^47^ Subsequent studies examining the risk of malignancy in MS patients receiving disease-modifying therapies^45^ indicated that there is no difference in cancer risk in MS patients receiving this type of treatment compared to the general population.

In this study, strict inclusion criteria were defined and applied to minimize heterogeneity between included studies, which were extracted from all relevant databases. A series of study-specific characteristics were collected and tested to obtain additional insights, improve the understanding of the included studies, and explore specific mechanisms in the heterogeneity of results. All studies were assessed for reliability using the ROBINS-E tool. The comprehensive bias assessment, in which various sources of bias were analyzed and adjusted for, suggested robust results.

However, it should be noted that some of the associations found in our study may be spurious. It cannot be ruled out that treatment effects or other factors are responsible for some of the associations found. Based on the information from the included studies, it was not possible to evaluate the effects of autoimmune therapies on cancer risk. However, this question warrants evaluation in further studies. The restriction to English or German language might miss some studies (though major databases were covered). We did not contact authors for unpublished data, so publication bias might influence results despite our statistical corrections. Finally, given the difficulties in non-experimental studies investigating long-term relationships of low-prevalent diseases, most of the studies examined suffered from a serious risk of bias (especially due to unmeasured confounding) that could not be accounted for. Therefore, some of the results may have been overestimated due to residual bias.

The present findings are important for physicians treating patients with CD, SLE, MS and T1D as they provide an indication of a potential development of certain digestive system cancers that should be focused on preventively as the disease progresses. There is a need to identify patients who are at higher risk for developing digestive system cancers and who would benefit most from individualized screening programs. The results could possibly be considered in clinical guidelines for the autoimmune diseases investigated. Finally, it is important to sensitize both patients with an autoimmune disease and the public to the increased cancer risk and to inform them about preventive options, such as screening measures.

Our findings support the hypothesis of chronic inflammation (from autoimmunity) driving malignancy in specific organs, consistent with the literature on chronic inflammation and cancer. The results of the present study are valuable for healthcare professionals, as they highlight the importance of regularly monitoring patients with celiac disease, SLE, MS, and T1D and conducting individualized cancer screening, respectively. Further research is needed to clarify the role of treatment options in patients with the mentioned autoimmune diseases (e.g. immunosuppressive therapy, disease-modifying agents) regarding the risk of digestive system cancers.

## Contributors

JR: Investigation, Writing—Original draft. CM: Conceptualization, Investigation, Supervision, Writing—Reviewing and Editing. DF: Investigation, Methodology, Formal analysis, Visualization, Writing—Original draft, Funding acquisition, Project administration. SF: Investigation, Writing—Reviewing and Editing. JL: Resources, Supervision, Writing—Reviewing and Editing. A medical writer or editor was not involved in the creation of the manuscript. JR and DF have verified the underlying data. All authors read and approved the final version of the manuscript.

## Data sharing statement

This study was based on previously published data and is therefore available in the original studies. The study protocol was registered at PROSPERO under the registration ID: CRD42024553216. The source code used in this study is available from the corresponding author upon reasonable request.

## Declaration of interests

The authors declare that they have no competing interests.
