# Supplementary material for: Evaluating the risk of digestive system cancer in autoimmune disease patients: a systematic review and meta-analysis focusing on bias assessment
Source: eClinicalMedicine. 2025 Aug 7;87:103410. doi: 10.1016/j.eclinm.2025.103410 (PMC12355593; doi:10.1016/j.eclinm.2025.103410)
Supplement: Supplementary Figures and Tables [file mmc1.pdf]

Evaluating the risk of digestive system cancer in autoimmune disease patients: A systematic review and meta-analysis focusing on bias assessment

First author: Julia Reizner

Table of contents

Supplementary Tables	
Table 1	Database-specific search strings
Table 2	Study characteristics of included studies
Table 3	Studies excluded in the full-text screening
Table 4	Results from meta-regression analyses
Table 5	Heterogeneity statistics and publication bias
Table 6	Bias-corrected estimates adjusted for all identified sources of bias

Supplementary References	
References 1	Studies considered in this study

Supplementary Figures	
Figure 1	Study-specific risk of bias assessment
Figure 2	Distribution of studies regarding the risk of bias
Figure 3	Unadjusted meta-analyses regarding celiac disease as exposure
Figure 4	Unadjusted meta-analyses regarding SLE as exposure
Figure 5	Unadjusted meta-analyses regarding multiple sclerosis as exposure
Figure 6	Unadjusted meta-analyses regarding type 1 diabetes as exposure
Figure 7	Predictor importance derived from multi-model inference
Figure 8	Outlier-adjusted meta-analyses regarding celiac disease as exposure
Figure 9	Influence analyses regarding celiac disease as exposure
Figure 10	Subgroup analyses regarding celiac disease as exposure
Figure 11	Outlier-adjusted meta-analyses regarding SLE as exposure
Figure 12	Influence analyses regarding SLE as exposure
Figure 13	Subgroup analyses regarding SLE as exposure
Figure 14	Outlier-adjusted meta-analyses regarding multiple sclerosis as exposure
Figure 15	Influence analyses regarding multiple sclerosis as exposure
Figure 16	Outlier-adjusted meta-analyses regarding type 1 diabetes as exposure
Figure 17	Influence analyses regarding type 1 diabetes as exposure
Figure 18	Subgroup analyses regarding type 1 diabetes as exposure
Figure 19	Associations corrected for small-study bias via the fill-and-trim method

Supplementary Tables

Table 1: Search strings tailored to four databases and the number of extracted studies

Database	Hits	Search string
PubMed	5033	(celiac disease[MeSH Terms] OR celiac disease[Title/Abstract] OR coeliac disease[Title/Abstract] OR endemic sprue[Title/Abstract] OR celiac sprue[Title/Abstract] OR nontropical sprue[Title/Abstract] OR diabetes mellitus, type 1[MeSH Terms] OR diabetes mellitus, type 1[Title/Abstract] OR insulin dependent diabetes[Title/Abstract] OR type 1 diabetes[Title/Abstract] OR type i diabetes[Title/Abstract] OR type one diabetes[Title/Abstract] OR T1D[Title/Abstract] OR juvenile diabetes[Title/Abstract] OR multiple sclerosis[MeSH Terms] OR multiple sclerosis[Title/Abstract] OR encephalomyelitis disseminata[Title/Abstract] OR multiple cerebro-spinal sclerosis[Title/Abstract] OR multiple cerebral sclerosis[Title/Abstract] OR lupus erythematosus, systemic[MeSH Terms] OR lupus erythematosus[Title/Abstract]) AND ((gastrointestinal neoplasms[MeSH Terms] OR pancreatic neoplasms[MeSH Terms] OR liver neoplasms[MeSH Terms] OR colorectal neoplasms[MeSH Terms] OR colonic neoplasms[MeSH Terms] OR intestinal neoplasms[MeSH Terms] OR stomach neoplasms[MeSH Terms] OR esophageal neoplasms[MeSH Terms]) OR ((gastrointestinal[Title/Abstract] OR pancrea*[Title/Abstract] OR liver[Title/Abstract] OR hepat*[Title/Abstract] OR colo*[Title/Abstract] OR intestinal[Title/Abstract] OR bowel[Title/Abstract] OR stomach[Title/Abstract] OR gastric[Title/Abstract] OR esophag*[Title/Abstract] OR oesophag*[Title/Abstract]) AND (neoplasm*[Title/Abstract] OR cancer*[Title/Abstract] OR carcinom*[Title/Abstract] OR tumor*[Title/Abstract] OR malignanc*[Title/Abstract])))
Web of Science	3364	(TS="celiac disease" OR TS="coeliac disease" OR TS="endemic sprue" OR TS="celiac sprue" OR TS="nontropical sprue" OR TS="diabetes mellitus, type 1" OR TS="insulin dependent diabetes" OR TS="type 1 diabetes" OR TS="type i diabetes" OR TS="type one diabetes" OR TS=TD1 OR TS="juvenile diabetes" OR TS="multiple sclerosis" OR TS="encephalomyelitis disseminata" OR TS="multiple cerebro-spinal sclerosis" OR TS="multiple cerebral sclerosis" OR TS="lupus erythematosus") AND (TS=((gastrointestinal OR pancrea* OR liver OR hepat* OR colo* OR intestinal OR bowel OR stomach OR gastric OR esophag* OR oesophag*) NEAR/40 (neoplasm* OR cancer* OR carcinom* OR tumo$r* OR malignanc*)))
EMBASE (via Ovid)	11376	(exp "celiac disease"/ or exp "insulin dependent diabetes mellitus"/ or exp "multiple sclerosis"/ or exp "systemic lupus erythematosus"/ or (celiac disease or coeliac disease or endemic sprue or celiac sprue or nontropical sprue or insulin dependent diabetes or diabetes mellitus type 1 or type 1 diabetes or type i diabetes or type one diabetes or T1D or juvenile diabetes or multiple sclerosis or encephalomyelitis disseminata or multiple cerebro-spinal sclerosis or multiple cerebral sclerosis or lupus erythematosus).ab,kf,ti.) and (exp gastrointestinal tumor/ or exp pancreas tumor/ or exp liver tumor/ or exp colorectal tumor/ or exp colon tumor/ or exp intestine tumor/ or exp stomach tumor/ or exp esophagus tumor/ or ((gastrointestinal or pancrea* or liver or hepat* or colo* or intestin* or bowel or stomach or gastric or esophag* or oesophag*) adj40 (neoplasm* or cancer* or carcinoma* or tumo?r* or malignanc*)).ab,kf,ti.)
Cochrane	920	ID Search<br>#1 MeSH descriptor: [Celiac Disease] explode all trees<br>#2 MeSH descriptor: [Diabetes Mellitus, Type 1] explode all trees<br>#3 MeSH descriptor: [Multiple Sclerosis] explode all trees<br>#4 MeSH descriptor: [Lupus Erythematosus, Systemic] explode all trees

		#5 (celiac disease):ti,ab,kw
		#6 ("coeliac diseases"):ti,ab,kw
		#7 (endemic sprue):ti,ab,kw
		#8 (celiac sprue):ti,ab,kw
		#9 (nontropical sprue):ti,ab,kw
		#10 ("diabetes mellitus type 1"):ti,ab,kw
		#11 ("type 1 diabetes"):ti,ab,kw
		#12 ("type i diabetes"):ti,ab,kw
		#13 (type one diabetes):ti,ab,kw
		#14 (T1D):ti,ab,kw
		#15 ("juvenile diabetes"):ti,ab,kw
		#16 (multiple sclerosis):ti,ab,kw
		#17 (encephalomyelitis disseminata):ti,ab,kw
		#18 (multiple cerebro-spinal sclerosis):ti,ab,kw
		#19 (multiple cerebral sclerosis):ti,ab,kw
		#20 ("lupus erythematosus"):ti,ab,kw
		#21 MeSH descriptor: [Gastrointestinal Neoplasms] explode all trees
		#22 MeSH descriptor: [Pancreatic Neoplasms] explode all trees
		#23 MeSH descriptor: [Liver Neoplasms] explode all trees
		#24 MeSH descriptor: [Colorectal Neoplasms] explode all trees
		#25 MeSH descriptor: [Colonic Neoplasms] explode all trees
		#26 MeSH descriptor: [Intestinal Neoplasms] explode all trees
		#27 MeSH descriptor: [Stomach Neoplasms] explode all trees
		#28 MeSH descriptor: [Esophageal Neoplasms] explode all trees
		#29 (gastrointestinal):ti,ab,kw
		#30 (pancrea*):ti,ab,kw
		#31 (liver):ti,ab,kw
		#32 (hepat*):ti,ab,kw
		#33 (colo*):ti,ab,kw
		#34 (intestin*):ti,ab,kw
		#35 (bowel):ti,ab,kw
		#36 (stomach):ti,ab,kw
		#37 (gastric):ti,ab,kw
		#38 (esophag*):ti,ab,kw
		#39 (oesophag*):ti,ab,kw
		#40 (neoplasm*):ti,ab,kw
		#41 (cancer*):ti,ab,kw
		#42 (carcinoma*):ti,ab,kw
		#43 (tumo?r*):ti,ab,kw
		#44 (malignanc*):ti,ab,kw
		#45 (insulin dependent diabetes):ti,ab,kw
		#46 #1 OR #2 OR #3 OR #4 OR #5 OR #6 OR #7 OR #8 OR #9 OR #10 OR #12 OR #13 OR #14 OR #15 OR #16 OR #17 OR #18 OR #19 OR #20 OR #45
		#47 #21 OR #22 OR #23 OR #24 OR #25 OR #26 OR #27 OR #28
		#48 #29 OR #30 OR #31 OR #32 OR #33 OR #34 OR #35 OR #36 OR #37 OR #38 OR #39
		#49 #40 OR #41 OR #42 OR #43 OR #44
		#50 #48 near/40 #49
		#51 #47 OR #50
		#52 #46 AND #51

Table 2: Study characteristics of included studies

First author (year)	Country/ Region	Study design (duration)	Exposure (cases)	Outcome (cases)	ICD [version] Code	Comparison group	Effect estimate	Adjustment variables
J. Askling (2002) (1)	Sweden	Cohort study (1964 - 1995)	Celiac disease (11019)	Esophagus (6)<br>Stomach (6)<br>Small intestine (8)<br>Large intestine (26)<br>Rectum (6)<br>Primary liver (11)<br>Pancreas (9)	[7]: 150<br>[7]: 151<br>[7]: 152<br>[7]: 153<br>[7]: 154<br>[7]: 155<br>[7]: 157	Swedish population	SIR	age, sex, calendar period
S. Bernatsky (2013) (2)	Transethnic	Cohort study (1958 - 2009)	Lupus (16409)	Pancreas (10)<br>Liver (12)<br>Gastric (14)<br>Colorectal (51)	-	Transethnic population	SIR	geographically dependent age, sex, calendar period
M. Buttmann (2016) (3)	Germany	Cohort study (1994 - 2011)	MS (677)	Colorectal (7)	-	German population	SIR	age, sex, year of occurrence
B. Carstensen (2016) (4)	Transethnic	cohort study (1972 - 2012)	Type I diabetes (9149)	Women:<br>Esophagus (30)<br>Stomach (254)<br>Colon (483)<br>Rectum (272)<br>Colorectal (876)<br>Liver (154)<br>Pancreas (240)<br><br>Men:<br>Esophagus (67)<br>Stomach (134)<br>Colon (273)<br>Rectum (158)<br>Colorectal (492)<br>Liver (113)<br>Pancreas (147)	 [10]: C15<br>[10]: C16<br>[10]: C18<br>[10]: C20<br>[10]: C18-20<br>[10]: C22<br>[10]: C25<br><br><br>[10]: C15<br>[10]: C16<br>[10]: C18<br>[10]: C20<br>[10]: C18-20<br>[10]: C22<br>[10]: C25	Australian population, Danish population, Finish population, Scottish population, and Swedish populations	HR	age, calendar time

First author (year)	Country/ Region	Study design (duration)	Exposure (cases)	Outcome (cases)	ICD [version] Code	Comparison group	Effect estimate	Adjustment variables
F. A. Castro (2014) (5)	Sweden	cohort study (1964 - 2008)	Celiac disease (402462)<br>Lupus (402462)<br>MS (402462)	Liver (9)<br>Liver (17)<br>Liver (9)	[7]: 155<br>[7]: 155<br>[7]: 155	Swedish population	HR	age, sex, calendar period, region, socioeconomic status, smoking, alcoholism
Y. J. Chen (2010) (6)	Taiwan	cohort study (1996 - 2007)	Lupus (11763)	Liver and gallbladder (28)<br>Colorectal (14)<br>Stomach (14)<br>Esophagus (2)<br>Pancreas (4)	[9]: 155,156<br>[9]: 153,154<br>[9]: 151<br>[9]: 150<br>[9]: 157	Taiwanese population	SIR	age, sex, follow-up duration
D. Dey (2013) (7)	England	nested case-control (1978 - 2010)	Lupus (595)	Pancreas (1)	-	English population	SIR	age, sex
P. Elfström (2012) (8)	Sweden	cohort study (1989 - 2007)	Celiac disease (28882)	Esophagus (8)<br>Stomach (24)<br>Small intestine (15)<br>Colon (88)<br>Rectum (30)<br>Liver (39)<br>Pancreas (38)	[7]: 150<br>[7]: 151<br>[7]: 152<br>[7]: 153<br>[7]: 154<br>[7]: 155<br>[7]: 157	Swedish population	HR	age, sex, county, calendar year, education
L. Emilsson (2020) (9)	Sweden	cohort study (1965 - 2017)	Celiac disease (48119)	Small bowel adenocarcinoma (29)	[10]: C17	Swedish population	HR	age, sex, county, calendar year, education
A. F. Fois (2010) (10)	England	record linkage study (1963 - 1999)	MS (4250)	Stomach (14)<br>Colon (13)<br>Rectum (11)<br>Liver (6)<br>Pancreas (5)	[9]: 151<br>[9]: 153<br>[9]: 154<br>[9]: 155<br>[9]: 157	Patients without MS	RR	age, sex, calendar year, district of residence
M. J. Goldacre (2008) (11)	England	record linkage study (1963 - 1999)	Celiac disease (1997)	Esophagus (5)<br>Stomach (8)<br>Colon (11)<br>Rectum (4)<br>Pancreas (2)	[9]: 150<br>[9]: 151<br>[9]: 153<br>[9]: 154<br>[9]: 157	Patients without celiac disease	RR	age, sex, calendar year, district of residence

**First author (year)**	**Country/ Region**	**Study design (duration)**	**Exposure (cases)**	**Outcome (cases)**	**ICD [version] Code**	**Comparison group**	**Effect estimate**	**Adjustment variables**
P. Gomez-Rubio (2019) (12)	Spain, UK, Germany, Ireland, Sweden, Italy	case-control study (2009 - 2014)	Lupus (12)<br>Celiac disease (13)	Pancreas (1705)<br>Pancreas (1705)	-	European patients without the AD	OR	age, sex, country, smoking, alcohol, t2d, obesity, family history of pancreatic cancer, education, treatment
J. Y. Han (2021) (13)	Korea	cohort study (2012 - 2015)	Lupus (17854)	Liver (63)<br>Colon (61)<br>Pancreas (32)<br>Stomach (31)	[10]: C22<br>[10]: C18<br>[10]: C25<br>[10]: C16	Korean population	SIR	age, sex, calendar period
J. L. Harding (2015) (14)	Australia	cohort study (1997 - 2008)	Type I diabetes (80676)	Esophagus (30)<br>Stomach (40)<br>Gallbladder (6)<br>Colorectal (237)<br>Liver (53)<br>Pancreas (67)	[10]: C15<br>[10]: C16<br>[10]: C23<br>[10]: C18-20<br>[10]: C22<br>[10]: C25	Australian population	SIR	age, sex, calendar year
M. M. He (2022) (15)	England	cohort study (2006 - 2010)	Celiac disease (2273)	Small intestine (3)<br>Colorectal (9)<br>Pancreas (3)<br>Esophagus (4)	[10]: C17<br>[10]: C18-20<br>[10]: C25<br>[10]: C15	British population	HR	age, sex, ethnicity, socioeconomic status, education, physical activity, BMI, waist-to-hip ratio, height, smoking, alcohol
			Type I diabetes (3556)	Stomach (4)<br>Colorectal (28)<br>Pancreas (3)<br>Esophagus (9)<br>Liver (10)	[10]: C16<br>[10]: C18-20<br>[10]: C25<br>[10]: C15<br>[10]: C22			
			MS (1610)	Stomach (2)<br>Colorectal (6)<br>Liver (1)	[10]: C16<br>[10]: C18-20<br>[10]: C22			
			Lupus (611)	Liver (1)<br>Colorectal (4)<br>Esophagus (1)	[10]: C22<br>[10]: C18-20<br>[10]: C15			

First author (year)	Country/ Region	Study design (duration)	Exposure (cases)	Outcome (cases)	ICD [version] Code	Comparison group	Effect estimate	Adjustment variables
K. Hemminki (2012) (16)	Sweden	cohort study (1964 - 2008)	Celiac disease (4124)<br><br><br><br><br><br><br>Lupus (5318)<br><br><br><br><br><br>Type I diabetes (20554)<br><br><br><br><br>MS (12553)	Upper digestive tract (1)<br>Esophageal SCC (1)<br>Small intestine (1)<br>Colon (9)<br>Rectum (1)<br><br>Upper digestive tract (16)<br>Esophageal SCC (2)<br>Stomach (10)<br>Colon (38)<br>Rectum (10)<br><br>Upper digestive tract (5)<br>Esophageal SCC (1)<br>Stomach (5)<br>Colon (4)<br>Rectum (5)<br><br>Upper digestive tract (19)<br>Esophageal adenocarcinoma (2)<br>Stomach (12)<br>Small intestine (2)<br>Colon (62)<br>Rectum (23)	-	Swedish population	SIR	age, sex, calendar period, region, socioeconomic status, smoking, alcoholism, obesity
K. Hemminki (2023) (17)	Sweden	cohort study (1997 - 2018)	Celiac disease (52373)	Men:<br>Liver (11)<br>Women:	[10]: C22	Swedish population	SIR	age, sex, calendar period, education, region

First author (year)	Country/ Region	Study design (duration)	Exposure (cases)	Outcome (cases)	ICD [version] Code	Comparison group	Effect estimate	Adjustment variables
			 <br><br><br>Type I diabetes (143701)<br><br><br><br><br><br><br>Lupus (9142)	Liver (9)<br>Gallbladder (6)<br><br><br>Men:<br>Liver (376)<br>Gallbladder (28)<br>Women:<br>Liver (89)<br>Gallbladder (49)<br><br>Men:<br>Liver (5)<br>Women:<br>Liver (9)<br>Gallbladder (7)	[10]: C22<br>[10]: C23.9<br><br><br><br>[10]: C22<br>[10]: C23.9<br><br>[10]: C22<br>[10]: C23.9<br><br><br>[10]: C22<br><br>[10]: C22<br>[10]: C23 .9			
P. C. Hsu (2015) (18)	Taiwan	cohort study (2000 - 2008)	Type I diabetes (14619)	Men:<br>Stomach (17)<br>Colon (31)<br>Rectum (27)<br>Liver (92)<br>Pancreas (12)<br><br>Women:<br>Stomach (14)<br>Colon (39)<br>Rectum (15)<br>Liver (30)<br>Pancreas (16)	 [9]: 151<br>[9]: 153<br>[9]: 154<br>[9]: 155<br>[9]: 157<br><br><br>[9]: 151<br>[9]: 153<br>[9]: 154<br>[9]: 155<br>[9]: 157	Taiwanese population	SIR	age, sex, calendar year
T. Ilus (2014) (19)	Finland	cohort study (2002 - 2011)	Celiac disease (32439)	Small intestine (27)<br>Colon (133)<br>Rectum (51)<br>Esophagus (22)	-	Finnish population	SIR	age, sex, calender period

First author (year)	Country/ Region	Study design (duration)	Exposure (cases)	Outcome (cases)	ICD [version] Code	Comparison group	Effect estimate	Adjustment variables
				Stomach (37)<br>Liver (24)<br>Pancreas (45)				
S. Kariniemi (2022) (20)	Finland	case-control study (2000 - 2018)	Lupus (1006)	Colorectal (8)<br>Pancreas (5)	[10]: C18-20<br>[10]: C25	Finnish population	IRR	matched by age, sex, place of residence
E. Kingwell (2012) (21)	Canada	cohort study (1980 - 2007)	MS (6917)	Colorectal (28)<br>Stomach (1)<br><br>Women:<br>Esophagus (1)<br>Pancreas (5)<br>Men:<br>Esophagus (1)<br>Pancreas (5)	-	Canadian population	SIR	age, sex, calendar year
I. Koskinen (2021) (22)	Finland	cohort study (1960 - 2014)	Celiac disease (1460)	Esophagus (3)<br>Stomach (11)<br>Small intestine (1)<br>Colon (14)<br>Rectum (9)<br>Liver (7)<br>Pancreas (7)	[O-3]: C15<br>[O-3]: C16<br>[O-3]: C17<br>[O-3]: C18<br>[O-3]: C20<br>[O-3]: C22<br>[O-3]: C25	Population of Tampere	SIR	age, sex, calender period
A. Krishnan (2023) (23)	USA	cohort study (2005 - 2022)	Celiac disease (134680)	Pancreas (151)	[10]: C25	Patients without CD	HR	Age, sex, race/ethnicity (Hispanic, non-Hispanic white, non-Hispanic black, or non-Hispanic other), nicotine dependence, alcohol-related disease, body mass index (BMI), type 2 diabetes mellitus (T2DM), hypertension, hyperlipidemia, type 1 diabetes, autoimmune thyroid disease, rheumatoid arthritis, inflammatory

First author (year)	Country/ Region	Study design (duration)	Exposure (cases)	Outcome (cases)	ICD [version] Code	Comparison group	Effect estimate	Adjustment variables
								bowel disease, disease of gallbladder, diseases of the pancreas, hypercholesterolemia, hypercalcemia, familial hypercholesterolemia, family history of primary malignant neoplasm, ERCP, cholecystectomy, cholesterol level, low-density lipoprotein, and serum triglyceride level, hemoglobin A1C (HbA1C) and genomics
A. M. Landgren (2011) (24)	USA	cohort study (1969 - 1996)	Celiac disease<br><br><br><br><br>Lupus<br><br><br><br><br><br>MS	Esophagus (11)<br>Colon (11)<br>Rectum (9)<br>Pancreas (13)<br><br>Esophagus (8)<br>Stomach (8)<br>Colon (19)<br>Rectum (6)<br>Pancreas (7)<br><br>Colon (72)<br>Rectum (37)<br>Liver (7)<br>Pancreas (18)<br>Esophagus (14)<br>Stomach (18)	[9]: 150<br>[9]: 153<br>[9]: 154<br>[9]: 157<br><br>[[9]: 150<br>[9]: 151<br>[9]: 153<br>[9]: 154<br>[9]: 157<br><br>[9]: 153<br>[9]: 154<br>[9]: 155<br>[9]: 157<br>[9]: 150<br>[9]: 151	Male veterans without the AD	RR	age, calendar year, race, time between study entry and exit, alcoholism
B. Lebwohl (2022) (25)	Sweden	cohort study (1965 - 2016)	Celiac disease (47241)	Gastric (65)<br>Colorectal (448)<br>Liver and gallbladder (115)<br>Pancreas (152)	[7]: 151<br>[7]: 153-154<br>[7]: 155-156<br><br>[7]: 157	Swedish population	HR	age, sex, calendar year, and county, education

First author (year)	Country/ Region	Study design (duration)	Exposure (cases)	Outcome (cases)	ICD [version] Code	Comparison group	Effect estimate	Adjustment variables
J. A. Liang (2012) (26)	Taiwan	cohort study (1996 - 2008)	Lupus (2150)	Colorectal<br>Liver	[9]: 153,154<br>[9]: 155	Taiwanese population	HR	age, sex, area, occupation, urbanization, income
R. A. Marrie (2021) (27)	Canada	cohort study (1984 - 2018)	MS (53983)	Colorectal	[9]: 153,154	Canadian population	HR	age at index date, socioeconomic status, region, Elixhauser comorbidity score, and birth cohort year
L. Mellemkjaer (1997) (28)	Denmark	cohort study (1977 - 1991)	Lupus (1585)	Esophagus (1)<br>Stomach (2)<br>Colorectal (10)<br>Liver (5)<br>Pancreas (1)	[7]: 150<br>[7]: 151<br>[7]: 153-154<br>[7]: 155<br>[7]: 157	Danish population	SIR	sex, age, and calendar time
A. Parikh-Patel (2008) (29)	USA	cohort study (1991 - 2002)	Lupus (30478)	Esophagus (12)<br>Stomach (18)<br>Colorectal (99)<br>Liver (17)<br>Pancreas (25)	[9]: 150<br>[9]: 151<br>[9]: 153-154<br>[9]: 155<br>[9]: 157	California population	SIR	age, sex,
C. Pierret (2024) (30)	French	cohort study (2012-2021)	MS (140649)	Colorectal (4376)	[10]: C18-20	French population	HR	Blood and stool testing
S. Shin (2024)(31)	Korea	cohort study (2005-2019)	Type I diabetes (2681)	Gastric (101)<br>Colon (100)<br>Liver (90)<br>Pancreas (31)<br>Esophagus (12)	[10]: C16<br>[10]: C18-20<br>[10]: C22<br>[10]: C25<br>[10]: C15	Korean population	HR	age, sex, BMI, systolic BP, fasting blood glucose, total cholesterol, GGT, smoking status, alcohol consumption,<br>physical activity and household income
X. Shu (32)(2010)	Sweden	cohort study (1964 - 2006)	Type I diabetes (24052)	Stomach (8)<br>Colon (10)<br>Rectum (5)<br>Liver (3)<br>Pancreas (2)	[7]: 151<br>[7]: 153<br>[7]: 154<br>[7]: 155<br>[7]: 157	Swedish population	SIR	sex, age at first hospitalization, period of diagnosis and region
A. J. Swerdlow (2023) (33)	UK	cohort study (1972 - 2018)	Type I diabetes (23473)	Esophagus (21)<br>Stomach (22)<br>Small intestine (4)<br>Colorectal (77)	[9]: 150<br>[9]: 151<br>[9]: 152<br>[9]: 153-154	British population	SIR	age, sex, country

First author (year)	Country/ Region	Study design (duration)	Exposure (cases)	Outcome (cases)	ICD [version] Code	Comparison group	Effect estimate	Adjustment variables
				Liver (5)<br>Gallbladder (2)<br>Pancreas (14)	[9]: 155<br>[9]: 156<br>[9]: 157			
E. Treppo (2022) (34)	Italy	cohort study (2002 - 2017)	Lupus (866)	Stomach (3)<br>Liver and gallbladder (1)<br>Pancreas (3)	[10]: C16<br>[10]: C22-24<br><br>[10]: C25	Italian population	SIR	age, sex
F. Valent (2015) (35)	Italy	cohort study (2002 - 2014)	Type I diabetes (6728)	Esophagus<br>Stomach<br>Small intestine<br>Colon<br>Rectum<br>Liver<br>Gallbladder<br>Pancreas	[9]: 150<br>[9]: 151<br>[9]: 152<br>[9]: 153<br>[9]: 154<br>[9]: 155<br>[9]: 156<br>[9]: 157	Italian population	HR	age, sex
M. Vicentini (2022) (36)	Italy	cohort study (2009 - 2016)	Type I diabetes (758)	Colorectal (1)<br>Liver (1)	[10]: C18-20<br>[10]: C22	Italian population	IRR	age, sex, citizenship
M. Viljamaa (2006) (37)	Finland	cohort study (1960 - 2002)	Celiac disease (781)	Digestive system (10)<br>Stomach (2)<br>Colorectal (4)	-	Finnish population	SIR	age, sex, calendar period
R. Westermann (2021) (38)	Denmark	cohort study (1995 - 2014)	Lupus (3424)	Esophagus (7)<br>Colorectal (21)<br>Liver (4)<br>Pancreas (12)	[10]: C15<br>[10]: C20<br>[10]: C22<br>[10]: C25	Danish population	SIR	age, sex, calendar time period
F. Yuan (2022) (39)	USA	case-control study (1992 - 2015)	Celiac disease (341)<br>MS (502)<br>Lupus (963)	Pancreas (90)<br>Pancreas (114)<br>Pancreas (285)	[10]: C25<br>[10]: C25<br>[10]: C25<br>[10]: C25	American population	OR	age, sex, calendar year, race, …
K. Zendehdel (2003) (40)	Sweden	cohort study (1965 - 1999)	Type I diabetes (29187)	Stomach (10)<br>Colorectal (18)<br>Pancreas (3)	[7]: 151<br>[7]: 153,154<br>[7]: 157	Swedish population	SIR	age, sex, calendar year

First author (year)	Country/ Region	Study design (duration)	Exposure (cases)	Outcome (cases)	ICD [version] Code	Comparison group	Effect estimate	Adjustment variables
M. Abu-Shakra (1996) (41)	Canada	cohort study (1970 - 1994)	Lupus (724)	Colon (3)<br>Pancreas (2)	-	Canadian population	SIR	age
L. Bjornadal (2002) (42)	Sweden	cohort study (1964 - 1995)	Lupus (5715)	Esophagus (5)<br>Stomach (19)<br>Colon (31)<br>Rectum (20)<br>Liver (17)<br>Pancreas (15)	[7]: 150<br>[7]: 151<br>[7]: 153<br>[7]: 154<br>[7]:155<br>[7]:157	Swedish population	SIR	age, sex, calendar period
M. J. Grainge (2012) (43)	UK	cohort study (1970 - 2004)	Celiac disease (435)	Esophagus (3)<br>Colorectal (6)<br>Small intestine (1)	[10]:C15<br>[10]:C18-20<br>[10]:C17	Scottish population	SIR	age, sex
J. Cibere (2001) (44)	Canada	cohort study (1975 - 1994)	Lupus (297)	Colorectal (1)<br>Liver and gallbladder (1)<br>Pancreas (1)	-	Canadian population	SIR	age, sex, calendar year
G. G. n. O´ Ragnarsson (2003) (45)	Iceland	cohort study (1957 - 2001)	Lupus (238)	Colon (1)	-	Icelandic population	SIR	age, sex, calendar period
M. S. Bahmanyar (2009) (46)	Sweden	cohort study (1958 - 2005)	MS (20276)	Digestive system (352)<br>Esophagus (12)<br>Stomach (34)<br>Small intestine (16)<br>Colon (143)<br>Rectum (72)<br>Liver (3)<br>Pancreas (32)	[7]: 150-159<br><br>[7]: 150<br>[7]: 151<br>[7]: 152<br>[7]:153<br>[7]:154<br>[7]:156<br>[7]:157	Swedish population	HR	age, sex, vital status, region
A. J. Swerdlow (2005) (47)	UK	cohort study (1972 - 2001)	Type I diabetes (23834)	Esophagus (4)<br>Stomach (7)<br>Colorectal (14)<br>Liver (1)<br>Pancreas (5)	[9]: 150<br>[9]: 151<br>[9]: 153, 153<br>[9]:155<br>[9]:157	British population	SIR	age, sex, calendar year, country

Table 3: Studies excluded within the full-text screening

First author	Year	Titel	Reasons for exclusion
A. Abdalla	2020	Does high disease activity in systemic lupus erythematosus patients increase the risk of cancer incidence?	Conference abstract
A. M. Abdalla	2019	Risk of cancer incidence among patients with systemic lupus erythematosus (SLE)	Conference abstract
D. Abrams	2021	THE ASSOCIATION BETWEEN CHRONIC AND NEW-ONSET DIABETES AND PANCREATIC CANCER CARE OUTCOMES IN THE NATIONWIDE VETERANS AFFAIRS HEALTHCARE SYSTEM	Conference abstract
M. Abureesh	2020	SYSTEMIC LUPUS ERYTHEMATOUS AND COLORECTAL CANCER: THE RISK ASSOCIATION AND EFFECT OF MEDICATIONS	Conference abstract
G. Y. Ahn	2020	Increased risk of mortality and cancer in systemic lupus erythematosus: Results from a lupus cohort study from 1998 to 2015	Conference abstract
V. Ajdacic-Gross	2016	Cancer co-occurrence patterns in Parkinson's disease and multiple sclerosis-Do they mirror immune system imbalances?	Mortality as outcome/ wrong outcome
A. R. Al Armashi	2022	Trends in ethnic-specific colorectal cancer mortality in patients with and without diabetes in the United States: A CDC database population-based study 2011-2019	Mortality as outcome / wrong outcome
B. Al-Bawardy	2017	Celiac disease: a clinical review	Clinical review
S. Ali	2024	Association between unstable diabetes mellitus and risk of pancreatic cancer	stable vs. unstable diabetes groups /wrong exposure
H. Allgayer	2008	[Celiac sprue and malignancies: analysis of risks and prevention strategies]	Review
D. K. Andersen	2013	Diabetes and cancer: placing the association in perspective	Review
Anonymous	2010	Cancer risk in diabetic patients treated with insulin glargine?	Review
T. Aparicio	2020	Small bowel adenocarcinoma: Results from a nationwide prospective ARCAD-NADEGE cohort study of 347 patients	Only small bowel adenocacinoma patients/ wrong outcome
T. Aparicio	2022	Epidemiology, Risk Factors and Diagnosis of Small Bowel Adenocarcinoma	Review
T. Aparicio	2014	Small bowel adenocarcinoma: epidemiology, risk factors, diagnosis and treatment	Review
N. Ari	2023	Gastrointestinal tract lymphomas: A retrospective analysis	Conference abstract
G. Arpa	2023	Early-onset small bowel adenocarcinomas (EO-SBAs) are more frequently associated with a predisposing condition compared to late-onset SBAs: An international multicentre study	Conference abstract
M. Askari	2022	Incidence of cancer in patients with multiple sclerosis (MS) who were treated with fingolimod: A systematic review and meta-analysis	Systematic review and meta-analysis
O. Babajide	2023	EXOCRINE PANCREAS INSUFFICIENCY, PREDIABETES, DIABETES MELLITUS, AND THE INCIDENCE OF PANCREATIC ADENOCARCINOMA: A POPULATION-LEVEL ENRICHMENT STUDY	Conference abstract
J. Badenhorst	2014	Coeliac disease	wrong study type
O. Bahri	2011	First multicenter study for risk factors for hepatocellular carcinoma development in North Africa	T2D/ wrong exposure
V. Bain	2013	Malignancies in systemic lupus erythematosus: Early appearance in the course of the disease	Conference abstract
P. Ballotari	2017	Diabetes and risk of cancer incidence: results from a population-based cohort study in northern Italy	Mixed diabetes types/ wrong exposure
R. Bei	2009	A common repertoire of autoantibodies is shared by cancer and autoimmune disease patients: Inflammation in their induction and impact on tumor growth	Assessment of shared antibodies/ wrong study design
H. Benhammane	2012	Small bowel adenocarcinoma complicating coeliac disease: a report of three cases and the literature review	Review
E. Benjaminsen	2019	Comorbidity in patients with multiple sclerosis from Nordland, Norway	Conference abstract
E. Benjaminsen	2021	Comorbidity in multiple sclerosis patients from Nordland County, Norway - validated data from the Norwegian Patient Registry	Prevalences
S. Bernatsky	2005	An international cohort study of cancer in systemic lupus erythematosus	Excluded at data extraction
S. Bernatsky	2012	Systemic lupus and malignancies	Review
S. Bernatsky	2021	Cancer Risk in a Large Inception Systemic Lupus Erythematosus Cohort: Effects of Demographic Characteristics, Smoking, and Medications	Assessed exposures are sex, etc

S. Bernatsky	2017	Cancer in systemic lupus erythematosus: Results from the systemic lupus international collaborating clinics inception cohort	Conference abstract
S. Bernatsky	2016	Cancer in systemic lupus erythematosus: Results from the systemic lupus international collaborating clinics inception cohort	Conference abstract
S. Bernatsky	2016	Cancer in systemic lupus erythematosus: Results from the slicc inception cohort	Conference abstract
S. R. Bernatsky	2006	Cancer screening in patients with systemic lupus erythematosus	Cancer screening / wrong exposure
C. R. Boland	2005	Infection, inflammation, and gastrointestinal cancer	Review
P. Bosco-Lévy	2022	Incidence and risk of cancer among multiple sclerosis patients: A matched population-based cohort study	Excluded at data exctraction
Z. J. Bro	2020	Diabetes Mellitus as a Risk Factor of Interval Colon Cancer	Letter to the editor
N. Brousse	2005	Malignant complications of coeliac disease	wrong estimate
M. Z. Brufatto	2024	Childhood-onset systemic lupus erythematosus (cSLE) and malignancy: a nationwide multicentre series review	Review
M. Buysschaert	2013	Diabetes and cancer: A 2013 synopsis	Review
A. Buyukkurt	2023	The effect of multiple sclerosis on cancer risk: A Mendelian randomization study	Conference abstract
A. Buyukkurt	2023	The effect of multiple sclerosis on cancer risk: A Mendelian randomization study	Conference abstract
A. Buyukkurt	2023	Exploring Shared Genetic Risk Between Multiple Sclerosis and Common Cancers	Conference abstract
R. A. Cader	2018	Malignancy in Systemic Lupus Erythematosus (SLE) Patients	wrong estimate
G. Caio	2019	Small bowel adenocarcinoma as a complication of celiac disease: clinical and diagnostic features	wrong estimate
E. E. Calle	1998	Diabetes mellitus and pancreatic cancer mortality in a prospective cohort of United States adults	wrong outcome, Mortality
L. Cao	2015	Systemic lupus erythematous and malignancy risk: a meta-analysis	Meta-analysis, No distinction between malignancy types
T. R. Card	2004	Risk of malignancy in diagnosed coeliac disease: a 24-year prospective, population-based, cohort study	Excluded at data extraction
B. Carstensen	2014	Cancer occurrence in type 1 diabetes patients: A 4-country study with 8800 cancer cases in 3.7 mio person-years	Conference abstract
G. Cassano	2007	Accrual of organ damage over time in Argentine patients with systemic lupus erythematosus: a multi-centre study	wrong outcome
I. Casserly	1997	Carcinoma of the right side of the colon and celiac disease	Case report
F. Catalá-López	2014	Inverse and direct cancer comorbidity in people with central nervous system disorders: a meta-analysis of cancer incidence in 577,013 participants of 50 observational studies	Meta-analysis, Review
C. Catassi	2005	Association of celiac disease and intestinal lymphomas and other cancers	Review
S. Cereda	2006	Celiac disease and childhood cancer	Case report
S. A. Chambers	2009	Damage and mortality in a group of British patients with systemic lupus erythematosus followed up for over 10 years	Wrong outcome
L. Chan	2014	Small bowel cancer: A 20-year single UK centre experience	Conference abstract
E. Chantelau	1994	Geographical variation of type 1 diabetes mellitus and pancreatic carcinoma	Wrong outcome
L. Chen	2024	Exploring potential causal associations between autoimmune diseases and colorectal cancer using bidirectional Mendelian randomization	MR, wrong study design
H. C. Cheng	2021	Narrative review of the influence of diabetes mellitus and hyperglycemia on colorectal cancer risk and oncological outcomes	Review
C. C. Chiu	2013	Increased Risk of Gastrointestinal Malignancy in Patients with Diabetes Mellitus and Correlations with Anti-Diabetes Drugs: A Nationwide Population-based Study in Taiwan	T2D. No information about T1D/ wrong exposure
V. Chouhan	2017	The 2012 to 2017 rate of first ever colorectal cancer occurrence in individuals 75 years of age and older: A population-based national study	Conference abstract
P. Chretien-Raymer	2014	Anti-Ro antibodies and cancer in systemic lupus erythematosus	Conference abstract
D. H. Christensen	2016	Venous thromboembolism and risk of cancer in patients with diabetes mellitus	T2D/ wrong exposure
B. C. Chun	2005	Mortality and cancer incidence in Korean patients with systemic lupus erythematosus: Results from the Hanyang Lupus Cohort in Seoul, Korea	No distinction between cancer types/ wrong outcome
S. L. Cichosz	2024	Prediction of pancreatic cancer risk in patients with new-onset diabetes using a machine learning approach based on routine	T2D/ wrong exposure

		biochemical parameters; Prediction of Pancreatic Cancer Risk in New Onset Diabetes	
P. J. Ciclitira	2001	AGA technical review on celiac sprue	Technical Review
A. E. Clarke	2021	Risk of malignancy in patients with systemic lupus erythematosus: Systematic review and meta-analysis	Systematic review and meta-analysis
Y. Cui; D. K. Andersen	2012	Diabetes and pancreatic cancer	Review
J. M. de Miguel-Yanes	2011	Diabetes, Insulin Resistance, and Cancer: An Update	Review, T2D
K. Demiroren	2022	Possible relationship between refractory celiac disease and malignancies	Review
L. Deng	2012	Diabetes mellitus and the incidence of colorectal cancer: an updated systematic review and meta-analysis	Systematic review and meta-analysis, no distinction between T1D and T2D
I. D. Dey	2012	A nested case control study of the association between systemic lupus erythematosus and cancer	Conference abstract
W. Dickey	2002	Colon neoplasia co-existing with coeliac disease in older patients: coincidental, probably; important, certainly	wrong study design
V. Dostanko	2017	Co-morbidity status in system lupus erythematosus patients	Conference abstract
L. Dreyer	2011	High incidence of potentially virus-induced malignancies in systemic lupus erythematosus: a long-term followup study in a Danish cohort	Excluded at data extraction
E. Duff	2023	A single-centre retrospective study of the association between subacute cutaneous lupus erythematosus and malignancy	Conference abstract
E. Duff	2023	The association between subacute cutaneous lupus erythematosus and malignancy	Letter to the editor
M. L. Dupla	1993	Malignancy in systemic lupus erythematosus: A report of five cases in a series of 96 patients	Case report
B. E. Dutra	2021	CLINICAL CHARACTERISTICS AND OUTCOMES OF PATIENTS WITH POSITIVE CELIAC SEROLOGY AND CANCER THERAPY EXPOSURE	Conference abstract
G. Ebers	2011	Cause of death in multiple sclerosis patients from a 21-year long-term follow-up study	Conference abstract
G. Egiziano	2016	Malignancy in Systemic Lupus Erythematosus	Review
W. Eigner	2017	Dynamics of occurrence of refractory coeliac disease and associated complications over 25 years	wrong estimate
P. Elfström	2011	Risk of lymphoproliferative malignancy in relation to small intestinal histopathology among patients with celiac disease	wrong outcome
L. Elli	2012	Risk of intestinal lymphoma in undiagnosed coeliac disease: results from a registered population with different coeliac disease prevalence	wrong otucome
L. Emilsson	2016	Cancer in first-degree relatives of people with celiac disease	Gastrointestinal cancer as outcome
A. J. Farias	2019	Severe diabetes-related complications and pancreatic cancer incidence in the multiethnic cohort	Conference abstract
B. G. A. Fonseca	2018	Malignancy in childhood onset-systemic lupus erythematosus: Real-life data from a nationwide series	Conference abstract
D. Franco	2022	A multivariate logistic model to predict odds of small bowel cancer by various risk factors	Conference abstract
A. L. Franks	2012	Multiple associations between a broad spectrum of autoimmune diseases, chronic inflammatory diseases and cancer	Review
H. J. Freeman	1996	Neoplastic disorders in 100 patients with adult celiac disease	wrong estimate
H. J. Freeman	2004	Lymphoproliferative and intestinal malignancies in 214 patients with biopsy-defined celiac disease	wrong estimate
H. J. Freeman	2009	Malignancy in adult celiac disease	wrong estimate
H. J. Freeman	2009	Adult celiac disease and its malignant complications	wrong estimate
A. F. Fundia	2009	[Genetic alterations, genomic instability and cancer in celiac disease]	wrong estimate
M. Gayed	2009	Lupus and cancer	Review
H. C. Gerstein	2010	Does insulin therapy promote, reduce, or have a neutral effect on cancers?	wrong outcome
E. Giovannucci	2010	Diabetes and cancer: A consensus report	wrong study design
C. Gomes	2022	Celiac disease presenting as a jejunal adenocarcinoma with severe iron deficiency anemia	Case report
Y. Gong	2012	ABO blood type, diabetes and risk of gastrointestinal cancer in Northern China	T2D/ wrong exposure
R. Gonzalez	2011	Risk of colorectal neoplasia in patients with celiac disease: A multicentric study	Conference abstract

R. Gonzalez	2013	Risks factors for colorectal neoplasia in patients with celiac disease: A multicentric study	No estimate for colorectal cancer/ wrong estimate
G. Goobie	2016	Autoantibody profiles in patients from the systemic lupus international collaborating clinics (SLICC) cohort with and without malignancy	Conference abstract
G. C. Goobie	2015	Malignancies in systemic lupus erythematosus: a 2015 update	Review
C. Gordon	2019	Cancer and lupus: What are the links and what should we be looking out for?	Conference abstract
V. L. Z. Gordon-Dseagu	2013	Epidemiological evidence of a relationship between type-1 diabetes mellitus and cancer: A review of the existing literature	Review
A. Goto	2020	Diabetes and cancer risk: a Mendelian randomization study	T2D, wrong exposure
S. B. Graversen	2023	The impact of diabetes on cancer detection during the prevalence round of a national screening program for colorectal cancer	No distinction between T1D and T2D, wrong exposure
P. H. Green	2003	Risk of malignancy in patients with celiac disease	wrong estimate
P. H. Green	2004	Small bowel carcinoma and coeliac disease	Letter
P. H. R. Green	2001	Characteristics of adult celiac disease in the USA: results of a national survey	wrong comparison group
K. Grieve	2012	Cancer and disease modifying therapy in multiple sclerosis: An audit of the tayside DMT register	Conference abstract
I. Gromny	2023	Pancreatic Cancer in Celiac Disease Patients-A Systematic Review and Meta-Analysis	Systematic review and meta-analysis
P. A. Groome	2022	Multiple Sclerosis and the Cancer Diagnosis: Diagnostic Route, Cancer Stage, and the Diagnostic Interval in Breast and Colorectal Cancer	Comparison of diagnostic route/ wrong outcome
N. Grytten	2023	Cancer related mortality in multiple sclerosis. A population based cohort study	wrong comparison group
L. Gullo	1999	Diabetes and the risk of pancreatic cancer	Not clear what diabetes type/wrong exposure
L. Gullo	2004	Diabetes and Pancreatic Cancer [2] (multiple letters)	Letter
L. Gullo	1994	Diabetes and the risk of pancreatic cancer	wrong outcome
J. Guo	2022	Relationship between diabetes and risk of gastric cancer: A systematic review and meta-analysis of cohort studies	Review
J. R. Guo	2022	Relationship between diabetes and risk of gastric cancer: A systematic review and <i>meta</i>-analysis of cohort studies	Review
U. C. Gupta	2023	An Evidence-Based Review of Diabetes Care: History, Types, Relationship to Cancer and Heart Disease, Co-Morbid Factors, and Preventive Measures	T2D / wrong exposure
L. A. Habel	2013	Cohort study of insulin glargine and risk of breast, prostate, and colorectal cancer among patients with diabetes	T1D and T2D mixed/ wrong exposure
M. Haddadi	2023	Epidemiology of Celiac Disease in Northern Morocco in 2018- 2021: A Descriptive Cross-Sectional Study	wrong outcome
M. B. Haider	2022	THE SPECTRUM OF MALIGNANT NEOPLASMS IN CELIAC DISEASE PATIENTS - INSIGHT FROM NATIONAL INPATIENT SAMPLE DATABASE	Abstract
Y. Han	2015	Association Between Coeliac Disease and Risk of Any Malignancy and Gastrointestinal Malignancy: A Meta-Analysis	Review
D. Hardenbergh	2022	The Cancer Risk Profile of Systemic Lupus Erythematosus Patients	Review
D. J. Harman	2012	Prevalence of liver histological abnormalities in type 1 diabetes and the long term consequences	Abstract
O. D. Harris	1967	Malignancy in adult coeliac disease and idiopathic steatorrhoea	Celiac disease and Idiopathic Steatorrhoea/ wrong exposure
J. Hebrero San Martín	1986	[Celiac disease and intestinal adenocarcinoma]	not in English
J. Heck	1984	[Malignant lymphoma of the small intestine after long-existent sprue]	not in English
K. Hemminki	2016	Cancer of unknown primary is associated with diabetes	wrong outcome
R. Hennig	2010	Diabetes is associated to malignant pancreatic tumors and serves as prognostic factor for ductal adenocarcinoma	Abstract
E. Hernandez-Garduno	2021	The association between diabetes and cancer in Mexico: Analysis using death certificate databases, 2009-2017	Prevalences
H. Hjalgrim	1997	Cancer and diabetes--a follow-up study of two population-based cohorts of diabetic patients	wrong comparison group
B. Y. Ho	2013	Malignancy in a cohort of oriental patients with systemic lupus erythematosus (SLE)	wrong comparison group

L. Y. Ho	2013	Relationship between individual organ damage and mortality of systemic lupus erythematosus (SLE): A prospective cohort study of 679 patients	wrong comparison group
G. K. Holmes	2002	Coeliac disease and malignancy	Minisymposium/ wrong study design
G. K. Holmes	1989	Malignancy in coeliac disease--effect of a gluten free diet	wrong comparison group
G. K. Holmes	1976	Coeliac disease, gluten-free diet, and malignancy	wrong comparison group
G. K. T. Holmes	2002	Coeliac disease and malignancy	Minisymposium/ wrong study desing
G. K. T. Holmes	2018	Mortality in coeliac disease: a population-based cohort study from a single centre in Southern Derbyshire, UK	wrong comparison group
G. K. T. Holmes	1989	Malignancy in coeliac disease - Effect of a gluten free diet	wrong comparison group
P. D. Home	2009	Combined randomised controlled trial experience of malignancies in studies using insulin glargine	RCT insuline study, wrong study design
K. Hongell	2019	Risk of cancer among Finnish multiple sclerosis patients	Prevalences
C. Hope	2016	Relationship between HbA1c and cancer in people with or without diabetes: a systematic review	Review
P. D. Howdle	2004	Small bowel malignancy in coeliac disease	Letter
P. D. Howdle	2003	Primary small-bowel malignancy in the UK and its association with coeliac disease	wrong outcome
C. Y. Hsu	2017	Cumulative immunosuppressant exposure is associated with diversified cancer risk among 14 832 patients with systemic lupus erythematosus: a nested case-control study	wrong comparison group
Y. C. Huang	2011	Diabetes mellitus negatively impacts survival of patients with colon cancer, particularly in stage II disease	wrong comparison group
S. Hussain	2022	TYPES OF DIABETES, INTERLINKED WITH CANCER, OTHER DISEASES AND COMPLICATIONS WITH OR WITHOUT CANCER	wrong comparison group
M. Ilyas	1995	Non-Hodgkin's lymphoma, coeliac disease, and Epstein-Barr virus: a study of 13 cases of enteropathy-associated T- and B-cell lymphoma	wrong comparison group
M. Ivanova	2023	Malignancies in Patients with Celiac Disease: Diagnostic Challenges and Molecular Advances	Review
V. T. Ivashkinl	2015	[Autoimmune Diseases of Digestive System]	Review
A. Iyengar	2017	Association of diabetes with colorectal cancer treatment and outcomes	Review
M. Janghorbani	2012	Systematic Review and Meta-analysis of Insulin Therapy and Risk of Cancer	Review
J. Ji	2018	Family history of autoimmune diseases and risk of gastric cancer: A national cohort study	Prevalence
S. Jick	2018	Increased risk of comorbidities in patients before as well as after multiple sclerosis (MS) diagnosis: A study in the UK Clinical Practice Research Datalink (CPRD)	Abstract
N. Joharatnam-Hogan	2023	Diabetes and cancer: Optimising glycaemic control	T2D, Review
S. D. Johnston	2000	Small bowel lymphoma in unrecognized coeliac disease: a cause for concern?	Prevalence
E. Jullumstrø	2009	Diabetes mellitus and outcomes of colorectal cancer	Prevalence
N. Kalra	2022	Current updates on the association between celiac disease and cancer, and the effects of the gluten-free diet for modifying the risk (Review)	Review
R. Kath	2000	Malignancies in patients with insulin-treated diabetes mellitus	T1D und T2D/ wrong exposure
V. A. Katzke	2020	Diabetes and cancer Epidemiological associations and biologic mechanisms	not in English
K. Kaukinen	2021	Updates on systemic consequences of coeliac disease	Comment
B. J. Kendall	2009	Undiagnosed coeliac disease and risk of oesophageal squamous cell carcinoma	Abstract
S. Kenwright	1972	CELIAC DISEASE AND SMALL BOWEL CARCINOMA	Review
S. Kenwright	1972	Coeliac disease and small bowel carcinoma	Review
H. Khalili	2012	Is diabetes a risk factor for colorectal cancer?	Editorial
Z. Khan	2018	INCIDENCES OF MALIGNANCY IN CELIAC DISEASE IN US POPULATION: A 13 YEARS NATIONAL INPATIENT SAMPLE ANALYSIS	Abstract
G. Khoudari	2019	The prevalence and epidemiology of pancreatic cancer in the USA: A population-based study	Abstract
S. Kim	2011	Syndecan as a messenger to link diabetes and cancer	Review
E. Kingwell	2009	Cancer risk in an untreated population of multiple sclerosis patients: Preliminary results from the malignancy in multiple sclerosis study	Abstract

E. Kingwell	2011	No evidence of increased cancer risk associated with betainterferon treatment for multiple sclerosis: Preliminary findings from the Malignancy in MS [MaMS] study	wrong comparison group
E. Kingwell	2013	Cancer risk following beta-interferon treatment for multiple sclerosis: Findings from the malignancy in ms [MAMS] study	Abstract
E. Kingwell	2014	Assessment of cancer risk with s-interferon treatment for multiple sclerosis	wrong comparison group
E. Kingwell	2014	Assessment of cancer risk with β-interferon treatment for multiple sclerosis	wrong comparison group
E. Kingwell	2013	The relative risk of cancer death in men and women with multiple sclerosis: Findings from the malignancy in multiple sclerosis (MaMS) study	Abstract
E. Kiss	2010	Malignancies in systemic lupus erythematosus	Review
A. Krishnan	2022	Increased Risk of Pancreatitis, Pancreatic Cancer, and Mortality in Patients With Celiac Disease	Abstract
A. Kuhn	1986	[Subacute cutaneous lupus erythematosus as a paraneoplastic syndrome]	Case Report
F. Kulali	2018	The imaging findings of duodenal adenocarcinoma in patient with celiac disease	Case Report
C. F. Kuo	2019	Temporal relationships between systemic lupus erythematosus and comorbidities	wrong outcome
L. La Mantia	2021	Cancer risk for multiple sclerosis patients treated with azathioprine and disease-modifying therapies: an Italian observational study	wrong comparison group
C. La Vecchia	1997	Diabetes mellitus and colorectal cancer risk	Keine Unterscheidung T1D/T2D/ wrong exposure
C. La Vecchia	1997	Diabetes mellitus and the risk of primary liver cancer	Keine Unterscheidung T1D/T2D/ wrong exposure
C. La Vecchia	1994	A case-control study of diabetes mellitus and cancer risk	Keine Unterscheidung T1D/T2D/ wrong exposure
A. Ladouceur	2019	Malignancies in systemic lupus erythematosus: an update	Review
A. Ladouceur	2020	Cancer and Systemic Lupus Erythematosus	Review
J. Lasa	2018	Colorectal adenoma risk is increased among recently diagnosed adult celiac disease patients	wrong outcome
J. Lasa	2018	Risk of colorectal adenomas in patients with celiac disease: a systematic review and meta-analysis	Review
J. S. Lasa	2018	PREVALENCE OF COLORECTAL ADENOMAS AMONG RECENTLY-DIAGNOSED ADULT CELIAC DISEASE PATIENTS	Abstract
P. Laurikka	2022	Review article: Systemic consequences of coeliac disease	Review
B. Lebwohl	2010	Risk of colorectal adenomas in patients with coeliac disease	Wrong outcome
Y. N. Lee	2020	Diabetes: an Overview for Clinical Oncologists	Review
I. C. Lega	2020	Review: Diabetes, Obesity, and Cancer-Pathophysiology and Clinical Implications	Review
C. E. Leijonmarck	1985	[Celiac disease increases the risk of malignancy]	Case Report
N. Leopa	2021	Colorectal cancer in patients with diabetes mellitus	T2D/ wrong outcome
N. R. Lewis	2010	Risk of morbidity in contemporary celiac disease	Review
X. Li	2018	Relationship between diabetes mellitus and the risk of pancreatic cancer	Review
S. Ling	2021	Risk of cancer incidence and mortality associated with diabetes: A systematic review with trend analysis of 203 cohorts	Review
Q. Liu	2023	Association between multiple sclerosis and cancer risk: An extensive review/meta and Mendelian randomization analyses	Review
X. L. Liu	2018	Association between insulin therapy and risk of liver cancer among diabetics: a meta-analysis of epidemiological studies	Review
R. Ljung	2011	Insulin glargine use and short-term incidence of malignancies - A three-year population-based observation	T1D und T2D/ wrong exoposre
C. G. Loftus	2002	Cancer risk in celiac disease	Editorial
R. F. Logan	1989	Mortality in celiac disease	mortality/ wrong outcome
J. F. Ludvigsson	2012	Mortality and malignancy in celiac disease	Review
S. Luo	2016	Diabetes mellitus increases the risk of colorectal neoplasia: An updated meta-analysis	Metaanalyse

S. F. Luo; C. F. Kuo	2017	Comorbidities in patients with systemic lupus erythematosus prior to and following diagnosis: Case-control study	Abstract
R. I. L. Machado	2014	Incidence of neoplasms in the most prevalent autoimmune rheumatic diseases: A systematic review	Review
J. T. Magruder	2011	Diabetes and pancreatic cancer: chicken or egg?	Review
M. Magyari	2020	Comorbidity in Multiple Sclerosis	Review
P. Maisonneuve	2010	Epidemiology of pancreatic cancer: An update	Review
S. Mao	2016	Systemic lupus erythematosus and malignancies risk	Review
I. Marafini	2020	Association Between Celiac Disease and Cancer	Review
G. Maric	2022	Occurrence of malignant diseases in patients with multiple sclerosis: population-based MS Registry data	wrong outcome
S. Marimuthu	2011	Diabetes mellitus and gastric carcinoma: Is there an association?	Review
A. Mariottini	2022	Incidence of malignant neoplasms and mortality in people affected by multiple sclerosis in the epoch of disease-modifying treatments: A population-based study on Tuscan residents	wrong outcome
J. Marley	2021	Associations between long-term conditions and upper gastrointestinal cancer incidence: A prospective population-based cohort of UK Biobank participants	Excluded at data extraction
M. Marovt	2017	Subacute cutaneous lupus erythematosus - paraneoplastic to gastric adenocarcinoma?	Letter
R. A. Marrie	2021	Colorectal Cancer Survival in Multiple Sclerosis: A Matched Cohort Study	wrong outcome
R. A. Marrie	2015	A systematic review of the incidence and prevalence of cancer in multiple sclerosis	Review
S. Marsch	1990	[Risk of malignancies in celiac disease--a retrospective study]	wrong comparison group
S. C. U. Marsch	1990	Adenocarcinoma of the small intestine associated with celiac disease	wrong outcome
G. Mazzacca	1993	Diet, coeliac disease and gastrointestinal neoplasm	Review
G. McNeill	2012	The presentation of coeliac disease as a disease-related malignancy	Comment
M. Michael	2003	Recognizing and managing celiac disease in primary care	Review
S. Mizuno	2013	Risk factors and early signs of pancreatic cancer in diabetes: Screening strategy based on diabetes onset age	T1D und T2D/ wrong exposure
A. Mohammed	2021	Small bowel adenocarcinoma: A nationwide population-based study	Abstract
A. Mohammed	2023	Small Bowel Adenocarcinoma: a Nationwide Population-Based Study	wrong estimate
I. Moreira	2013	Multiple sclerosis and extra central nervous system malignancies	Abstract
A. Morimoto	2013	Cause-specific mortality trends in a nationwide population-based cohort of childhood-onset type 1 diabetes in Japan during 35 years of follow-up: The DERI Mortality Study	mortality in T1D/ wrong outcome
F. Moroni	2016	Small bowel adenocarcinoma: Single centre experience over 6 years	Abstract
R. Mukherjee	2012	Gastrointestinal cancer in celiac disease: the first days are the hardest days, don't you worry anymore?""	Editorial
Nct	2023	The Impact of Metformin Administration on the Clinical Outcome of Stage IV Colon Cancer	wrong estimate
R. Nelson	1997	Diabetes mellitus and risk of large bowel cancer	Letter
J. Ni	2014	Lung, liver, prostate, bladder malignancies risk in systemic lupus erythematosus: evidence from a meta-analysis	Review
A. Nicolucci	2010	Epidemiological aspects of neoplasms in diabetes	Review
O. H. Nielsen	1985	Non-tropical sprue. Malignant diseases and mortality rate	wrong estimate
C. J. O'Brien	1983	Coeliac disease, adenocarcinoma of jejunum and in situ squamous carcinoma of oesophagus	Case report
T. M. O'Connor	1999	Type 1 diabetes mellitus, coeliac disease, and lymphoma: a report of four cases	Case report
K. Okumura	2022	Re: Association between intestinal neoplasms and celiac disease - beyond celiac disease and more	Letter
O. Olen	2011	Coeliac disease characteristics, compliance to a gluten free diet and risk of lymphoma by subtype	wrong comparison group
S. Onwuzo	2023	Increased Risk of Colorectal Cancer in Patients With Celiac Disease: A Population-Based Study	wrong comparison group
B. Packova	2022	Malignant complications of celiac disease: a case series and review of the literature	Case Report
B. Packová	2020	Small bowel adenocarcinoma diagnosed by video capsule endoscopy in a patient with celiac disease: a case report and review of literature	Case Report

J. Palo	1977	Malignant diseases among patients with multiple sclerosis	wrong outcome
S. Y. Pan	2011	Epidemiology of cancer of the small intestine	Editorial
A. Pandey	2011	Diabetes mellitus and the risk of cancer	Review
F. Pelizzaro	2021	The Risk of Malignancies in Celiac Disease-A Literature Review	Review
P. J. Pemberton	1972	Adult celiac disease, reticulosis and carcinoma	Case Report
L. Pereyra	2013	Risk of colorectal neoplasia in patients with celiac disease: a multicenter study	wrong outcome
M. C. Perrin	2007	Gestational diabetes as a risk factor for pancreatic cancer: a prospective cohort study	wrong exposure
R. Persson	2019	Increased risk of comorbidities in patients before and after multiple sclerosis diagnosis and initiation of treatment: A study using the US department of defense database	wrong outcome
U. Peters	2003	Causes of death in patients with celiac disease in a population-based Swedish cohort	Mortality/ wrong outcome
J. L. Petrick	2017	Risk factors for intrahepatic and extrahepatic cholangiocarcinoma in the United States: A population-based study in SEER-Medicare	wrong otucome
D. D. Potter	2004	The role of defective mismatch repair in small bowel adenocarcinoma in celiac disease	wrong outcome
O. B. Poyrazoglu	2021	Celiac disease is increased in esophageal squamous cell Carcinoma	wrong estimate
V. E. Pricolo	1998	Gastrointestinal malignancies in patients with celiac sprue	wrong estimate
C. Prince	2021	Integrative multiomics analysis highlights immune-cell regulatory mechanisms and shared genetic architecture for 14 immune-associated diseases and cancer outcomes	MR study/ wrong study design
D. T. Purtilo	1983	Coeliac disease and malignancy	Abstract
P. Ragonese	2017	Association between multiple sclerosis, cancer risk, and immunosuppressant treatment: A cohort study	wrong outcome
S. D. Rampertab	2003	Risk of duodenal adenoma in celiac disease	wrong outcome
S. D. Rampertab	2003	Small bowel neoplasia in coeliac disease	Case Report
R. Ramsey-Goldman	2016	Standardised incidence ratios (SIRs) for cancer after renal transplant in systemic lupus erythematosus (SLE) and non-SLE recipients	wrong exposure
R. Ramsey-Goldman	2013	Standardized incidence ratios for cancer after renal transplant in systemic lupus erythematosus and non-systemic lupus erythematosus recipients	Abstract
M. Rewers	2005	Epidemiology of celiac disease: What are the prevalence, incidence, and progression of celiac disease?	Review
U. Ribeiro, Jr.	1996	Risk factors for squamous cell carcinoma of the oesophagus	Review
A. M. Roch	2016	Intraductal papillary mucinous neoplasm of the pancreas, one manifestation of a more systemic disease?	wrong outcome
N. Rooney	2004	Gastrointestinal lymphoma	wrong estimate
W. Rösch	1978	[High risk groups for gastrointestinal carcinoma]	not in English
M. Rostami Nejad	2013	Geographic trends and risk of gastrointestinal cancer among patients with celiac disease in Europe and Asian-Pacific region	Review
M. Salmeron	1984	[Malignant lymphoma and celiac disease in adults]	Review, French
A. Saraiva	2023	CANCER PREVALENCE IN SYSTEMIC LUPUS ERYTHEMATOSUS: A PORTUGUESE COHORT STUDY WITH 15 YEARS OF FOLLOW-UP	Abstract
L. Scappaticcio	2017	Insights into the relationships between diabetes, prediabetes, and cancer	Review
R. Schiel	2006	Diabetes, insulin, and risk of cancer	Review
R. Schiel	2005	Risk of malignancies in patients with insulin-treated diabetes mellitus: results of a population-based trial with 10-year follow-up (JEVIN)	wrong estimate
M. Schumann	2015	Celiac disease and intestinal lymphomas	Editorial
J. J. Schweizer	2001	Cancer in children with celiac disease: a survey of the European Society of Paediatric Gastroenterology, Hepatology and Nutrition	Short Communication
M. Seabra	2017	Cancer and multiple sclerosis: Case series from a reference centre	
W. S. Selby	1979	Malignancy in a 19-year experience of adult celiac disease	wrong estimate
M. S. Seo	2019	Risk of pancreatic cancer in patients with systemic lupus erythematosus: a meta-analysis	Review
I. Shah	2021	ID: 3525490 PROSPECTIVE ASSESSMENT OF NEW ONSET DIABETES MELLITUS AND PRE DIABETES MELLITUS IN PATIENTS UNDERGOING PANCREATIC CANCER SCREENING	Abstract
M. Shahrivar	2023	ASSOCIATION BETWEEN DIABETES, METFORMIN USE, AND PROGNOSIS IN STAGE I-III COLORECTAL CANCER PATIENTS: A NATIONWIDE REGISTER-BASED STUDY	wrong outcome

L. R. Sharafutdinova	2014	Cancer diseases in patients with multiple sclerosis in the Bashkortostan Republic	text not in English
S. Sharma	2022	Predicting Pancreatic Cancer in the UK Biobank Cohort Using Polygenic Risk Scores and Diabetes Mellitus	Poligenic risk score and risk of pancreas cancer in Diabetes/non-Diabetes/ wrong exposure
S. Shenoy	2016	Genetic risks and familial associations of small bowel carcinoma	Review
C. Shieh	2019	Exogenous insulin and risk of colorectal adenomas in type I diabetes	Abstract
K. Shikata	2013	Diabetes mellitus and cancer risk: Review of the epidemiological evidence	Review
P. Shringi	2016	Occurrence of malignancy in SLE: A rare association between SLE and adenocarcinoma of colon	Case Report
M. Silano	2005	Risk of gastrointestinal non-Hodgkin's lymphoma at diagnosis of celiac disease	Letter
M. Silano	2005	Small bowel malignancy at diagnosis of coeliac disease	Letter
M. Silano	2007	Delayed diagnosis of coeliac disease increases cancer risk	Prevalence
D. Simon	2010	Diabetes mellitus, hyperglycaemia and cancer	Review
K. E. Smedby	2005	Malignant lymphomas in coeliac disease: Evidance of increased risks for lymphoma types other than enteropathy-type T cell lymphoma	wrong outcome
M. F. Sona	2018	Type 1 diabetes mellitus and risk of cancer: a meta-analysis of observational studies	Metaanalyse
C. S. Sondergaard	2023	Use of Antihyperglycemic Drugs and Risk of Cancer in Patients with Diabetes	Review
L. Song	2018	The risks of cancer development in systemic lupus erythematosus (SLE) patients: a systematic review and meta-analysis	Review
M. Song	2019	Abstract 5051: Associations between autoimmune conditions and gastric cancer risk among elderly us adults	Abstract
M. Song	2019	Autoimmune Diseases and Gastric Cancer Risk: A Systematic Review and Meta-Analysis	Review
R. J. Stevens	2007	Pancreatic cancer in type 1 and young-onset diabetes: systematic review and meta-analysis	Review
S. Suh	2019	Diabetes and Cancer: Cancer Should Be Screened in Routine Diabetes Assessment	Review
S. M. Sultan	2000	Is there an association of malignancy with systemic lupus erythematosus? An analysis of 276 patients under long-term review	wrong outcome
L. Sun	2012	Diabetes mellitus is an independent risk factor for colorectal cancer	Review
S. Svacina	2004	Gastrointestinal tract cancer and diabetes mellitus	Review, Czech
C. M. Swinson	1983	Coeliac disease and malignancy	wrong exposure
L. Szablewski	2014	Diabetes mellitus: influences on cancer risk	Review
E. M. Tan	2003	Relative paradigms between autoantibodies in lupus and autoantibodies in cancer	Review
X. Tang	2012	Insulin glargine and cancer risk in patients with diabetes: a meta-analysis	Review
T. Tarr	2007	Occurrence of malignancies in Hungarian patients with systemic lupus erythematosus: results from a single center	case numbers
T. Tarr	2006	[The occurrence of malignancies in a Hungarian lupus population]	Article in Hungarian, not in English
B. Tessier-Cloutier	2014	Systemic lupus erythematosus and malignancies: a review article	Review
C. Tomba	2014	Enteroscopy for the early detection of small bowel tumours in at-risk celiac patients	wrong estimate
D. Tomic	2022	The burden and risks of emerging complications of diabetes mellitus	Review
J. Trna	2010	The relationship between diabetes mellitus and pancreatic cancer and the implications for screening	Review
C. H. Tseng	2011	Diabetes conveys a higher risk of gastric cancer mortality despite an age-standardised decreasing trend in the general population in Taiwan	Mortality/ wrong Outcome
C. H. Tseng	2013	Diabetes, insulin use, and gastric cancer: a population-based analysis of the Taiwanese	T1D und T2D, wrong exposure
C. H. Tseng	2021	The Relationship between Diabetes Mellitus and Gastric Cancer and the Potential Benefits of Metformin: An Extensive Review of the Literature	Review
C. H. Tung	2013	Hepatic complication in autoimmune rheumatic diseases: A nationwide cohort study in Taiwan	Abstract
E. Van Cutsem	1991	Epidemiology and clinical aspects of esophageal cancer	Review

T. van Gils	2018	Risks for lymphoma and gastrointestinal carcinoma in patients with newly diagnosed adult-onset celiac disease: Consequences for follow-up: Celiac disease, lymphoma and GI carcinoma	Prevalences
L. Van Overbeke	2005	What is the role of celiac disease in enteropathy-type intestinal lymphoma? A retrospective study of nine cases	wrong outcome
A. Vanoli	2015	Celiac disease-associated small bowel adenocarcinomas: Clinico-pathologic, phenotypic and molecular study of 11 cases	Abstract
L. Váróczy	2002	Malignant lymphoma-associated autoimmune diseases--a descriptive epidemiological study	wrong estimate
V. Verkarre	2004	Gluten-free diet, chromosomal abnormalities, and cancer risk in coeliac disease	Editorial
P. Vigneri	2009	Diabetes and cancer	Review
H. L. Wang	2018	Malignancy as a comorbidity in rheumatic diseases: a retrospective hospital-based study	wrong outcome
M. Wang	2020	Diabetes and cancer: Epidemiological and biological links	Review
M. Wang	2021	Association between intestinal neoplasms and celiac disease: A review	Review
Y. Wang	2022	Gluten Intake and Risk of Digestive System Cancers in 3 Large Prospective Cohort Studies	wrong exposure
Q. Wei	2023	Association between Gastric Cancer and 12 Autoimmune Diseases: A Mendelian Randomization Study	MR Studie/ wrong study design
P. Wellhoner	2011	Endocrine disorders and the gastrointestinal tract : What consequences are possible?	text not English
M. Wender	1987	[Comparative epidemiologic studies of multiple sclerosis and cancer of the large intestine in several provinces of western Poland]	Text not in English
J. West	2009	Celiac Disease and Its Complications: A Time Traveller's Perspective	Editorial
J. West	2004	Malignancy and mortality in people with coeliac disease: population based cohort study	Excluded at data extraction
J. West	2004	Malignancy and mortality in people with coeliac disease: population based cohort study	Duplicate
P. J. Westlake	1989	Adenocarcinoma of the stomach in celiac disease	Case Report
D. C. Whitcomb	2014	Diabetes and cancer: The problem of reverse causality and missing links	Abstract
S. Widgren	1998	Adenocarcinoma of the small bowel, coeliac disease, and lymphocytic gastritis	Letter
S. H. Wild	2011	Diabetes, treatments for diabetes and their effect on cancer incidence and mortality: Attempts to disentangle the web of associations	Commentary
J. Wise	2016	Type 1 diabetes is associated with increased risk of several cancers	Abstract
D. H. Wright	1995	The major complications of coeliac disease	Review
J. W. Wu	2016	Effect of long-acting insulin analogs on the risk of cancer: A systematic review of observational studies	Review
L. Wu	2013	Diabetes mellitus and the occurrence of colorectal cancer: an updated meta-analysis of cohort studies	Metaanalyse
D. Yadav	2013	The epidemiology of pancreatitis and pancreatic cancer	Review
Q. Yin	2023	Mendelian Randomization Analyses of Chronic Immune-Mediated Diseases, Circulating Inflammatory Biomarkers, and Cytokines in Relation to Liver Cancer	MR Studie
H. Yu	2019	Clinical characteristics of systemic lupus erythematosus with malignant tumors	Abstract
F. Yuan	2019	GENETIC SUSCEPTIBILITY TO CHRONIC INFLAMMATORY INTESTINAL DISEASES AND PANCREATIC DUCTUAL ADENOCARCINOMA: A PATHWAY ANALYSIS OF GENOME-WIDE ASSOCIATION STUDIES	Abstract
F. Yuan	2020	Genome-Wide Association Study Data Reveal Genetic Susceptibility to Chronic Inflammatory Intestinal Diseases and Pancreatic Ductal Adenocarcinoma Risk	wrong study design
H. Yuhara	2011	Is diabetes mellitus an independent risk factor for colon cancer and rectal cancer?	Metaanalyse
N. Zádori	2021	Six Autoimmune Disorders Are Associated With Increased Incidence of Gastric Cancer: A Systematic Review and Meta-Analysis of Half a Million Patients	Review
C. Zarnavalou	2016	Autoimmune diseases and correlation with malignancies	Review
M. Zhang	2022	Association Between Systemic Lupus Erythematosus and Cancer Morbidity and Mortality: Findings From Cohort Studies	Review
B. Zhu	2022	The Relationship Between Diabetes Mellitus and Cancers and Its Underlying Mechanisms	Review
L. Zhu	2020	Revealing the Interactions Between Diabetes, Diabetes-Related Diseases, and Cancers Based on the Network Connectivity of Their Related Genes	Wrong estimate

L. Chen	2024	Exploring potential causal associations between autoimmune diseases and colorectal cancer using bidirectional Mendelian randomization	MR, wrong study design
M. A. Getu	2024	A two-sample Mendelian randomization study of type 1 diabetes and the risk of 22 site-specific cancers	MR, wrong study design
Z. Liu	2024	Association between multiple sclerosis and cancer risk: A two-sample Mendelian randomization study	MR, wrong study design
Y. B. Long	2024	The Genetic Causal Effect of Autoimmune Diseases on pan-Cancers: Evidence from Mendelian Randomization	MR, wrong study design
M. Pliszka	2024	Associations between Diabetes Mellitus and Selected Cancers	Review
J. L. Zhao	2024	Casual effects of type 1 diabetes mellitus on site-specific digestive cancers: a Mendelian randomisation analysis	MR, wrong study design
H. A. *Luigina Bonelli	2003	Exocrine Pancreatic Cancer, Cigarette Smoking, and Diabetes Mellitus: A Case-Control Study in Northern Italy	t2d, wrong exposure
BARBARA A. O'MARA'	1985	DIABETES MELLITUS AND CANCER RISK: A MULTISITE CASE-CONTROL STUDY	t2d age > 30, wrong exposure
Q. Ben	2011	Diabetes mellitus and risk of pancreatic cancer: A meta-analysis of cohort studies	t2d, wrong exposure
H. B. Bueno de Mesquita	1992	Aspects of medical history and exocrine carcinoma of the pancreas: a population-based case-control study in The Netherlands	no strict distinction between t1d and t2d, wrong exposure
B. Carstensen	2012	Cancer occurrence in Danish diabetic patients: duration and insulin effects	mainly t2d, wrong exposure
J. A. Davila	2005	Diabetes increases the risk of hepatocellular carcinoma in the United States: a population based case control study	exposure not specified
M. S. DT Silverman	1999	Diabetes mellitus, other medical conditions and familial history of cancer as risk factors for pancreatic cancer	only adult onset diabetes, exposure not specified
A. Flood	2010	Diabetes and risk of incident colorectal cancer in a prospective cohort of women	t2d, wrong exposure
M. M. Hassan	2010	Association of diabetes duration and diabetes treatment with the risk of hepatocellular carcinoma	t2d, wrong exposure
J. He	2010	The association of diabetes with colorectal cancer risk: the Multiethnic Cohort	t2d, wrong exposure
A. Hidalgo-Conde	2013	Incidence of cancer in a cohort of Spanish patients with systemic lupus erythematosus	wrong outcome
D. A. G. Julie C. Will	1998	Colorectal Cancer: Another Complication of Diabetes Mellitus?	t2d, wrong exposure
S. N. Kyle Steenland	1995	Cancer Incidence Follow-up in the Data: and National Diabetes, Physical Health and Cholesterol, Activity Nutrition	exposure not specified
S. M. LA Anderson	2007	Malignancy and mortality in a population-based cohort of patients with coeliac disease or 'gluten sensitivity'	review
D. Li	2011	Diabetes and risk of pancreatic cancer: a pooled analysis of three large case-control studies	t2d, wrong exposure
K. F. Liao	2012	Diabetes mellitus correlates with increased risk of pancreatic cancer: a population-based cohort study in Taiwan	exposure not specified
L. R. W. Lolc Le Marchand	1997	Associations of Sedentary with the Risk of Colorectal Lifestyle, Cancer' Obesity, Smoking, Alcohol Use, and Diabetes	exposure not specified
G. G. Louise Wideroff	1997	Cancer Incidence in a Population-Based Cohort of Patients Hospitalized With Diabetes Mellitus in Denmark	exposure not specified
G. G. Louise Wideroff	1994	Cancer Incidence in a Population-Based Cohort of Patients Hospitalized With Diabetes Mellitus in Denmark	exposure not specified
P. Maisonneuve	2010	Past medical history and pancreatic cancer risk: Results from a multicenter case-control study	exposure not specified
M. Manami Inoue	2006	Diabetes Mellitus and the Risk of Cancer Results From a Large-Scale Population-Based Cohort Study in Japan	exposure not specified
MARK RAGOZZINO*	1982	SUBSEQUENT CANCER RISK IN THE INCIDENCE COHORT OF ROCHESTER, MINNESOTA, RESIDENTS WITH DIABETES MELLITUS	exposure not specified
A. B. O Nived1*	2001	Malignancies during follow-up in an epidemiologically de®ned systemic lupus erythematosus inception cohort in southern Sweden	wrong estimate
H. K. Pagona Lagiou	2000	Role of Diabetes Mellitus in the Etiology of Hepatocellular Carcinoma	exposure not specified
J. Pearson-Stuttard	2018	Worldwide burden of cancer attributable to diabetes and high body-mass index: a comparative risk assessment	exposure not specified
M. C. Rousseau	2006	Diabetes mellitus and cancer risk in a population-based case-control study among men from Montreal, Canada	t2d, wrong exposure
A. Seow	2006	Diabetes mellitus and risk of colorectal cancer in the Singapore Chinese Health Study	t2d, wrong exposure
P. Sun Ha Jee	2005	Fasting Serum Glucose Level and Cancer Risk in Korean Men and Women	exposure not specified
L. C. Vinikoor	2009	The association between diabetes, insulin use, and colorectal cancer among Whites and African Americans	t2d, wrong exposure

Table 4: Results from meta-regression analyses

Association	Predictor category	Estimate	cil	ciu	P	R^2
Celiac disease_Pancreas	type_estOR	-0,50	-0,90	-0,09	0,023	100
Celiac disease_Pancreas	type_estRR	0,20	-0,66	1,07	0,596	100
Celiac disease_Pancreas	type_estSIR	-0,59	-1,02	-0,15	0,016	100
Celiac disease_Colorectal	otcmColorectal	-0,25	-0,46	-0,05	0,020	100
Celiac disease_Colorectal	otcmRectum	-0,46	-0,69	-0,24	0,001	100
Celiac disease_Colorectal	time_window0	0,25	0,05	0,45	0,018	100
Lupus_Colorectal	otcmColorectal	-0,56	-0,75	-0,36	0,000	100
Lupus_Colorectal	otcmRectum	-0,44	-0,87	-0,01	0,045	100
Lupus_Pancreas	regionEurope	-0,33	-0,46	-0,19	0,000	100
Lupus_Pancreas	regionNorthAmerica/Australia	-0,59	-0,77	-0,42	0,000	100
Lupus_Pancreas	regionTransethnic	-0,80	-1,55	-0,04	0,041	100
Lupus_Liver	type_estSIR	0,57	0,06	1,08	0,033	100
Lupus_Liver	suff_cases10	-0,56	-1,06	-0,06	0,033	100
Type I diabetes_Colorectal	otcmColorectal	-0,13	-0,28	0,02	0,083	97
Type I diabetes_Colorectal	otcmRectum	-0,19	-0,37	0,00	0,048	97
Type I diabetes_Colorectal	regionEurope	-0,44	-0,77	-0,10	0,014	97
Type I diabetes_Colorectal	regionNorthAmerica/Australia	-0,22	-0,57	0,13	0,194	97
Type I diabetes_Colorectal	regionTransethnic	-0,35	-0,63	-0,07	0,017	97
Type I diabetes_Colorectal	time_window0	-0,40	-0,72	-0,09	0,015	97
Type I diabetes_Pancreas	regionEurope	-1,28	-2,53	-0,03	0,045	100
Type I diabetes_Pancreas	regionNorthAmerica/Australia	-0,31	-1,28	0,66	0,471	100
Type I diabetes_Pancreas	regionTransethnic	-1,85	-3,23	-0,46	0,016	100
Type I diabetes_Pancreas	type_estOR	-1,78	-2,97	-0,59	0,010	100
Type I diabetes_Pancreas	type_estSIR	-0,97	-2,05	0,10	0,069	100

The time window indicates whether a study left a period of at least 1 year between the diagnosis of exposure and outcome. Suff_cases10 indicates whether at least 10 cancer cases occurred within the respective autoimmune disease.

Table 5: Heterogeneity statistics and tests for funnel plot asymmetry for models with at least 10 studies (before outlier-removal)

association	model	k	outlier	I^2	tau^2	P_Q	P_{Egger}	P_{Begg}
Celiac disease on Colorectal	unadjusted	17		65,1	0,042	0,000	0,379	0,805
Celiac disease on Colorectal	outl-adjusted	15	2	51,8	0,011	0,010	0,284	0,903
Celiac disease on Pancreas	unadjusted	11		69,2	0,074	0,000	0,922	0,243
Celiac disease on Pancreas	outl-adjusted	10	1	49,2	0,031	0,039	0,815	0,128
Lupus on Colorectal	unadjusted	18		70,0	0,032	0,000	0,025	0,570
Lupus on Colorectal	outl-adjusted	16	2	8,1	0,003	0,361	0,460	0,242
Lupus on Hepatobiliary	unadjusted	15		35,0	0,07	0,089	0,166	0,125
Lupus on Hepatobiliary	outl-adjusted	14	1	0,0	0,000	0,452	0,338	0,171
Lupus on Liver	unadjusted	11		48,0	0,124	0,037	0,133	0,139
Lupus on Liver	outl-adjusted	10	1	20,8	0,027	0,251	0,292	0,180
Lupus on Pancreas	unadjusted	14		81,3	0,049	0,000	0,805	0,171
Lupus on Pancreas	outl-adjusted	13	1	70,4	0,032	0,000	0,724	0,272
MS on Colorectal	unadjusted	12		44,3	0,046	0,043	0,685	0,464
MS on Colorectal	outl-adjusted	11	1	16,7	0,005	0,280	0,126	0,075
T1D on Colorectal	unadjusted	22		64,4	0,037	0,000	0,321	0,937
T1D on Colorectal	outl-adjusted	19	3	41,1	0,012	0,029	0,679	0,910
T1D on Hepatobiliary	unadjusted	18		95,4	0,41	0,000	0,041	0,649
T1D on Hepatobiliary	outl-adjusted	14	4	61,7	0,058	0,001	0,585	0,697
T1D on Liver	unadjusted	13		96,4	0,473	0,000	0,130	0,870
T1D on Liver	outl-adjusted	8	5	60,7	0,031	0,009	0,927	0,835
T1D on Pancreas	unadjusted	12		91,3	0,246	0,000	0,042	0,272
T1D on Pancreas	outl-adjusted	9	3	71,1	0,103	0,000	0,607	0,929
T1D on Stomach	unadjusted	12		42,2	0,023	0,054	0,681	0,222
T1D on Stomach	outl-adjusted	12	0	42,2	0,023	0,054	0,681	0,222

Abbreviations: T1D, type I diabetes, k, number of included studies.

Table 6: Bias-corrected estimates adjusted for all identified sources of bias

Exposure	Outcome	Studies	RR	cil	ciu	P_fdr
Celiac disease	Pancreas	10	1,40	1,15	1,69	0,000
Lupus	Pancreas	13	1,62	1,36	1,93	0,000
Lupus	Liver	10	1,96	1,58	2,42	0,000
Lupus	Stomach	8	1,17	0,96	1,44	0,180
Lupus	Colorectal	16	1,02	0,90	1,15	0,802
Type I diabetes	Colorectal	20	1,16	1,07	1,26	0,000
Type I diabetes	Liver	9	1,84	1,54	2,20	0,000
Type I diabetes	Pancreas	10	1,50	1,14	1,98	0,000
Lupus	Hepatobiliary	14	1,83	1,77	1,90	0,000
Celiac disease	Colorectal	15	1,05	0,93	1,18	0,499
Type I diabetes	Hepatobiliary	15	1,70	1,42	2,03	0,000
Celiac disease	Esophagus	8	1,86	1,42	2,42	0,000
Celiac disease	Stomach	7	1,08	0,88	1,33	0,529
Celiac disease	Small intestine	7	4,19	2,71	6,50	0,000
Celiac disease	Colon	7	1,31	1,09	1,57	0,000
Celiac disease	Rectum	6	0,83	0,65	1,06	0,180
Celiac disease	Liver	7	1,68	1,25	2,26	0,000
Type I diabetes	Esophagus	8	1,49	1,21	1,85	0,000
Type I diabetes	Stomach	13	1,42	1,23	1,64	0,000
Type I diabetes	Colon	8	1,37	1,17	1,60	0,000
Type I diabetes	Rectum	7	1,24	0,98	1,56	0,101
MS	Liver	5	0,75	0,39	1,43	0,456
Lupus	Esophagus	7	1,66	1,46	1,89	0,000
MS	Stomach	6	0,75	0,46	1,21	0,320
MS	Colon	4	0,97	0,86	1,10	0,676
MS	Rectum	4	0,81	0,69	0,96	0,034
MS	Pancreas	6	0,77	0,66	0,90	0,000
Lupus	Colon	6	1,41	1,13	1,75	0,000
Type I diabetes	Gallbladder	5	1,59	1,05	2,41	0,047
Lupus	Rectum	3	0,93	0,66	1,32	0,741
MS	Esophagus	5	0,59	0,40	0,86	0,019
MS	Small intestine	2	1,63	1,00	2,66	0,078
Celiac disease	Hepatobiliary	9	1,61	1,29	2,02	0,000
Type I diabetes	Small intestine	2	1,12	0,42	2,94	0,820
MS	Hepatobiliary	5	0,75	0,39	1,43	0,456
MS	Colorectal	12	0,88	0,81	0,96	0,000

References

References 1: Studies considered in this systematic review and in meta-analyses

1. Askling J, Linet M, Gridley G, Halstensen TS, Ekström K, Ekbom A. Cancer incidence in a population-based cohort of individuals hospitalized with celiac disease or dermatitis herpetiformis. Gastroenterology. 2002;123(5):1428-35.
2. Bernatsky S, Ramsey-Goldman R, Labrecque J, Joseph L, Boivin JF, Petri M, et al. Cancer risk in systemic lupus: an updated international multi-centre cohort study. J Autoimmun. 2013;42:130-5.
3. Buttmann M, Seuffert L, Mäder U, Toyka KV. Malignancies after mitoxantrone for multiple sclerosis: A retrospective cohort study. Neurology. 2016;86(23):2203-7.
4. Carstensen B, Read SH, Friis S, Sund R, Keskimäki I, Svensson AM, et al. Cancer incidence in persons with type 1 diabetes: a five-country study of 9,000 cancers in type 1 diabetic individuals. Diabetologia. 2016;59(5):980-8.
5. Castro FA, Liu X, Försti A, Ji J, Sundquist J, Sundquist K, et al. Increased risk of hepatobiliary cancers after hospitalization for autoimmune disease. Clin Gastroenterol Hepatol. 2014;12(6):1038-45.e7.
6. Chen YJ, Chang YT, Wang CB, Wu CY. Malignancy in systemic lupus erythematosus: A nationwide cohort study in Taiwan. American Journal of Medicine. 2010;123(12):1150.e1-.e6.
7. Dey D, Kenu E, Isenberg DA. Cancer complicating systemic lupus erythematosus--a dichotomy emerging from a nested case-control study. Lupus. 2013;22(9):919-27.
8. Elfström P, Granath F, Ye W, Ludvigsson JF. Low risk of gastrointestinal cancer among patients with celiac disease, inflammation, or latent celiac disease. Clin Gastroenterol Hepatol. 2012;10(1):30-6.
9. Emilsson L, Semrad C, Lebwohl B, Green PHR, Ludvigsson JF. Risk of Small Bowel Adenocarcinoma, Adenomas, and Carcinoids in a Nationwide Cohort of Individuals With Celiac Disease. Gastroenterology. 2020;159(5):1686-94.e2.
10. Fois AF, Wotton CJ, Yeates D, Turner MR, Goldacre MJ. Cancer in patients with motor neuron disease, multiple sclerosis and Parkinson's disease: record linkage studies. J Neurol Neurosurg Psychiatry. 2010;81(2):215-21.
11. Goldacre MJ, Wotton CJ, Yeates D, Seagroatt V, Jewell D. Cancer in patients with ulcerative colitis, Crohn's disease and coeliac disease: record linkage study. Eur J Gastroenterol Hepatol. 2008;20(4):297-304.
12. Gomez-Rubio P, Piñero J, Molina-Montes E, Gutiérrez-Sacristán A, Marquez M, Rava M, et al. Pancreatic cancer and autoimmune diseases: An association sustained by computational and epidemiological case-control approaches. INTERNATIONAL JOURNAL OF CANCER. 2019;144(7):1540-9.
13. Han JY, Kim H, Jung SY, Jang EJ, Cho SK, Sung YK. Increased risk of malignancy in patients with systemic lupus erythematosus: population-based cohort study in Korea. Arthritis Res Ther. 2021;23(1):270.
14. Harding JL, Shaw JE, Peeters A, Cartensen B, Magliano DJ. Cancer risk among people with type 1 and type 2 diabetes: disentangling true associations, detection bias, and reverse causation. Diabetes Care. 2015;38(2):264-70.
15. He MM, Lo CH, Wang K, Polychronidis G, Wang L, Zhong R, et al. Immune-Mediated Diseases Associated With Cancer Risks. JAMA Oncol. 2022;8(2):209-19.
16. Hemminki K, Liu X, Ji J, Sundquist J, Sundquist K. Autoimmune disease and subsequent digestive tract cancer by histology. Annals of Oncology. 2012;23(4):927-33.
17. Hemminki K, Sundquist K, Sundquist J, Forsti A, Liska V, Hemminki A, et al. Autoimmune diseases as comorbidities for liver, gallbladder, and biliary duct cancers in Sweden. Cancer. 2023;129(8):1227-36.
18. Hsu PC, Lin WH, Kuo TH, Lee HM, Kuo C, Li CY. A Population-Based Cohort Study of All-Cause and Site-Specific Cancer Incidence Among Patients With Type 1 Diabetes Mellitus in Taiwan. J Epidemiol. 2015;25(9):567-73.
19. Ilus T, Kaukinen K, Virta LJ, Pukkala E, Collin P. Incidence of malignancies in diagnosed celiac patients: a population-based estimate. Am J Gastroenterol. 2014;109(9):1471-7.
20. Kariniemi S, Rantalaiho V, Virta LJ, Kautiainen H, Puolakka K, Elfving P. Malignancies among newly diagnosed systemic lupus erythematosus patients and their survival. Lupus. 2022;31(14):1750-8.
21. Kingwell E, Bajdik C, Phillips N, Zhu F, Oger J, Hashimoto S, et al. Cancer risk in multiple sclerosis: findings from British Columbia, Canada. Brain. 2012;135(Pt 10):2973-9.
22. Koskinen I, Hervonen K, Pukkala E, Reunala T, Kaukinen K, Collin P. Cancer incidence and factors associated with malignancies in coeliac disease during long-term follow-up. GastroHep. 2021;3(2):107-15.
23. Krishnan A, Hadi YB, Shabih S, Mukherjee D, Patel RA, Patel R, et al. Risk of pancreatic cancer in individuals with celiac disease in the United States: A population-based matched cohort study. World J Gastrointest Oncol. 2023;15(3):523-32.
24. Landgren AM, Landgren O, Gridley G, Dores GM, Linet MS, Morton LM. Autoimmune disease and subsequent risk of developing alimentary tract cancers among 4.5 million US male veterans. Cancer. 2011;117(6):1163-71.
25. Lebwohl B, Green PHR, Emilsson L, Mårild K, Söderling J, Roelstraete B, et al. Cancer Risk in 47,241 Individuals With Celiac Disease: A Nationwide Cohort Study. Clin Gastroenterol Hepatol. 2022;20(2):e111-e31.
26. Liang JA, Sun LM, Yeh JJ, Lin WY, Chang SN, Sung HC, et al. Malignancies associated with systemic lupus erythematosus in Taiwan: A nationwide population-based cohort study. Rheumatology International. 2012;32(3):773-8.

27. Marrie RA, Maxwell C, Mahar A, Ekuma O, McClintock C, Seitz D, et al. Cancer Incidence and Mortality Rates in Multiple Sclerosis: A Matched Cohort Study. Neurology. 2021;96(4):e501-e12.
28. Mellemkjaer L, Andersen V, Linet MS, Gridley G, Hoover R, Olsen JH. Non-Hodgkin's lymphoma and other cancers among a cohort of patients with systemic lupus erythematosus. Arthritis Rheum. 1997;40(4):761-8.
29. Parikh-Patel A, White RH, Allen M, Cress R. Cancer risk in a cohort of patients with systemic lupus erythematosus (SLE) in California. Cancer Causes Control. 2008;19(8):887-94.
30. Pierret C, Mulliez A, Le Bihan-Benjamin C, Moisset X, Bousquet PJ, Leray E. Cancer Risk Among Patients With Multiple Sclerosis: A 10-Year Nationwide Retrospective Cohort Study. Neurology. 2024;103(9):e209885.
31. Shin S, Kim MH, Oh CM, Ha E, Ryoo JH. Association Between Type 1 Diabetes Mellitus and Incident Gastrointestinal Cancer in Korean Population: A Nationwide Retrospective Cohort Study. Diabetes Metab Res Rev. 2024;40(7):e3848.
32. Shu X, Ji J, Li X, Sundquist J, Sundquist K, Hemminki K. Cancer risk among patients hospitalized for Type 1 diabetes mellitus: a population-based cohort study in Sweden. Diabet Med. 2010;27(7):791-7.
33. Swerdlow AJ, Jones ME, Slater SD, Burden ACF, Botha JL, Waugh NR, et al. Cancer incidence and mortality in 23 000 patients with type 1 diabetes in the UK: Long-term follow-up. Int J Cancer. 2023;153(3):512-23.
34. Treppo E, Toffolutti F, Manfrè V, Taborelli M, De Marchi G, De Vita S, et al. Risk of Cancer in Connective Tissue Diseases in Northeastern Italy over 15 Years. J Clin Med. 2022;11(15).
35. Valent F. Diabetes mellitus and cancer of the digestive organs: An Italian population-based cohort study. J Diabetes Complications. 2015;29(8):1056-61.
36. Vicentini M, Ballotari P, Venturelli F, Ottone M, Manicardi V, Gallo M, et al. Impact of Insulin Therapies on Cancer Incidence in Type 1 and Type 2 Diabetes: A Population-Based Cohort Study in Reggio Emilia, Italy. CANCERS. 2022;14(11).
37. Viljamaa M, Kaukinen K, Pukkala E, Hervonen K, Reunala T, Collin P. Malignancies and mortality in patients with coeliac disease and dermatitis herpetiformis: 30-year population-based study. Digestive and Liver Disease. 2006;38(6):374-80.
38. Westermann R, Zobbe K, Cordtz R, Haugaard JH, Dreyer L. Increased cancer risk in patients with cutaneous lupus erythematosus and systemic lupus erythematosus compared with the general population: A Danish nationwide cohort study. Lupus. 2021;30(5):752-61.
39. Yuan F, Pfeiffer RM, Julian-Serrano S, Arjani S, Barrett MJ, Koshiol J, et al. Autoimmune conditions and pancreatic cancer risk in older American adults. Cancer Research. 2022;82(22 Supplement).
40. Zendehdel K, Nyrén O, Ostenson CG, Adami HO, Ekbom A, Ye W. Cancer incidence in patients with type 1 diabetes mellitus: a population-based cohort study in Sweden. J Natl Cancer Inst. 2003;95(23):1797-800.
41. Abu-Shakra M, Gladman DD, Urowitz MB. Malignancy in systemic lupus erythematosus. Arthritis Rheum. 1996;39(6):1050-4.
42. Bjornadal L, Lofstrom B, Yin L, Lundberg IE, Ekbom A. Increased cancer incidence in a Swedish cohort of patients with systemic lupus erythematosus. Scand J Rheumatol. 2002;31(2):66-71.
43. Grainge MJ, West J, Solaymani-Dodaran M, Card TR, Logan RF. The long-term risk of malignancy following a diagnosis of coeliac disease or dermatitis herpetiformis: a cohort study. Aliment Pharmacol Ther. 2012;35(6):730-9.
44. Cibere J, Sibley J, Haga M. Systemic lupus erythematosus and the risk of malignancy. Lupus. 2001;10(6):394-400.
45. Ragnarsson Ó, Gröndal G, Steinsson K. Risk of malignancy in an unselected cohort of Icelandic patients with systemic lupus erythematosus. Lupus. 2003;12(9):687-91.
46. Bahmanyar S, Montgomery SM, Hillert J, Ekbom A, Olsson T. Cancer risk among patients with multiple sclerosis and their parents. Neurology. 2009;72(13):1170-7.
47. Swerdlow AJ, Laing SP, Qiao Z, Slater SD, Burden AC, Botha JL, et al. Cancer incidence and mortality in patients with insulin-treated diabetes: a UK cohort study. Br J Cancer. 2005;92(11):2070-5.

Supplementary Figures

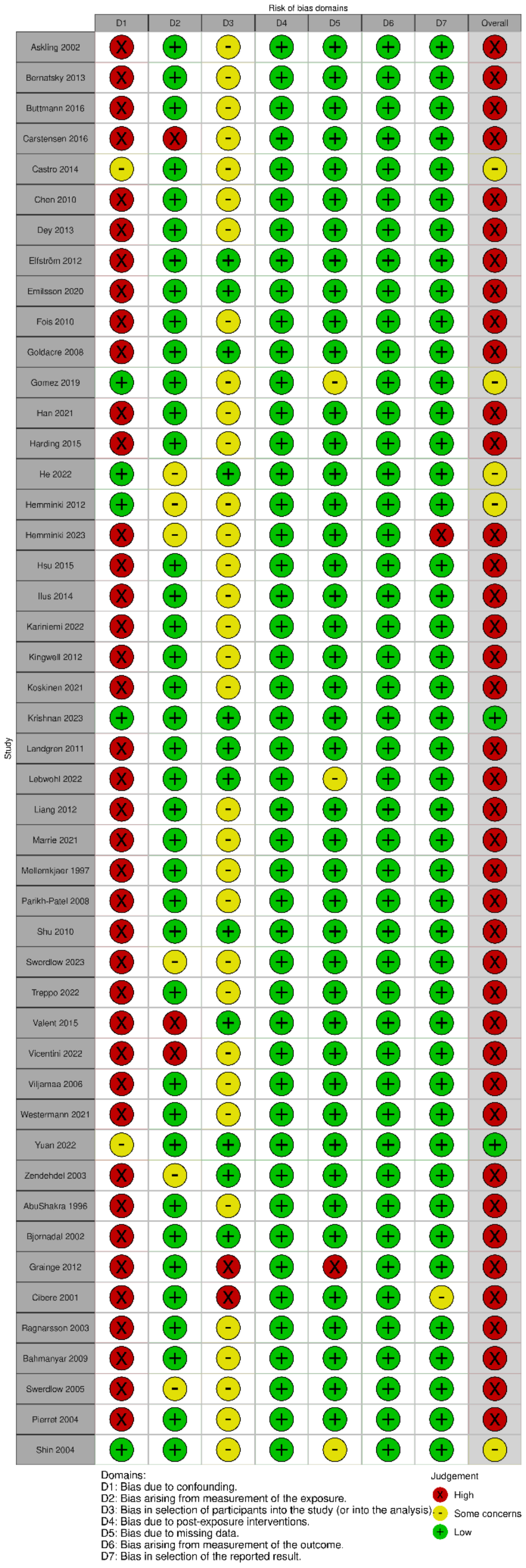

Figure 1: Study-specific risk of bias assessment using the ROBINS-E tool

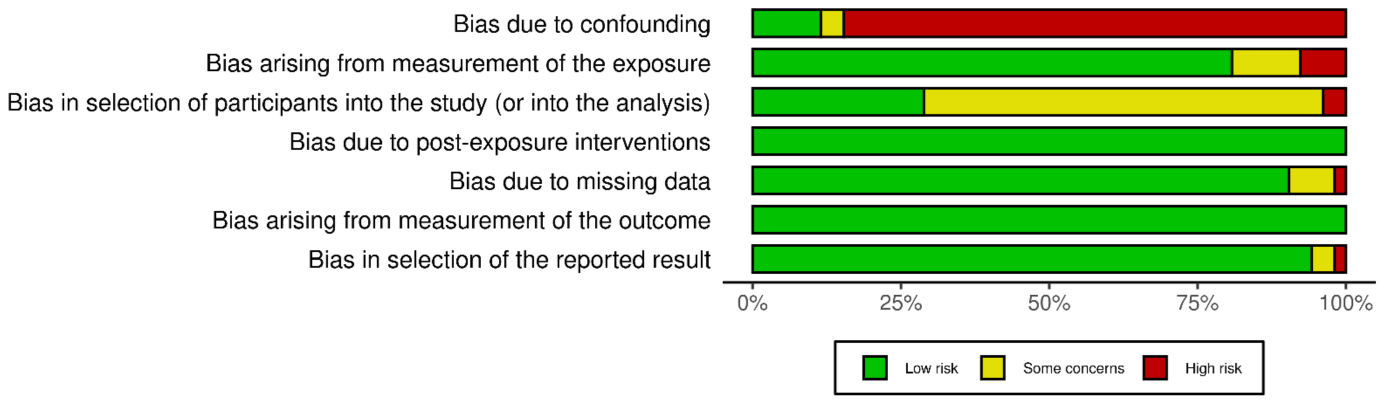

Figure 2: Distribution of studies regarding the seven domains of risk of bias based on the ROBINS-E tool

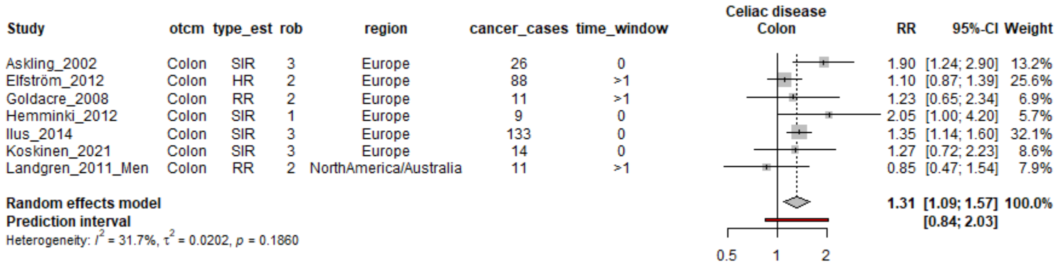
Celiac disease
Colon
Study otcm type_est rob region cancer_cases time_window RR 95%-CI Weight
Askling_2002 Colon SIR 3 Europe 26 0 1.90 [1.24; 2.90] 13.2%
Elfström_2012 Colon HR 2 Europe 88 >1 1.10 [0.87; 1.39] 25.6%
Goldacre_2008 Colon RR 2 Europe 11 >1 1.23 [0.65; 2.34] 6.9%
Hemminki_2012 Colon SIR 1 Europe 9 0 2.05 [1.00; 4.20] 5.7%
Ilus_2014 Colon SIR 3 Europe 133 0 1.35 [1.14; 1.60] 32.1%
Koskinen_2021 Colon SIR 3 Europe 14 0 1.27 [0.72; 2.23] 8.6%
Landgren_2011_Men Colon RR 2 NorthAmerica/Australia 11 >1 0.85 [0.47; 1.54] 7.9%
Random effects model 1.31 [1.09; 1.57] 100.0%
Prediction interval [0.84; 2.03]
Heterogeneity: I^2 = 31.7%, τ^2 = 0.0202, p = 0.1860
0.5 1 2

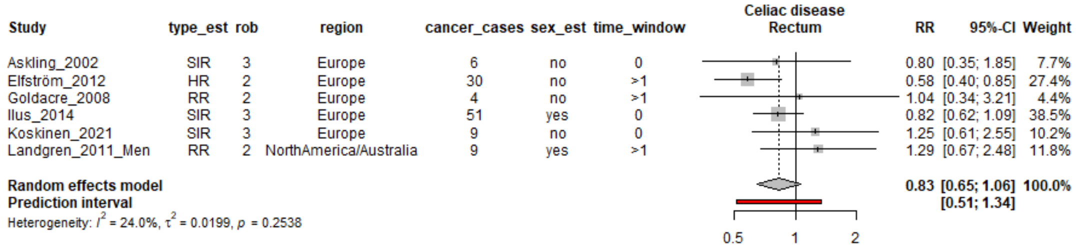
Celiac disease
Rectum
Study type_est rob region cancer_cases sex_est time_window RR 95%-CI Weight
Askling_2002 SIR 3 Europe 6 no 0 0.80 [0.35; 1.85] 7.7%
Elfström_2012 HR 2 Europe 30 no >1 0.58 [0.40; 0.85] 27.4%
Goldacre_2008 RR 2 Europe 4 no >1 1.04 [0.34; 3.21] 4.4%
Ilus_2014 SIR 3 Europe 51 yes 0 0.82 [0.62; 1.09] 38.5%
Koskinen_2021 SIR 3 Europe 9 no 0 1.25 [0.61; 2.55] 10.2%
Landgren_2011_Men RR 2 NorthAmerica/Australia 9 yes >1 1.29 [0.67; 2.48] 11.8%
Random effects model 0.83 [0.65; 1.06] 100.0%
Prediction interval [0.51; 1.34]
Heterogeneity: I^2 = 24.0%, τ^2 = 0.0199, p = 0.2538
0.5 1 2

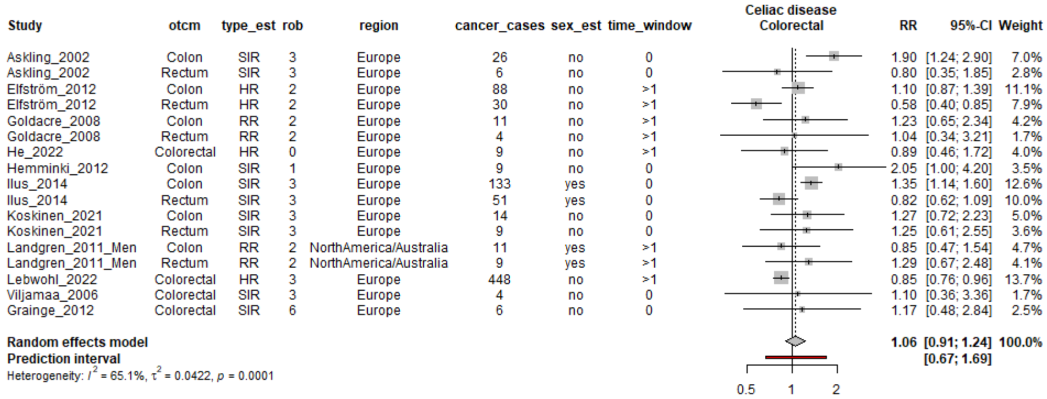
Celiac disease
Colorectal
Study otcm type_est rob region cancer_cases sex_est time_window RR 95%-CI Weight
Askling_2002 Colon SIR 3 Europe 26 no 0 1.90 [1.24; 2.90] 7.0%
Askling_2002 Rectum SIR 3 Europe 6 no 0 0.80 [0.35; 1.85] 2.8%
Elfström_2012 Colon HR 2 Europe 88 no >1 1.10 [0.87; 1.39] 11.1%
Elfström_2012 Rectum HR 2 Europe 30 no >1 0.58 [0.40; 0.85] 7.9%
Goldacre_2008 Colon RR 2 Europe 11 no >1 1.23 [0.65; 2.34] 4.2%
Goldacre_2008 Rectum RR 2 Europe 4 no >1 1.04 [0.34; 3.21] 1.7%
He_2022 Colorectal HR 0 Europe 9 no >1 0.89 [0.46; 1.72] 4.0%
Hemminki_2012 Colon SIR 1 Europe 9 no 0 2.05 [1.00; 4.20] 3.5%
Ilus_2014 Colon SIR 3 Europe 133 yes 0 1.35 [1.14; 1.60] 12.6%
Ilus_2014 Rectum SIR 3 Europe 51 yes 0 0.82 [0.62; 1.09] 10.0%
Koskinen_2021 Colon SIR 3 Europe 14 no 0 1.27 [0.72; 2.23] 5.0%
Koskinen_2021 Rectum SIR 3 Europe 9 no 0 1.25 [0.61; 2.55] 3.6%
Landgren_2011_Men Colon RR 2 NorthAmerica/Australia 11 yes >1 0.85 [0.47; 1.54] 4.7%
Landgren_2011_Men Rectum RR 2 NorthAmerica/Australia 9 yes >1 1.29 [0.67; 2.48] 4.1%
Lebwohl_2022 Colorectal HR 3 Europe 448 no >1 0.85 [0.76; 0.96] 13.7%
Viljamaa_2006 Colorectal SIR 3 Europe 4 no 0 1.10 [0.36; 3.36] 1.7%
Grainge_2012 Colorectal SIR 6 Europe 6 no 0 1.17 [0.48; 2.84] 2.5%
Random effects model 1.06 [0.91; 1.24] 100.0%
Prediction interval [0.67; 1.69]
Heterogeneity: I^2 = 65.1%, τ^2 = 0.0422, p = 0.0001
0.5 1 2

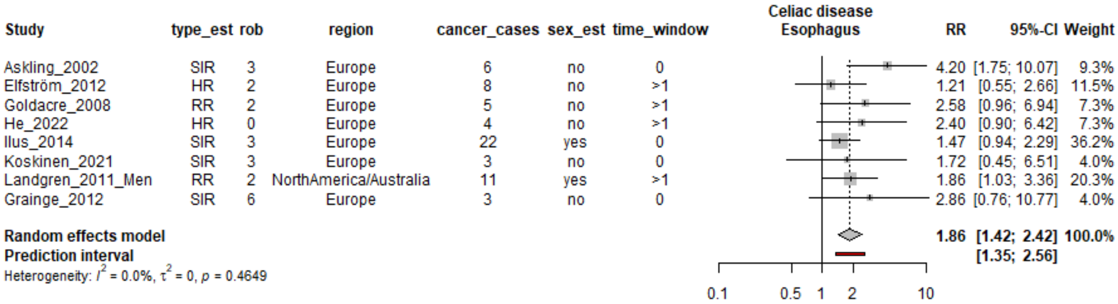
Celiac disease
Esophagus
Study type_est rob region cancer_cases sex_est time_window RR 95%-CI Weight
Askling_2002 SIR 3 Europe 6 no 0 4.20 [1.75; 10.07] 9.3%
Elfström_2012 HR 2 Europe 8 no >1 1.21 [0.55; 2.66] 11.5%
Goldacre_2008 RR 2 Europe 5 no >1 2.58 [0.96; 6.94] 7.3%
He_2022 HR 0 Europe 4 no >1 2.40 [0.90; 6.42] 7.3%
Ilus_2014 SIR 3 Europe 22 yes 0 1.47 [0.94; 2.29] 36.2%
Koskinen_2021 SIR 3 Europe 3 no 0 1.72 [0.45; 6.51] 4.0%
Landgren_2011_Men RR 2 NorthAmerica/Australia 11 yes >1 1.86 [1.03; 3.36] 20.3%
Grainge_2012 SIR 6 Europe 3 no 0 2.86 [0.76; 10.77] 4.0%
Random effects model 1.86 [1.42; 2.42] 100.0%
Prediction interval [1.35; 2.56]
Heterogeneity: I^2 = 0.0%, τ^2 = 0, p = 0.4649
0.1 0.5 1 2 10

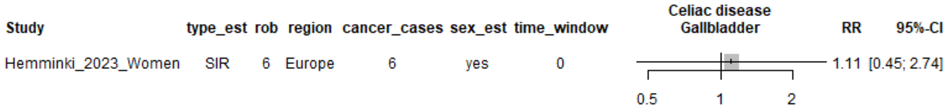
Celiac disease
Gallbladder
Study type_est rob region cancer_cases sex_est time_window RR 95%-CI
Hemminki_2023_Women SIR 6 Europe 6 yes 0 1.11 [0.45; 2.74]
0.5 1 2

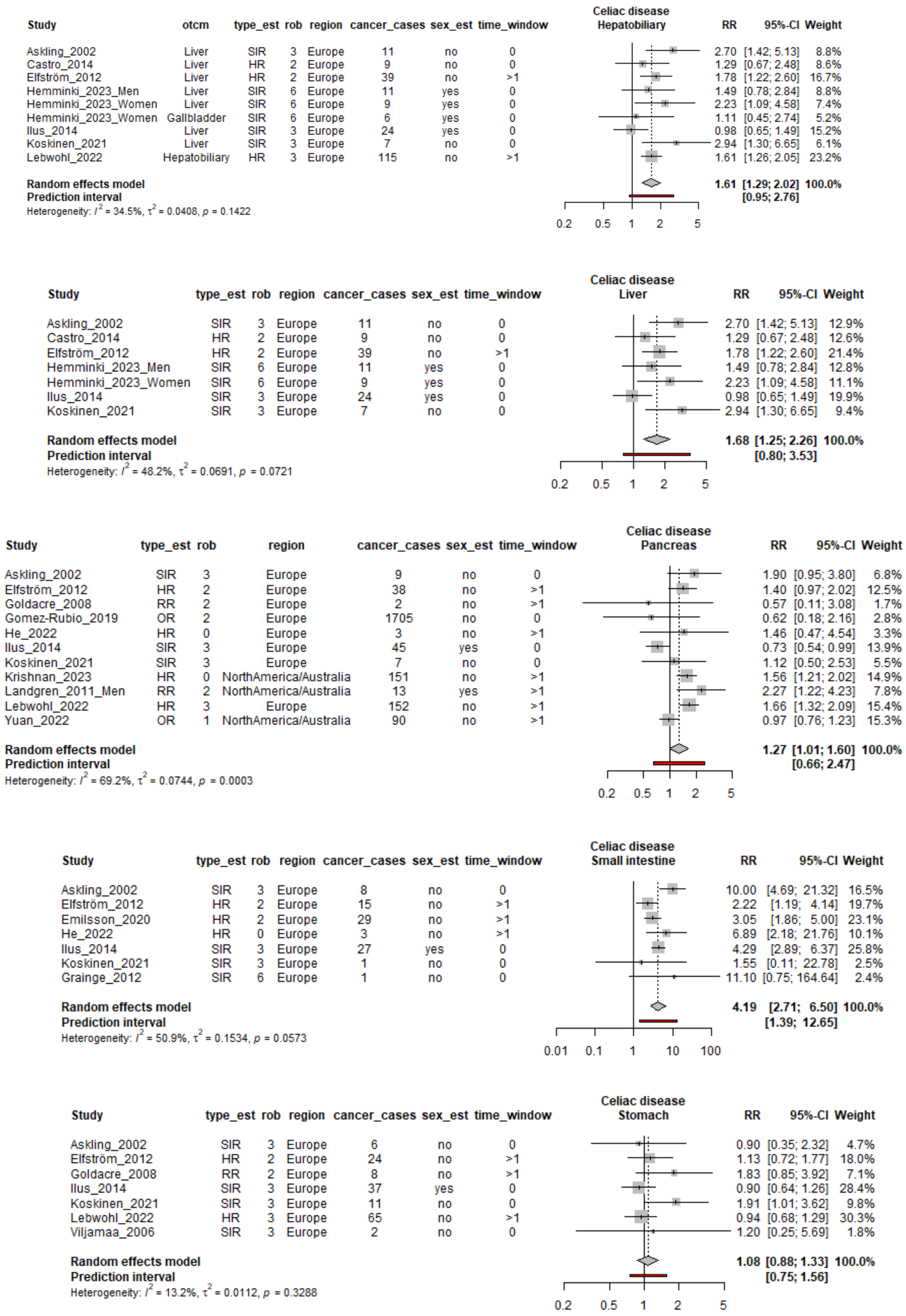

Figure 3: Unadjusted meta-analyses for the associations between celiac disease and digestive system cancers.

Assessed associations are displayed above the respective forest plots. The time window indicates whether a study left a period of at least 1 year between the diagnosis of exposure and outcome. Cancer cases represent the number of incident cancer cases in the autoimmune disease group. Sex_est indicates wether sex-specific estimates were presented in the respective study. Rob represents a weighted risk of bias score over the seven domains of ROBINS-E tool. Type_est shows the estimate-type used in the respective study (SIR=Standardized Incidence Ratio, RR=Relative Risk, HR=Hazard Ratio, OR=Odds Ratio, and IRR=Incidence Rate Ratio).

Study	type_est	rob	region	cancer_cases	sex_est	time_window	RR	95%-CI	Weight
Han_2021	SIR	3	Asia	61	no	0	1.73	[1.34; 2.23]	34.0%
Hemminki_2012	SIR	1	Europe	38	no	0	1.59	[1.14; 2.21]	25.7%
Landgren_2011_Men	RR	2	NorthAmerica/Australia	19	yes	>1	1.23	[0.76; 1.99]	15.5%
Abu-Shakra_1996	SIR	3	NorthAmerica/Australia	3	no	0	2.04	[0.54; 7.68]	2.6%
Bjornadal_2002	SIR	2	Europe	31	no	>1	0.94	[0.64; 1.38]	21.5%
Steinsson1,2_2003	SIR	3	Europe	1	no	0	0.88	[0.07; 11.18]	0.7%
**Random effects model**							**1.41**	**[1.13; 1.75]**	**100.0%**
**Prediction interval**								**[0.89; 2.22]**	

Heterogeneity: $I^2 = 37.4\%$, $\tau^2 = 0.0192$, $p = 0.1572$

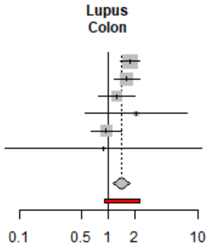

Study	otcm	type_est	rob	region	cancer_cases	sex_est	time_window	RR	95%-CI	Weight
Bernatsky_2013	Colorectal	SIR	3	Transethnic	51	no	0	0.88	[0.66; 1.17]	9.8%
Chen_2010	Colorectal	SIR	3	Asia	14	no	0	0.82	[0.78; 0.86]	16.0%
Han_2021	Colon	SIR	3	Asia	61	no	0	1.73	[1.34; 2.23]	10.7%
He_2022	Colorectal	HR	0	Europe	4	no	>1	1.60	[0.60; 4.27]	1.8%
Hemminki_2012	Colon	SIR	1	Europe	38	no	0	1.59	[1.14; 2.21]	8.6%
Hemminki_2012	Rectum	SIR	1	Europe	10	no	0	0.82	[0.42; 1.61]	3.4%
Kariniemi_2022	Colorectal	IRR	3	Europe	8	yes	0	2.05	[0.79; 5.33]	1.9%
Landgren_2011_Men	Colon	RR	2	NorthAmerica/Australia	19	yes	>1	1.23	[0.76; 1.99]	5.7%
Landgren_2011_Men	Rectum	RR	2	NorthAmerica/Australia	6	yes	>1	0.74	[0.33; 1.65]	2.6%
Liang_2012	Colorectal	HR	3	Asia	.	no	0	0.71	[0.30; 1.67]	2.3%
Mellemkjaer_1997	Colorectal	SIR	3	Europe	10	no	0	1.10	[0.56; 2.14]	3.5%
Parikh-Patel_2008	Colorectal	SIR	3	NorthAmerica/Australia	99	no	0	0.91	[0.74; 1.11]	12.2%
Westermann_2021	Colorectal	SIR	3	Europe	21	no	0	0.86	[0.56; 1.32]	6.5%
Abu-Shakra_1996	Colon	SIR	3	NorthAmerica/Australia	3	no	0	2.04	[0.54; 7.68]	1.1%
Bjornadal_2002	Colon	SIR	2	Europe	31	no	>1	0.94	[0.64; 1.38]	7.4%
Bjornadal_2002	Rectum	SIR	2	Europe	20	no	>1	1.07	[0.67; 1.70]	5.9%
Hga_2001	Colorectal	SIR	5	NorthAmerica/Australia	1	no	0	0.63	[0.03; 13.23]	0.2%
Steinsson1,2_2003	Colon	SIR	3	Europe	1	no	0	0.88	[0.07; 11.18]	0.3%
**Random effects model**								**1.05**	**[0.92; 1.21]**	**100.0%**
**Prediction interval**									**[0.70; 1.58]**	

Heterogeneity: $I^2 = 70.0\%$, $\tau^2 = 0.0315$, $p < 0.0001$

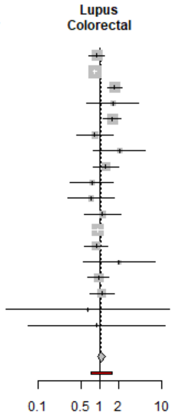

Study	type_est	rob	region	cancer_cases	sex_est	time_window	RR	95%-CI	Weight
Chen_2010	SIR	3	Asia	2	no	0	1.63	[1.42; 1.88]	86.8%
He_2022	HR	0	Europe	1	no	>1	2.70	[0.38; 19.19]	0.4%
Hemminki_2012	SIR	1	Europe	2	no	0	1.52	[0.24; 9.61]	0.5%
Landgren_2011_Men	RR	2	NorthAmerica/Australia	8	yes	>1	1.22	[0.58; 2.56]	3.2%
Parikh-Patel_2008	SIR	3	NorthAmerica/Australia	12	no	0	1.85	[1.00; 3.41]	4.6%
Westermann_2021	SIR	3	Europe	7	no	0	3.60	[1.59; 8.14]	2.6%
Bjornadal_2002	SIR	2	Europe	5	no	>1	1.59	[0.60; 4.25]	1.8%
**Random effects model**							**1.66**	**[1.46; 1.89]**	**100.0%**
**Prediction interval**								**[1.41; 1.96]**	

Heterogeneity: $I^2 = 0.0\%$, $\tau^2 = 0$, $p = 0.6018$

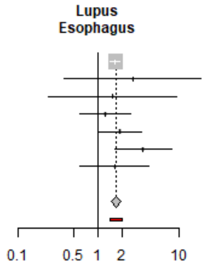

Study	type_est	rob	region	cancer_cases	sex_est	time_window	RR	95%-CI
Hemminki_2023_Women	SIR	6	Europe	7	yes	0	2.06	[0.90; 4.70]

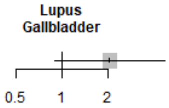

Study	otcm	type_est	rob	region	cancer_cases	sex_est	time_window	RR	95%-CI	Weight
Bernatsky_2013	Liver	SIR	3	Transethnic	12	no	0	1.87	[1.02; 3.43]	7.6%
Castro_2014	Liver	HR	2	Europe	17	no	0	1.36	[0.84; 2.20]	9.8%
Chen_2010	Hepatobiliary	SIR	3	Asia	28	no	0	1.83	[1.76; 1.90]	18.0%
Han_2021	Liver	SIR	3	Asia	63	no	0	1.86	[1.44; 2.39]	14.6%
He_2022	Liver	HR	0	Europe	1	no	>1	3.41	[0.48; 24.31]	1.2%
Hemminki_2023_Men	Liver	SIR	6	Europe	5	yes	0	3.50	[1.28; 9.57]	3.8%
Hemminki_2023_Women	Liver	SIR	6	Europe	9	yes	0	3.91	[1.90; 8.03]	6.2%
Hemminki_2023_Women	Gallbladder	SIR	6	Europe	7	yes	0	2.06	[0.90; 4.70]	5.1%
Liang_2012	Liver	HR	3	Asia	.	no	0	1.28	[0.66; 2.48]	6.9%
Mellemkjaer_1997	Liver	SIR	3	Europe	5	no	0	8.00	[2.99; 21.40]	3.9%
Parikh-Patel_2008	Liver	SIR	3	NorthAmerica/Australia	17	no	0	2.70	[1.63; 4.48]	9.3%
Treppo_2022	Hepatobiliary	SIR	3	Europe	1	no	0	0.54	[0.03; 9.37]	0.6%
Westermann_2021	Liver	SIR	3	Europe	4	no	0	2.47	[0.80; 7.59]	3.2%
Bjornadal_2002	Liver	SIR	2	Europe	17	no	>1	1.61	[0.97; 2.66]	9.3%
Hga_2001	Hepatobiliary	SIR	5	NorthAmerica/Australia	1	no	0	5.55	[0.26; 116.63]	0.5%
**Random effects model**								**2.08**	**[1.66; 2.59]**	**100.0%**
**Prediction interval**									**[1.12; 3.85]**	

Heterogeneity: $I^2 = 35.0\%$, $\tau^2 = 0.0703$, $p = 0.0885$

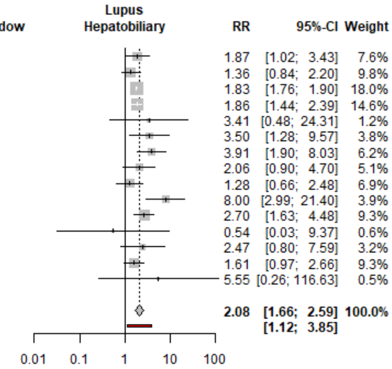

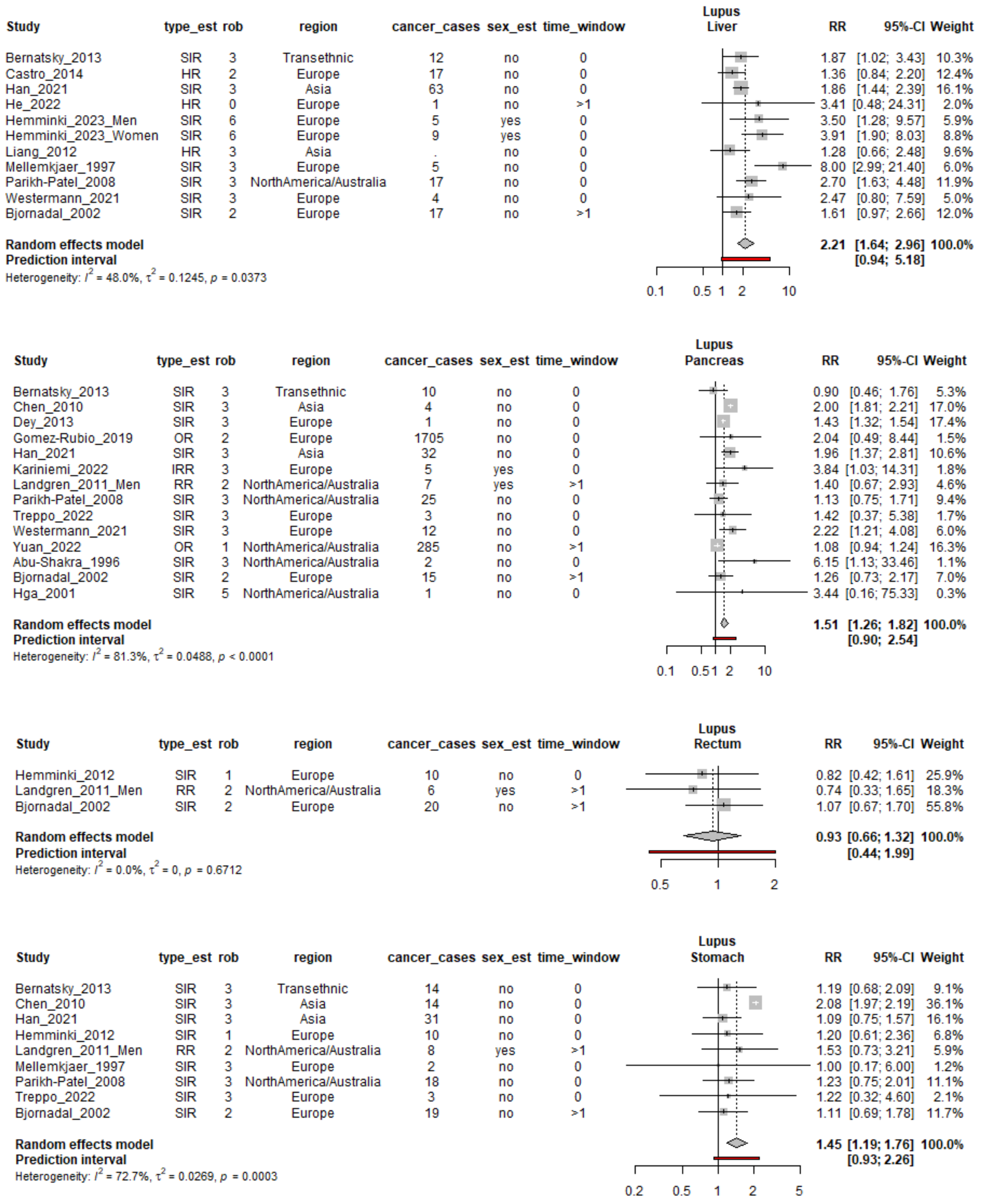

Figure 4: Unadjusted meta-analyses for the associations between systemic lupus erythematosus and digestive system cancers.

Assessed associations are displayed above the respective forest plots. The time window indicates whether a study left a period of at least 1 year between the diagnosis of exposure and outcome. Cancer cases represent the number of incident cancer cases in the autoimmune disease group. Sex_est indicates wether sex-specific estimates were presented in the respective study. Rob represents a weighted risk of bias score over the seven domains of ROBINS-E tool. Type_est shows the estimate-type used in the respective study (SIR=Standardized Incidence Ratio, RR=Relative Risk, HR=Hazard Ratio, OR=Odds Ratio, and IRR=Incidence Rate Ratio).

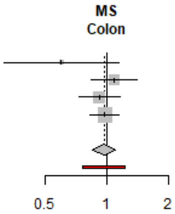

Study	type_est	rob	region	cancer_cases	sex_est	time_window	RR	95%-CI	Weight
Fois_2010	RR	3	Europe	13	no	0	0.60	[0.31; 1.15]	3.7%
Hemminki_2012	SIR	1	Europe	62	no	0	1.09	[0.84; 1.41]	22.0%
Landgren_2011_Men	RR	2	NorthAmerica/Australia	72	yes	>1	0.92	[0.73; 1.16]	26.3%
PhD_2009	HR	3	Europe	143	yes	0	0.98	[0.83; 1.15]	48.1%
**Random effects model**							**0.97**	**[0.86; 1.10]**	**100.0%**
**Prediction interval**								**[0.76; 1.23]**	

Heterogeneity: $I^2 = 3.8\%$, $\tau^2 = 0.0015$, $p = 0.3739$

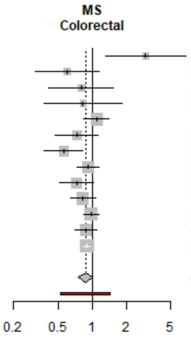

Study	otcm	type_est	rob	region	cancer_cases	sex_est	time_window	RR	95%-CI	Weight
Buttmann_2016	Colorectal	SIR	3	Europe	7	no	0	2.98	[1.32; 6.74]	2.8%
Fois_2010	Colon	RR	3	Europe	13	no	0	0.60	[0.31; 1.15]	4.0%
Fois_2010	Rectum	RR	3	Europe	11	no	0	0.80	[0.41; 1.55]	3.9%
He_2022	Colorectal	HR	0	Europe	6	no	>1	0.83	[0.37; 1.86]	2.9%
Hemminki_2012	Colon	SIR	1	Europe	62	no	0	1.09	[0.84; 1.41]	9.8%
Hemminki_2012	Rectum	SIR	1	Europe	23	no	0	0.73	[0.47; 1.13]	6.5%
Kingwell_2012	Colorectal	SIR	3	NorthAmerica/Australia	28	yes	0	0.56	[0.38; 0.83]	7.2%
Landgren_2011_Men	Colon	RR	2	NorthAmerica/Australia	72	yes	>1	0.92	[0.73; 1.16]	10.3%
Landgren_2011_Men	Rectum	RR	2	NorthAmerica/Australia	37	yes	>1	0.73	[0.52; 1.03]	8.1%
Marrie_2021	Colorectal	HR	3	NorthAmerica/Australia	.	no	0	0.83	[0.64; 1.07]	9.8%
PhD_2009	Colon	HR	3	Europe	143	yes	0	0.98	[0.83; 1.15]	11.6%
PhD_2009	Rectum	HR	3	Europe	72	yes	0	0.88	[0.70; 1.11]	10.3%
Pierret_2024	Colorectal	HR	3	Europe	651	yes	0	0.90	[0.84; 0.97]	13.0%
**Random effects model**								**0.87**	**[0.75; 1.02]**	**100.0%**
**Prediction interval**									**[0.53; 1.43]**	

Heterogeneity: $I^2 = 44.3\%$, $\tau^2 = 0.0462$, $p = 0.0430$

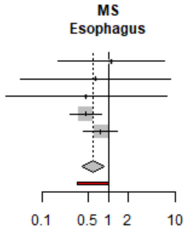

Study	type_est	rob	region	cancer_cases	sex_est	time_window	RR	95%-CI	Weight
Hemminki_2012	SIR	1	Europe	2	no	0	1.08	[0.17; 6.80]	4.4%
Kingwell_2012_Women	SIR	3	NorthAmerica/Australia	1	yes	0	0.63	[0.05; 8.37]	2.2%
Kingwell_2012_Men	SIR	3	NorthAmerica/Australia	1	yes	0	0.46	[0.03; 7.35]	1.9%
Landgren_2011_Men	RR	2	NorthAmerica/Australia	14	yes	>1	0.45	[0.26; 0.78]	49.1%
PhD_2009	HR	3	Europe	12	yes	0	0.76	[0.42; 1.37]	42.4%
**Random effects model**							**0.59**	**[0.40; 0.86]**	**100.0%**
**Prediction interval**								**[0.34; 1.02]**	

Heterogeneity: $I^2 = 0.0\%$, $\tau^2 = 0$, $p = 0.7198$

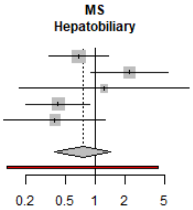

Study	otcm	type_est	rob	region	cancer_cases	sex_est	time_window	RR	95%-CI	Weight
Castro_2014	Liver	HR	2	Europe	9	no	0	0.68	[0.34; 1.36]	26.8%
Fois_2010	Liver	RR	3	Europe	6	no	0	2.20	[0.90; 5.39]	22.2%
He_2022	Liver	HR	0	Europe	1	no	>1	1.22	[0.17; 8.75]	8.5%
Landgren_2011_Men	Liver	RR	2	NorthAmerica/Australia	7	yes	>1	0.42	[0.20; 0.89]	25.5%
PhD_2009	Liver	HR	3	Europe	3	yes	0	0.39	[0.12; 1.26]	17.1%
**Random effects model**								**0.75**	**[0.39; 1.43]**	**100.0%**
**Prediction interval**									**[0.13; 4.26]**	

Heterogeneity: $I^2 = 57.1\%$, $\tau^2 = 0.2847$, $p = 0.0534$

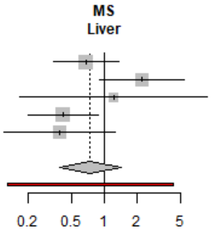

Study	type_est	rob	region	cancer_cases	sex_est	time_window	RR	95%-CI	Weight
Castro_2014	HR	2	Europe	9	no	0	0.68	[0.34; 1.36]	26.8%
Fois_2010	RR	3	Europe	6	no	0	2.20	[0.90; 5.39]	22.2%
He_2022	HR	0	Europe	1	no	>1	1.22	[0.17; 8.75]	8.5%
Landgren_2011_Men	RR	2	NorthAmerica/Australia	7	yes	>1	0.42	[0.20; 0.89]	25.5%
PhD_2009	HR	3	Europe	3	yes	0	0.39	[0.12; 1.26]	17.1%
**Random effects model**							**0.75**	**[0.39; 1.43]**	**100.0%**
**Prediction interval**								**[0.13; 4.26]**	

Heterogeneity: $I^2 = 57.1\%$, $\tau^2 = 0.2847$, $p = 0.0534$

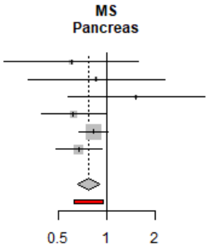

Study	type_est	rob	region	cancer_cases	sex_est	time_window	RR	95%-CI	Weight
Fois_2010	RR	3	Europe	5	no	0	0.60	[0.23; 1.59]	2.6%
Kingwell_2012_Women	SIR	3	NorthAmerica/Australia	5	yes	0	0.86	[0.32; 2.30]	2.6%
Kingwell_2012_Men	SIR	3	NorthAmerica/Australia	5	yes	0	1.53	[0.57; 4.09]	2.6%
Landgren_2011_Men	RR	2	NorthAmerica/Australia	18	yes	>1	0.62	[0.39; 0.99]	11.4%
Yuan_2022	OR	1	NorthAmerica/Australia	114	no	>1	0.83	[0.68; 1.02]	58.9%
PhD_2009	HR	3	Europe	32	yes	0	0.67	[0.48; 0.94]	22.0%
**Random effects model**							**0.77**	**[0.66; 0.90]**	**100.0%**
**Prediction interval**								**[0.63; 0.95]**	

Heterogeneity: $I^2 = 0.0\%$, $\tau^2 = 0$, $p = 0.5249$

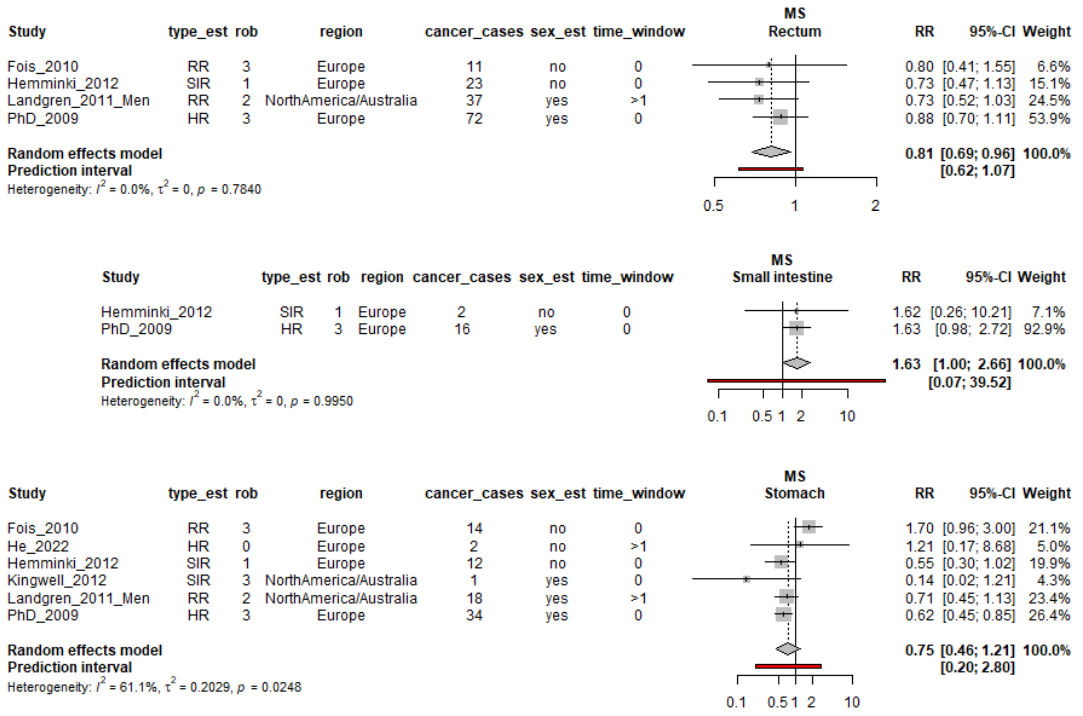

Figure 5: Unadjusted meta-analyses for the associations between multiple sclerosis and digestive system cancers.

Assessed associations are displayed above the respective forest plots. The time window indicates whether a study left a period of at least 1 year between the diagnosis of exposure and outcome. Cancer cases represent the number of incident cancer cases in the autoimmune disease group. Sex_est indicates wether sex-specific estimates were presented in the respective study. Rob represents a weighted risk of bias score over the seven domains of ROBINS-E tool. Type_est shows the estimate-type used in the respective study (SIR=Standardized Incidence Ratio, RR=Relative Risk, HR=Hazard Ratio, OR=Odds Ratio, and IRR=Incidence Rate Ratio).

Study	type_est	rob	region	cancer_cases	sex_est	time_window	RR	95%-CI	Weight
Carstensen_2016_Women	HR	5	Transethnic	483	yes	0	1.06	[0.93; 1.21]	20.6%
Carstensen_2016_Men	HR	5	Transethnic	273	yes	0	1.25	[1.11; 1.41]	21.4%
Hemminki_2012	SIR	2	Europe	4	no	0	0.96	[0.30; 3.03]	1.7%
Hsu_2015_Men	SIR	3	Asia	31	yes	0	1.50	[1.04; 2.17]	10.2%
Hsu_2015_Women	SIR	3	Asia	39	yes	0	1.95	[1.41; 2.70]	11.8%
Shu_2010	SIR	2	Europe	10	yes	>1	1.18	[0.60; 2.33]	4.3%
Valent_2015	HR	4	Europe	.	no	>1	1.81	[1.35; 2.43]	13.0%
Shin_2024	HR	2	Europe	100	yes	0	1.36	[1.11; 1.68]	17.1%
**Random effects model**							**1.37**	**[1.17; 1.60]**	**100.0%**
**Prediction interval**								**[0.90; 2.09]**	

Heterogeneity: $I^2 = 65.9\%$, $\tau^2 = 0.0257$, $p = 0.0045$

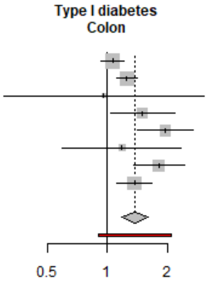

Study	otcm	type_est	rob	region	cancer_cases	sex_est	time_window	RR	95%-CI	Weight
Carstensen_2016_Women	Colon	HR	5	Transethnic	483	yes	0	1.06	[0.93; 1.21]	7.1%
Carstensen_2016_Men	Colon	HR	5	Transethnic	273	yes	0	1.25	[1.11; 1.41]	7.3%
Carstensen_2016_Women	Rectum	HR	5	Transethnic	272	yes	0	0.97	[0.81; 1.17]	6.5%
Carstensen_2016_Men	Rectum	HR	5	Transethnic	158	yes	0	0.96	[0.82; 1.12]	6.8%
Carstensen_2016_Women	Colorectal	HR	5	Transethnic	876	yes	0	1.09	[0.99; 1.21]	7.5%
Carstensen_2016_Men	Colorectal	HR	5	Transethnic	492	yes	0	1.14	[1.04; 1.24]	7.6%
Harding_2015	Colorectal	SIR	3	NorthAmerica/Australia	237	yes	0	1.21	[1.06; 1.38]	7.2%
He_2022	Colorectal	HR	1	Europe	28	no	>1	1.25	[0.85; 1.84]	3.9%
Hemminki_2012	Colon	SIR	2	Europe	4	no	0	0.96	[0.30; 3.03]	0.8%
Hemminki_2012	Rectum	SIR	2	Europe	5	no	0	2.06	[0.75; 5.62]	1.0%
Hsu_2015_Men	Colon	SIR	3	Asia	31	yes	0	1.50	[1.04; 2.17]	4.1%
Hsu_2015_Women	Colon	SIR	3	Asia	39	yes	0	1.95	[1.41; 2.70]	4.6%
Hsu_2015_Men	Rectum	SIR	3	Asia	27	yes	0	1.64	[1.10; 2.43]	3.8%
Hsu_2015_Women	Rectum	SIR	3	Asia	15	yes	0	1.07	[0.62; 1.84]	2.6%
Shu_2010	Colon	SIR	2	Europe	10	yes	>1	1.18	[0.60; 2.33]	1.9%
Shu_2010	Rectum	SIR	2	Europe	5	yes	>1	1.38	[0.51; 3.76]	1.0%
Swerdlow_2023	Colorectal	SIR	4	Europe	77	no	0	0.79	[0.63; 0.99]	5.9%
Valent_2015	Colon	HR	4	Europe	.	no	>1	1.81	[1.35; 2.43]	5.0%
Valent_2015	Rectum	HR	4	Europe	.	no	>1	1.89	[1.27; 2.82]	3.8%
Vicentini_2022	Colorectal	IRR	5	Europe	1	no	0	0.51	[0.07; 3.66]	0.3%
Zendehdel_2003	Colorectal	SIR	3	Europe	18	no	>1	1.10	[0.65; 1.85]	2.8%
Swerdlow_2005	Colorectal	SIR	4	Europe	14	no	0	0.71	[0.41; 1.24]	2.5%
Shin_2024	Colon	HR	2	Europe	100	yes	0	1.36	[1.11; 1.68]	6.2%
**Random effects model**								**1.21**	**[1.08; 1.34]**	**100.0%**
**Prediction interval**									**[0.79; 1.83]**	

Heterogeneity: $I^2 = 64.4\%$, $\tau^2 = 0.0372$, $p < 0.0001$

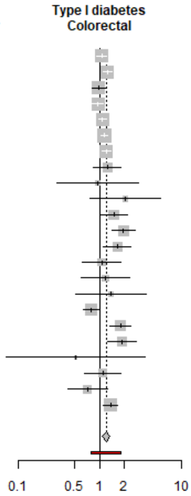

Study	type_est	rob	region	cancer_cases	sex_est	time_window	RR	95%-CI	Weight
Carstensen_2016_Women	HR	5	Transethnic	30	yes	0	1.79	[1.25; 2.56]	17.4%
Carstensen_2016_Men	HR	5	Transethnic	67	yes	0	1.08	[0.85; 1.37]	23.7%
Harding_2015	SIR	3	NorthAmerica/Australia	30	yes	0	1.75	[1.23; 2.50]	17.4%
He_2022	HR	1	Europe	9	no	>1	2.13	[1.13; 4.02]	8.6%
Swerdlow_2023	SIR	4	Europe	21	no	0	1.03	[0.65; 1.63]	13.3%
Valent_2015	HR	4	Europe	.	no	>1	2.26	[1.07; 4.76]	6.7%
Swerdlow_2005	SIR	4	Europe	4	no	0	1.06	[0.35; 3.25]	3.3%
Shin_2024	HR	2	Europe	12	yes	0	1.86	[1.04; 3.35]	9.6%
**Random effects model**							**1.49**	**[1.21; 1.85]**	**100.0%**
**Prediction interval**								**[0.89; 2.51]**	

Heterogeneity: $I^2 = 50.0\%$, $\tau^2 = 0.0359$, $p = 0.0511$

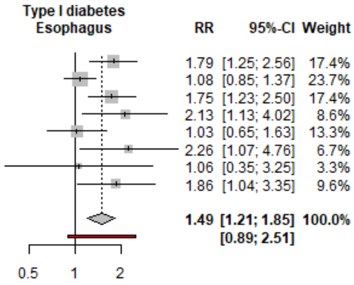

Study	type_est	rob	region	cancer_cases	sex_est	time_window	RR	95%-CI	Weight
Harding_2015	SIR	3	NorthAmerica/Australia	6	yes	0	0.81	[0.36; 1.81]	16.2%
Hemminki_2023_Men	SIR	6	Europe	28	yes	0	2.22	[1.50; 3.28]	30.0%
Hemminki_2023_Women	SIR	6	Europe	49	yes	0	1.85	[1.38; 2.47]	34.0%
Swerdlow_2023	SIR	4	Europe	2	no	0	0.52	[0.09; 2.88]	5.2%
Valent_2015	HR	4	Europe	.	no	>1	1.76	[0.73; 4.24]	14.5%
**Random effects model**							**1.59**	**[1.05; 2.41]**	**100.0%**
**Prediction interval**								**[0.53; 4.76]**	

Heterogeneity: $I^2 = 42.4\%$, $\tau^2 = 0.1111$, $p = 0.1386$

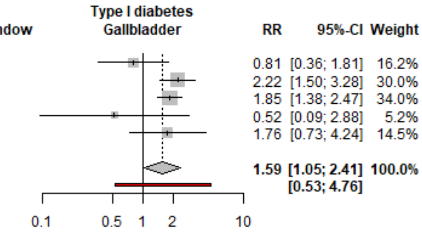

Study	otcm	type_est	rob	region	cancer_cases	sex_est	time_window	RR	95%-CI	Weight
Carstensen_2016_Women	Liver	HR	5	Transethnic	154	yes	0	1.55	[1.14; 2.10]	6.5%
Carstensen_2016_Men	Liver	HR	5	Transethnic	113	yes	0	2.00	[1.67; 2.40]	6.7%
Harding_2015	Gallbladder	SIR	3	NorthAmerica/Australia	6	yes	0	0.81	[0.36; 1.81]	4.9%
Harding_2015	Liver	SIR	3	NorthAmerica/Australia	53	yes	0	2.76	[2.11; 3.62]	6.6%
He_2022	Liver	HR	1	Europe	10	no	>1	2.82	[1.43; 5.56]	5.3%
Hemminki_2023_Men	Liver	SIR	6	Europe	376	yes	0	5.13	[4.63; 5.68]	6.8%
Hemminki_2023_Women	Liver	SIR	6	Europe	89	yes	0	4.92	[3.98; 6.09]	6.7%
Hemminki_2023_Men	Gallbladder	SIR	6	Europe	28	yes	0	2.22	[1.50; 3.28]	6.3%
Hemminki_2023_Women	Gallbladder	SIR	6	Europe	49	yes	0	1.85	[1.38; 2.47]	6.5%
Hsu_2015_Men	Liver	SIR	3	Asia	92	yes	0	1.63	[1.32; 2.01]	6.7%
Hsu_2015_Women	Liver	SIR	3	Asia	30	yes	0	1.00	[0.69; 1.45]	6.3%
Shu_2010	Liver	SIR	2	Europe	3	yes	>1	1.59	[0.40; 6.30]	3.1%
Swerdlow_2023	Liver	SIR	4	Europe	5	no	0	0.45	[0.17; 1.20]	4.3%
Swerdlow_2023	Gallbladder	SIR	4	Europe	2	no	0	0.52	[0.09; 2.88]	2.4%
Valent_2015	Liver	HR	4	Europe	.	no	>1	8.39	[6.57; 10.72]	6.6%
Valent_2015	Gallbladder	HR	4	Europe	.	no	>1	1.76	[0.73; 4.24]	4.6%
Vicentini_2022	Liver	IRR	5	Europe	1	no	0	2.00	[0.28; 14.29]	2.0%
Swerdlow_2005	Liver	SIR	4	Europe	1	no	0	0.70	[0.05; 9.80]	1.3%
Shin_2024	Liver	HR	2	Europe	90	yes	0	1.39	[1.12; 1.73]	6.7%
**Random effects model**								**1.99**	**[1.43; 2.76]**	**100.0%**
**Prediction interval**									**[0.50; 7.98]**	

Heterogeneity: $I^2 = 95.4\%$, $\tau^2 = 0.4090$, $p < 0.0001$

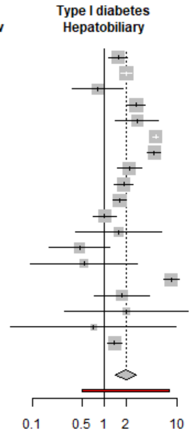

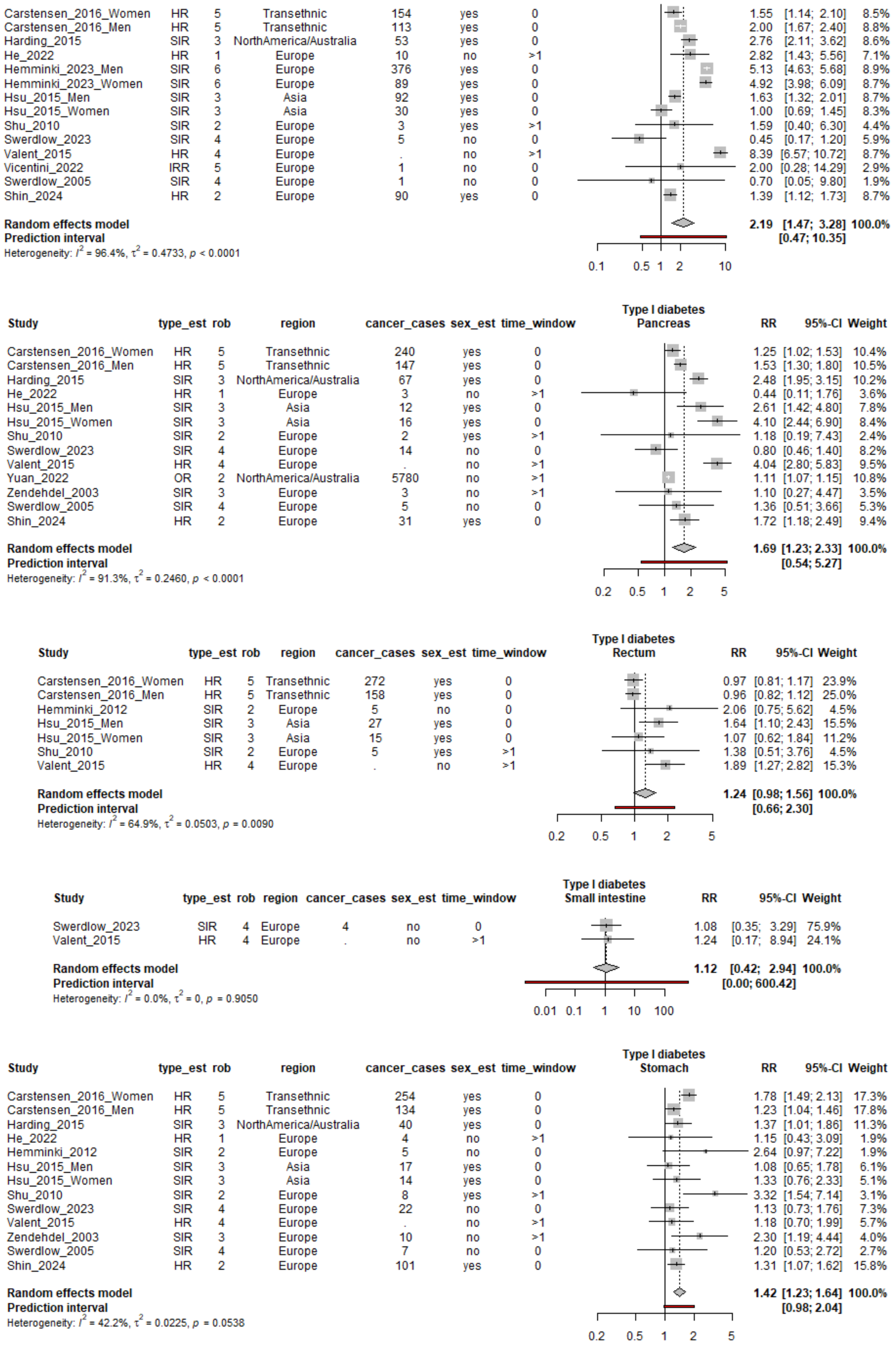

Figure 6: Unadjusted meta-analyses for the associations between type 1 diabetes mellitus and digestive system cancers.

Assessed associations are displayed above the respective forest plots. The time window indicates whether a study left a period of at least 1 year between the diagnosis of exposure and outcome. Cancer cases represent the number of incident cancer cases in the autoimmune disease group. Sex_est indicates wether sex-specific estimates were presented in the respective study. Rob represents a weighted risk of bias score over the seven domains of ROBINS-E tool. Type_est shows

the estimate-type used in the respective study (SIR=Standardized Incidence Ratio, RR=Relative Risk, HR=Hazard Ratio, OR=Odds Ratio, and IRR=Incidence Rate Ratio).

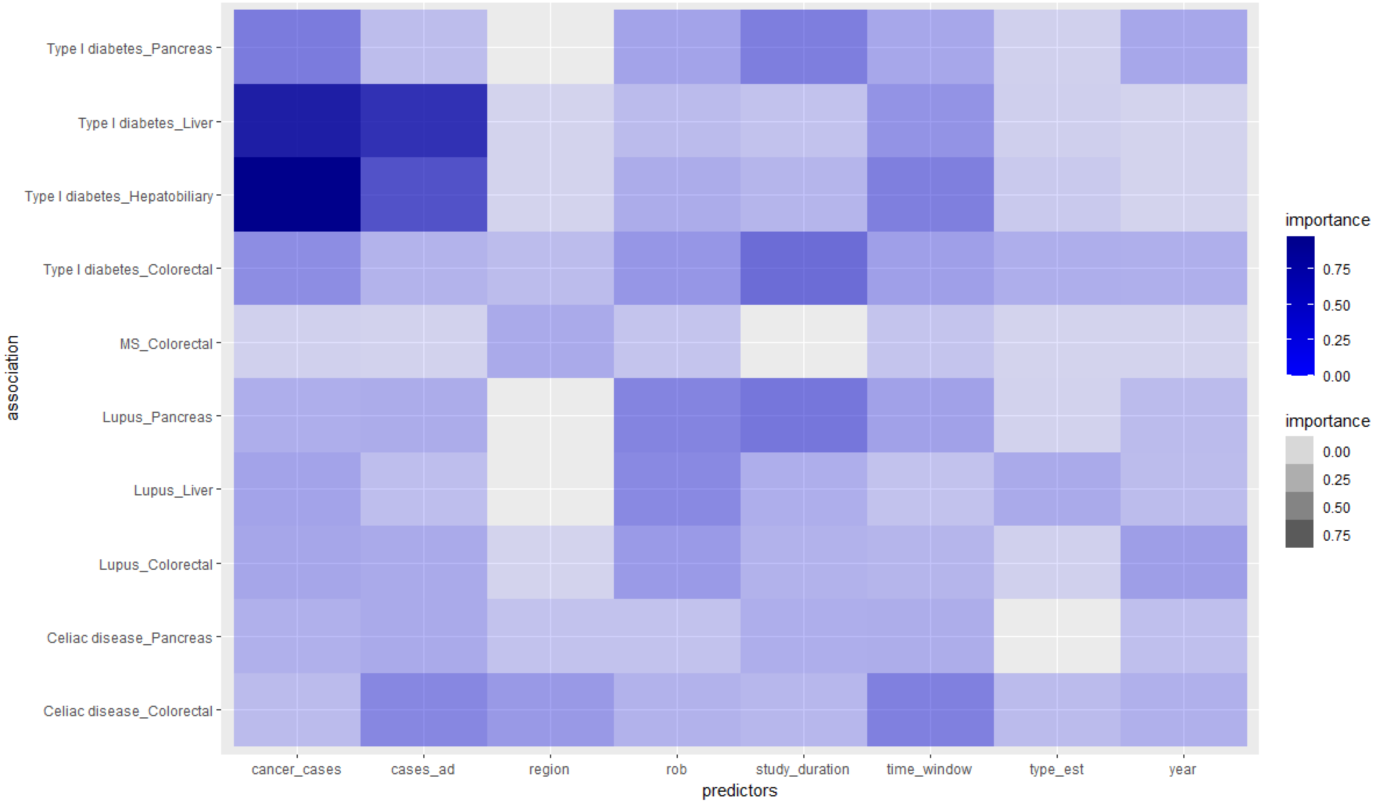

Figure 7: Predictor importance derived from multi-model inference

Stronger and darker colored areas correspond to higher predictor (x-axis) importance for investigated associations on the y-axis.

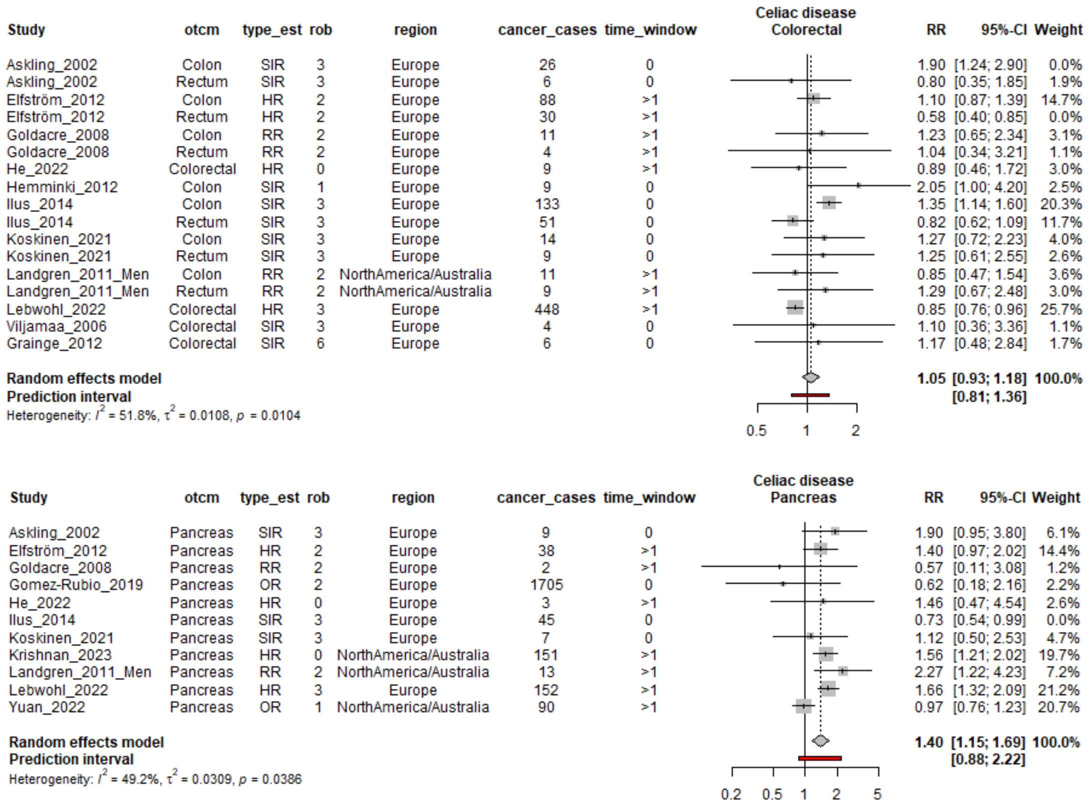

Figure 8: Outlier-adjusted meta-analyses for the associations between celiac disease and digestive system cancers.

Assessed associations are displayed above the respective forest plots. The time window indicates whether a study left a period of at least 1 year between the diagnosis of exposure and outcome. Cancer cases represent the number of cancer cases in the autoimmune disease group. Rob represents a weighted risk of bias score over the seven domains of ROBINS-E tool. Type_est shows the estimate-type used in the respective study (SIR=Standardized Incidence Ratio, RR=Relative Risk, HR=Hazard Ratio, OR=Odds Ratio, and IRR=Incidence Rate Ratio).

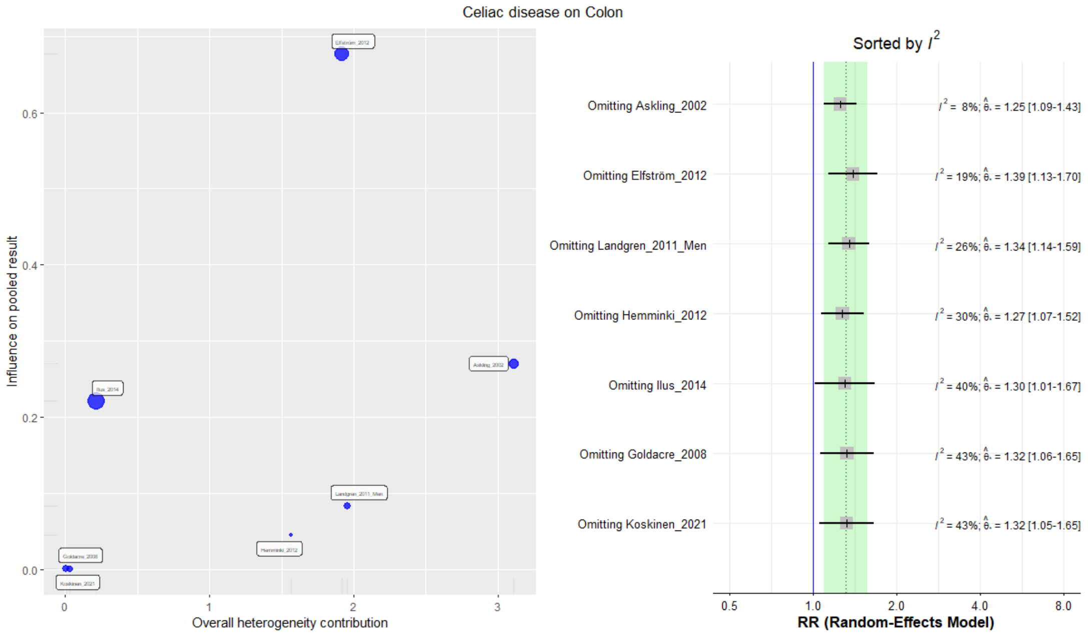

Celiac disease on Colorectal

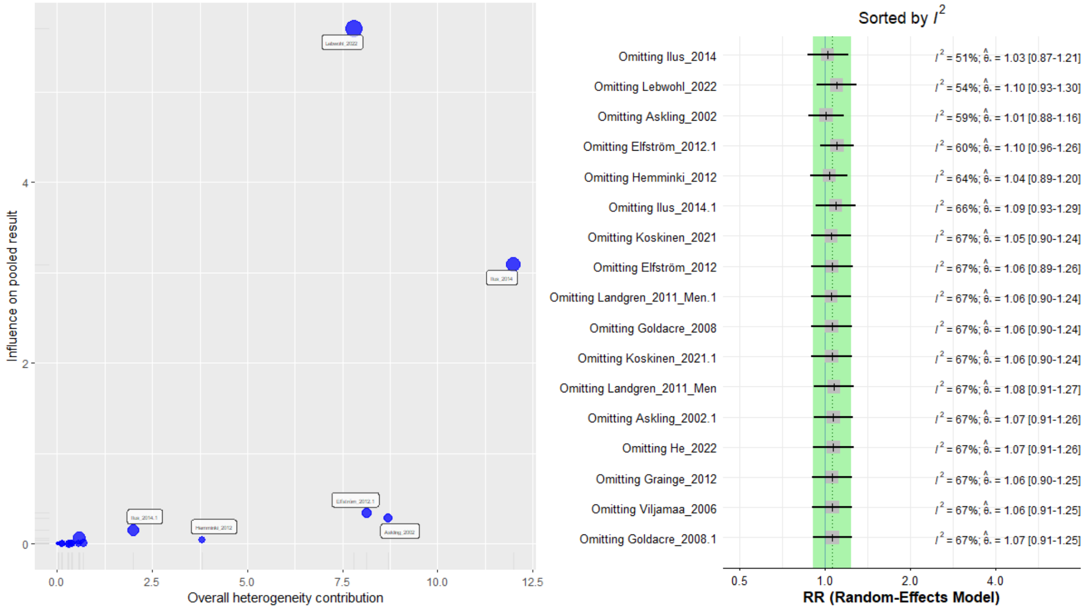

Celiac disease on Esophagus

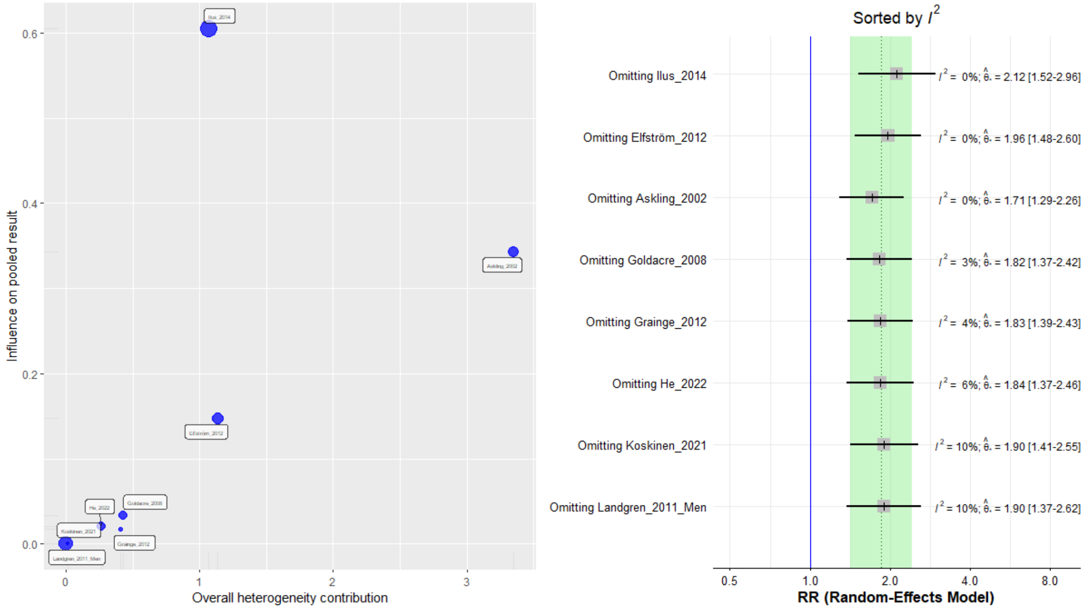

Celiac disease on Hepatobiliary

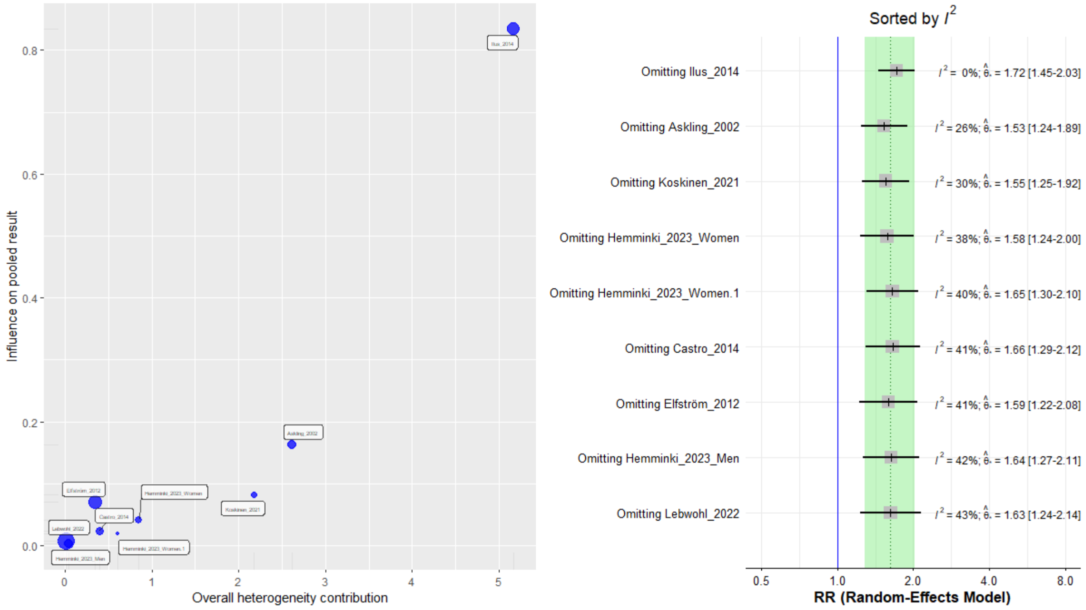

Celiac disease on Liver

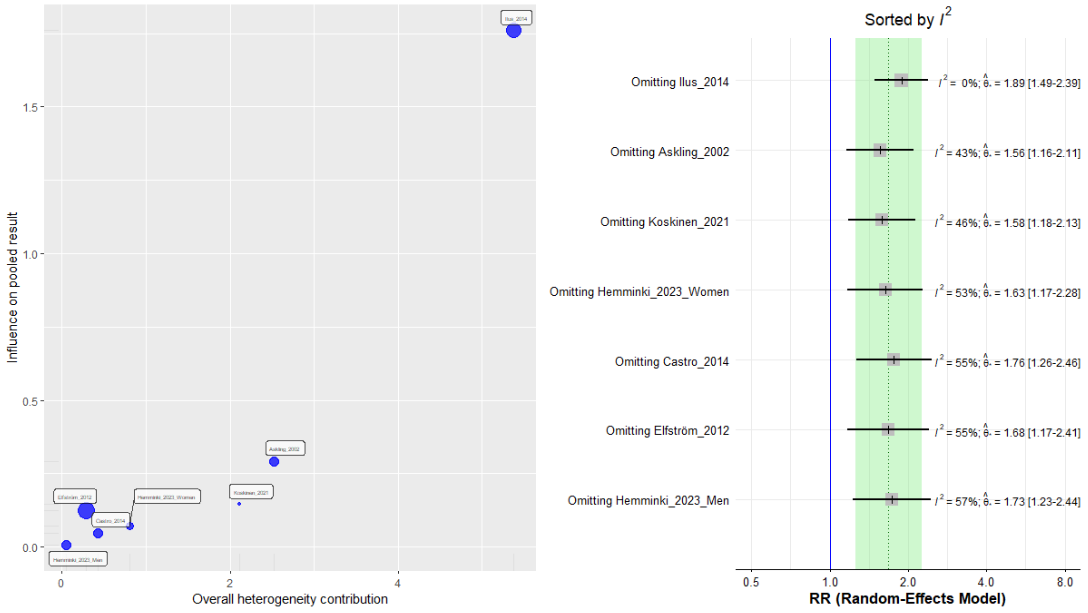

Celiac disease on Pancreas

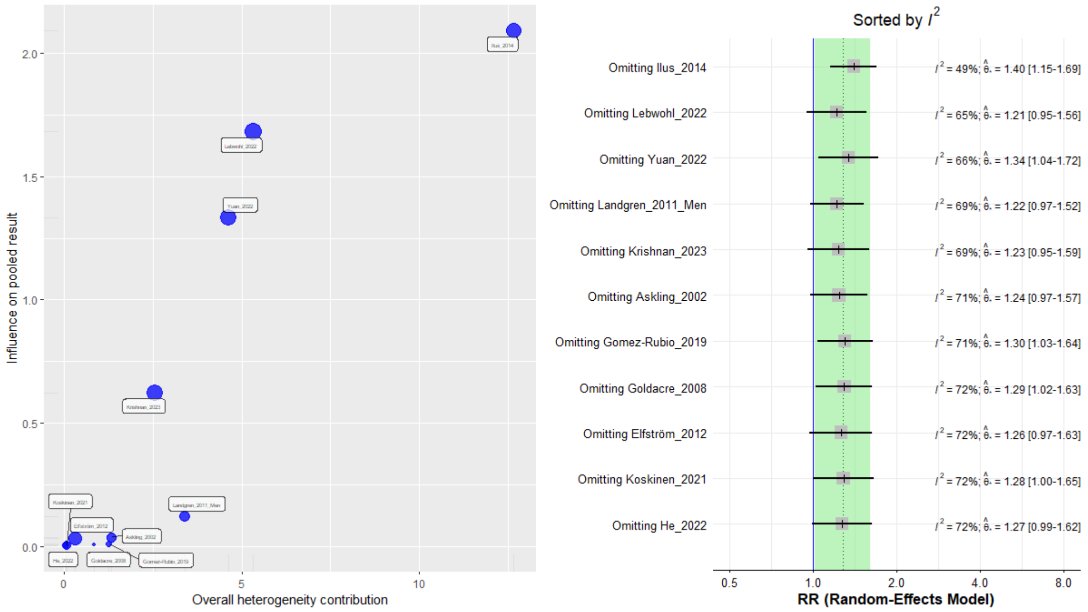

Celiac disease on Rectum

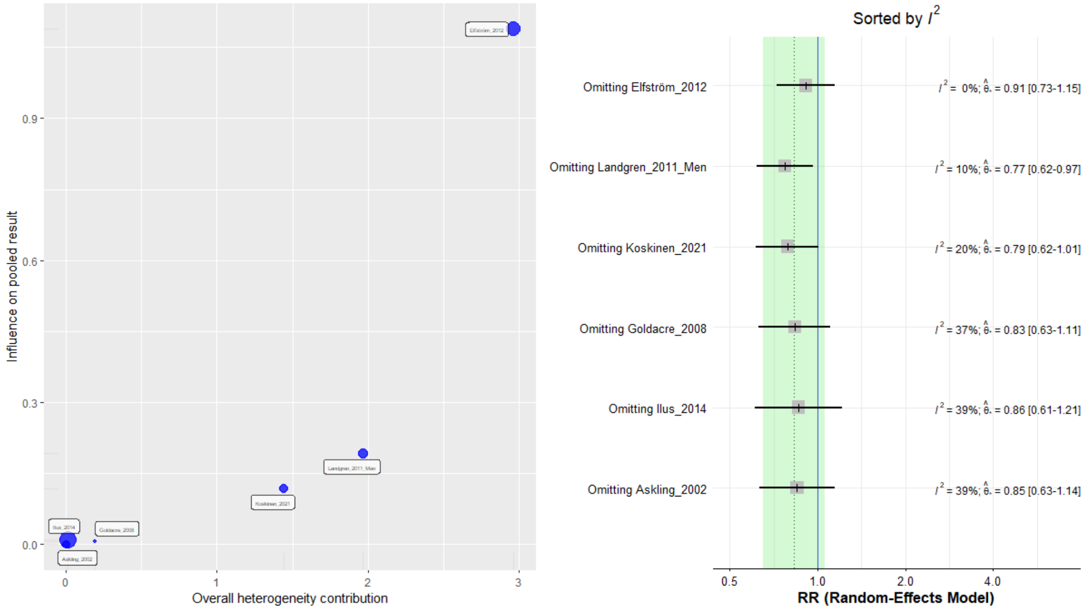

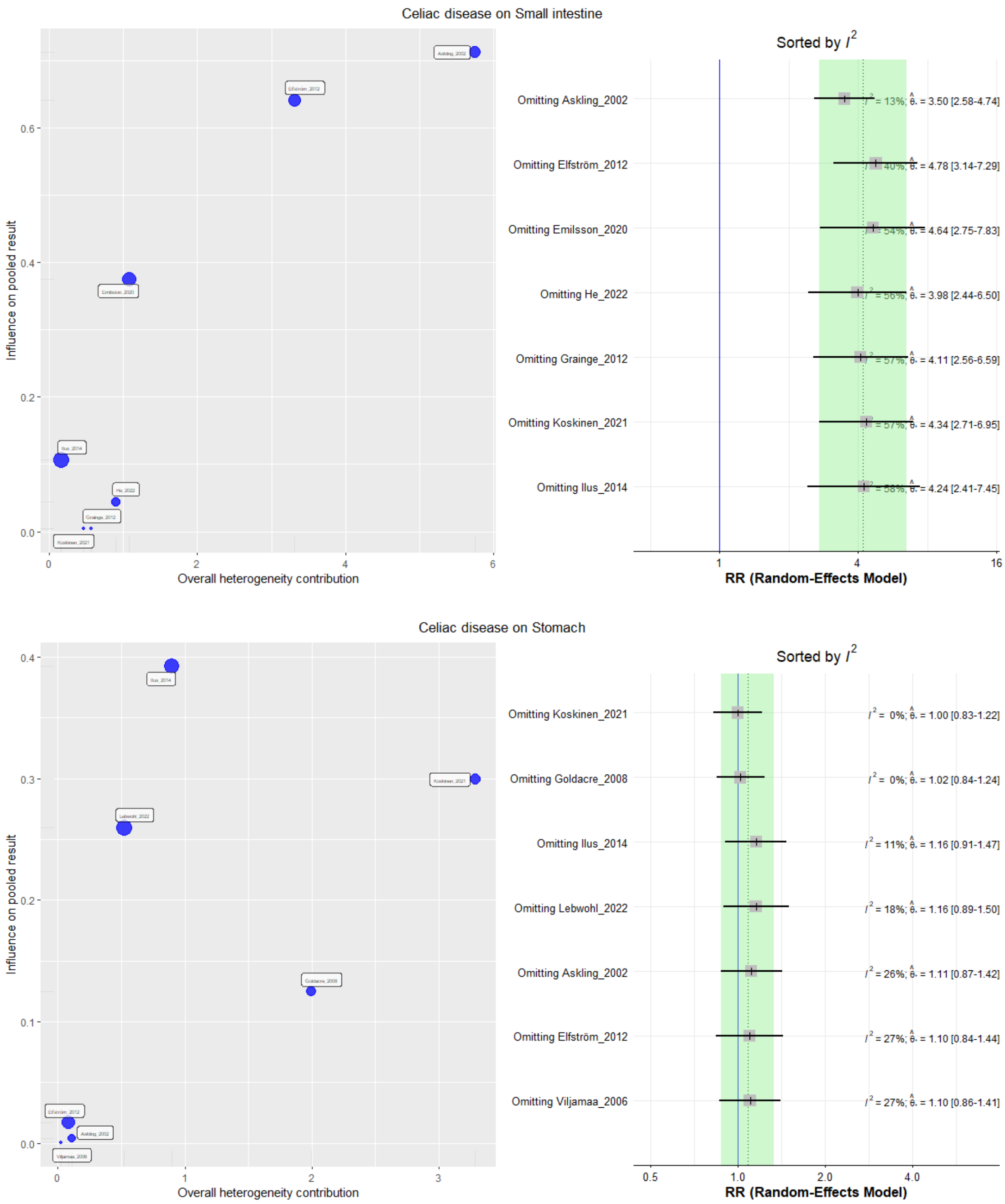

Figure 9: Influence analyses consisting of Baujat diagnostics and leave-one-out analyses (sorted by I^2) for the associations between celiac disease and different digestive system cancers.

Assessed associations are displayed above the respective plots.

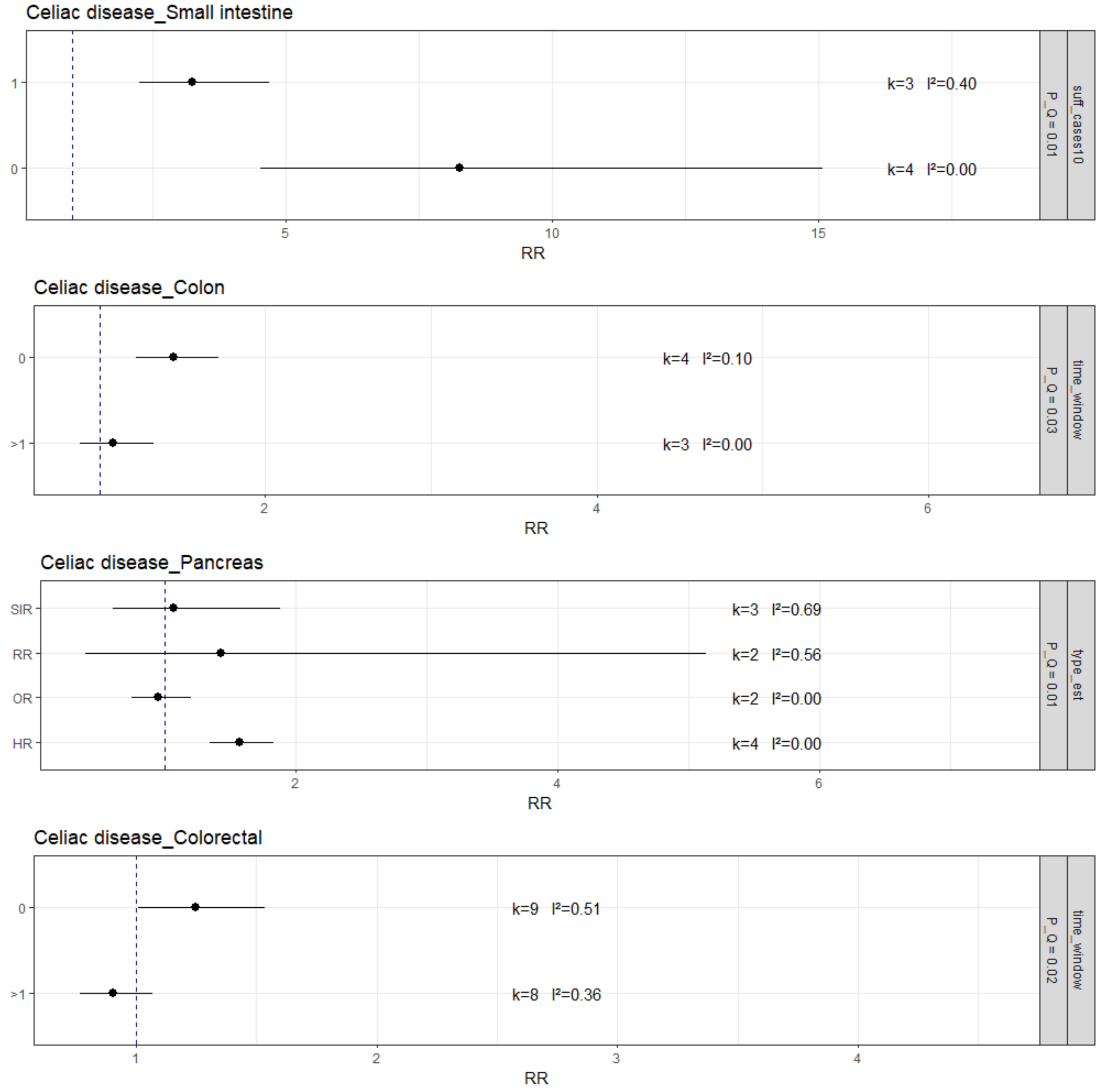

Figure 10: Subgroup analyses for the associations between celiac disease and digestive system cancers

The time window indicates whether a study left a period of at least 1 year between the diagnosis of exposure and outcome. Suff_cases10 indicates whether at least 10 cancer cases occurred within the respective autoimmune disease.

Study	otcm	type_est	rob	region	cancer_cases	time_window	RR	95%-CI	Weight
Bernatsky_2013	Colorectal	SIR	3	Transethnic	51	0	0.88	[0.66; 1.17]	15.7%
Chen_2010	Colorectal	SIR	3	Asia	14	0	0.82	[0.78; 0.86]	0.0%
Han_2021	Colon	SIR	3	Asia	61	0	1.73	[1.34; 2.23]	0.0%
He_2022	Colorectal	HR	0	Europe	4	>1	1.60	[0.60; 4.27]	1.5%
Hemminki_2012	Colon	SIR	1	Europe	38	0	1.59	[1.14; 2.21]	12.1%
Hemminki_2012	Rectum	SIR	1	Europe	10	0	0.82	[0.42; 1.61]	3.2%
Kariniemi_2022	Colorectal	IRR	3	Europe	8	0	2.05	[0.79; 5.33]	1.6%
Landgren_2011_Men	Colon	RR	2	NorthAmerica/Australia	19	>1	1.23	[0.76; 1.99]	6.1%
Landgren_2011_Men	Rectum	RR	2	NorthAmerica/Australia	6	>1	0.74	[0.33; 1.65]	2.2%
Liang_2012	Colorectal	HR	3	Asia	.	0	0.71	[0.30; 1.67]	2.0%
Mellemkjaer_1997	Colorectal	SIR	3	Europe	10	0	1.10	[0.56; 2.14]	3.2%
Parikh-Patel_2008	Colorectal	SIR	3	NorthAmerica/Australia	99	0	0.91	[0.74; 1.11]	27.5%
Westermann_2021	Colorectal	SIR	3	Europe	21	0	0.86	[0.56; 1.32]	7.6%
Abu-Shakra_1996	Colon	SIR	3	NorthAmerica/Australia	3	0	2.04	[0.54; 7.68]	0.8%
Bjornadal_2002	Colon	SIR	2	Europe	31	>1	0.94	[0.64; 1.38]	9.4%
Bjornadal_2002	Rectum	SIR	2	Europe	20	>1	1.07	[0.67; 1.70]	6.6%
Hga_2001	Colorectal	SIR	5	NorthAmerica/Australia	1	0	0.63	[0.03; 13.23]	0.2%
Steinsson1,2_2003	Colon	SIR	3	Europe	1	0	0.88	[0.07; 11.18]	0.2%
**Random effects model**							**1.02**	**[0.90; 1.15]**	**100.0%**
**Prediction interval**								**[0.85; 1.22]**	

Heterogeneity: $I^2 = 8.1\%$, $\tau^2 = 0.0034$, $p = 0.3612$

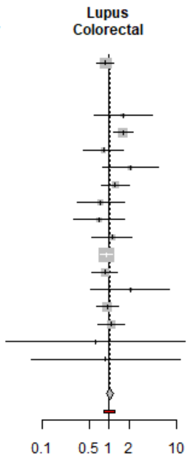

Study	otcm	type_est	rob	region	cancer_cases	time_window	RR	95%-CI	Weight
Bernatsky_2013	Liver	SIR	3	Transethnic	12	0	1.87	[1.02; 3.43]	0.4%
Castro_2014	Liver	HR	2	Europe	17	0	1.36	[0.84; 2.20]	0.6%
Chen_2010	Hepatobiliary	SIR	3	Asia	28	0	1.83	[1.76; 1.90]	94.7%
Han_2021	Liver	SIR	3	Asia	63	0	1.86	[1.44; 2.39]	2.2%
He_2022	Liver	HR	0	Europe	1	>1	3.41	[0.48; 24.31]	0.0%
Hemminki_2023_Men	Liver	SIR	6	Europe	5	0	3.50	[1.28; 9.57]	0.1%
Hemminki_2023_Women	Liver	SIR	6	Europe	9	0	3.91	[1.90; 8.03]	0.3%
Hemminki_2023_Women	Gallbladder	SIR	6	Europe	7	0	2.06	[0.90; 4.70]	0.2%
Liang_2012	Liver	HR	3	Asia	.	0	1.28	[0.66; 2.48]	0.3%
Mellemkjaer_1997	Liver	SIR	3	Europe	5	0	8.00	[2.99; 21.40]	0.0%
Parikh-Patel_2008	Liver	SIR	3	NorthAmerica/Australia	17	0	2.70	[1.63; 4.48]	0.5%
Treppo_2022	Hepatobiliary	SIR	3	Europe	1	0	0.54	[0.03; 9.37]	0.0%
Westermann_2021	Liver	SIR	3	Europe	4	0	2.47	[0.80; 7.59]	0.1%
Bjornadal_2002	Liver	SIR	2	Europe	17	>1	1.61	[0.97; 2.66]	0.5%
Hga_2001	Hepatobiliary	SIR	5	NorthAmerica/Australia	1	0	5.55	[0.26; 116.63]	0.0%
**Random effects model**							**1.83**	**[1.77; 1.90]**	**100.0%**
**Prediction interval**								**[1.76; 1.91]**	

Heterogeneity: $I^2 = 0.0\%$, $\tau^2 = 0$, $p = 0.4518$

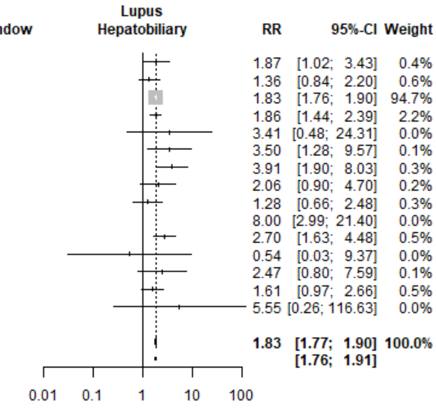

Study	otcm	type_est	rob	region	cancer_cases	time_window	RR	95%-CI	Weight
Bernatsky_2013	Liver	SIR	3	Transethnic	12	0	1.87	[1.02; 3.43]	9.6%
Castro_2014	Liver	HR	2	Europe	17	0	1.36	[0.84; 2.20]	13.6%
Han_2021	Liver	SIR	3	Asia	63	0	1.86	[1.44; 2.39]	27.2%
He_2022	Liver	HR	0	Europe	1	>1	3.41	[0.48; 24.31]	1.1%
Hemminki_2023_Men	Liver	SIR	6	Europe	5	0	3.50	[1.28; 9.57]	4.1%
Hemminki_2023_Women	Liver	SIR	6	Europe	9	0	3.91	[1.90; 8.03]	7.3%
Liang_2012	Liver	HR	3	Asia	.	0	1.28	[0.66; 2.48]	8.4%
Mellemkjaer_1997	Liver	SIR	3	Europe	5	0	8.00	[2.99; 21.40]	0.0%
Parikh-Patel_2008	Liver	SIR	3	NorthAmerica/Australia	17	0	2.70	[1.63; 4.48]	12.6%
Westermann_2021	Liver	SIR	3	Europe	4	0	2.47	[0.80; 7.59]	3.3%
Bjornadal_2002	Liver	SIR	2	Europe	17	>1	1.61	[0.97; 2.66]	12.7%
**Random effects model**							**1.96**	**[1.58; 2.42]**	**100.0%**
**Prediction interval**								**[1.25; 3.08]**	

Heterogeneity: $I^2 = 20.8\%$, $\tau^2 = 0.0267$, $p = 0.2514$

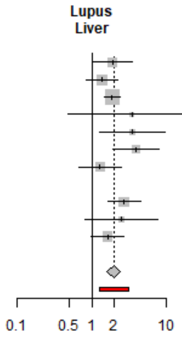

Study	otcm	type_est	rob	region	cancer_cases	time_window	RR	95%-CI	Weight
Bernatsky_2013	Pancreas	SIR	3	Transethnic	10	0	0.90	[0.46; 1.76]	5.4%
Chen_2010	Pancreas	SIR	3	Asia	4	0	2.00	[1.81; 2.21]	23.4%
Dey_2013	Pancreas	SIR	3	Europe	1	0	1.43	[1.32; 1.54]	24.1%
Gomez-Rubio_2019	Pancreas	OR	2	Europe	1705	0	2.04	[0.49; 8.44]	1.4%
Han_2021	Pancreas	SIR	3	Asia	32	0	1.96	[1.37; 2.81]	12.3%
Kariniemi_2022	Pancreas	IRR	3	Europe	5	0	3.84	[1.03; 14.31]	1.7%
Landgren_2011_Men	Pancreas	RR	2	NorthAmerica/Australia	7	>1	1.40	[0.67; 2.93]	4.6%
Parikh-Patel_2008	Pancreas	SIR	3	NorthAmerica/Australia	25	0	1.13	[0.75; 1.71]	10.5%
Treppo_2022	Pancreas	SIR	3	Europe	3	0	1.42	[0.37; 5.38]	1.6%
Westermann_2021	Pancreas	SIR	3	Europe	12	0	2.22	[1.21; 4.08]	6.3%
Yuan_2022	Pancreas	OR	1	NorthAmerica/Australia	285	>1	1.08	[0.94; 1.24]	0.0%
Abu-Shakra_1996	Pancreas	SIR	3	NorthAmerica/Australia	2	0	6.15	[1.13; 33.46]	1.0%
Bjornadal_2002	Pancreas	SIR	2	Europe	15	>1	1.26	[0.73; 2.17]	7.4%
Hga_2001	Pancreas	SIR	5	NorthAmerica/Australia	1	0	3.44	[0.16; 75.33]	0.3%
**Random effects model**							**1.62**	**[1.36; 1.93]**	**100.0%**
**Prediction interval**								**[1.04; 2.51]**	

Heterogeneity: $I^2 = 70.4\%$, $\tau^2 = 0.0317$, $p < 0.0001$

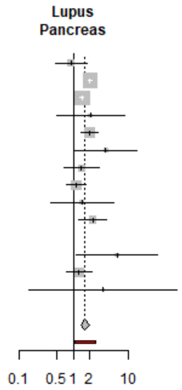

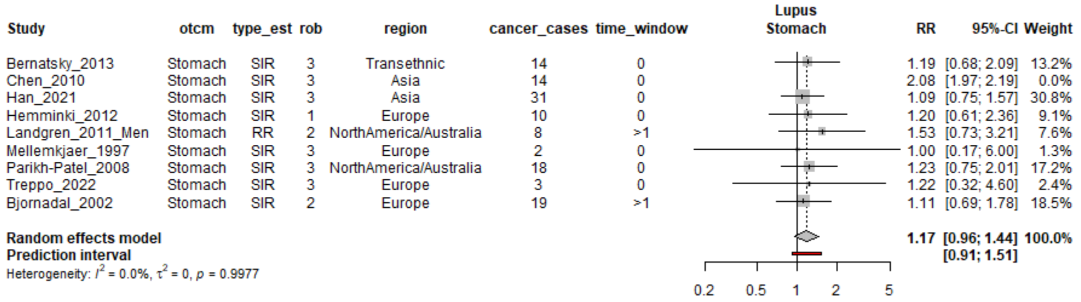

Figure 11: Outlier-adjusted meta-analyses for the associations between systemic lupus erythematosus and digestive system cancers.

Assessed associations are displayed above the respective forest plots. The time window indicates whether a study left a period of at least 1 year between the diagnosis of exposure and outcome. Cancer cases represent the number of incident cancer cases in the autoimmune disease group. Rob represents a weighted risk of bias score over the seven domains of ROBINS-E tool. Type_est shows the estimate-type used in the respective study (SIR=Standardized Incidence Ratio, RR=Relative Risk, HR=Hazard Ratio, OR=Odds Ratio, and IRR=Incidence Rate Ratio).

Lupus on Colon

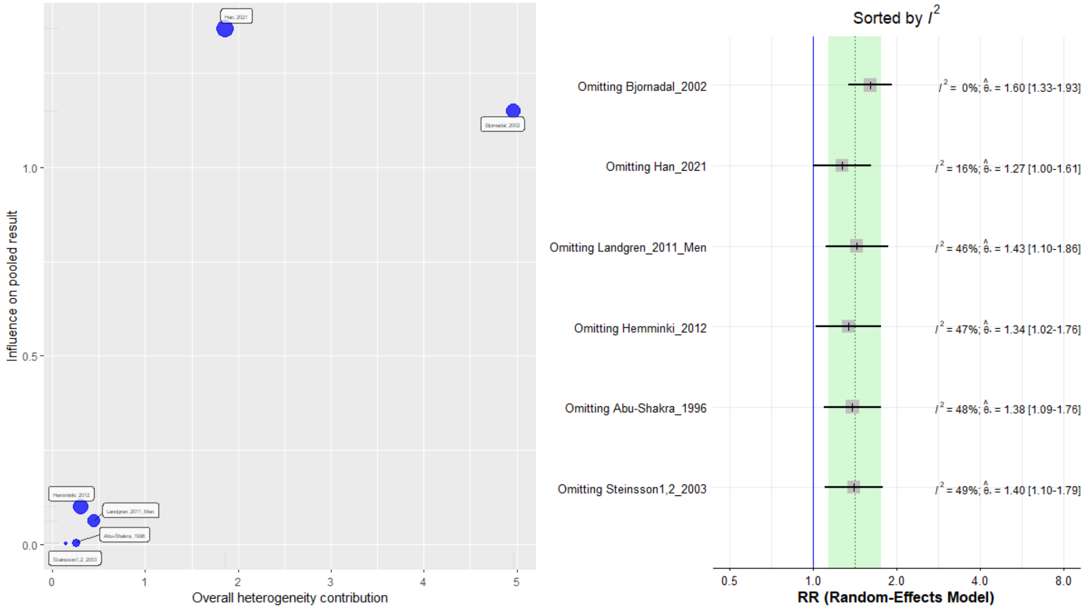

Lupus on Colorectal

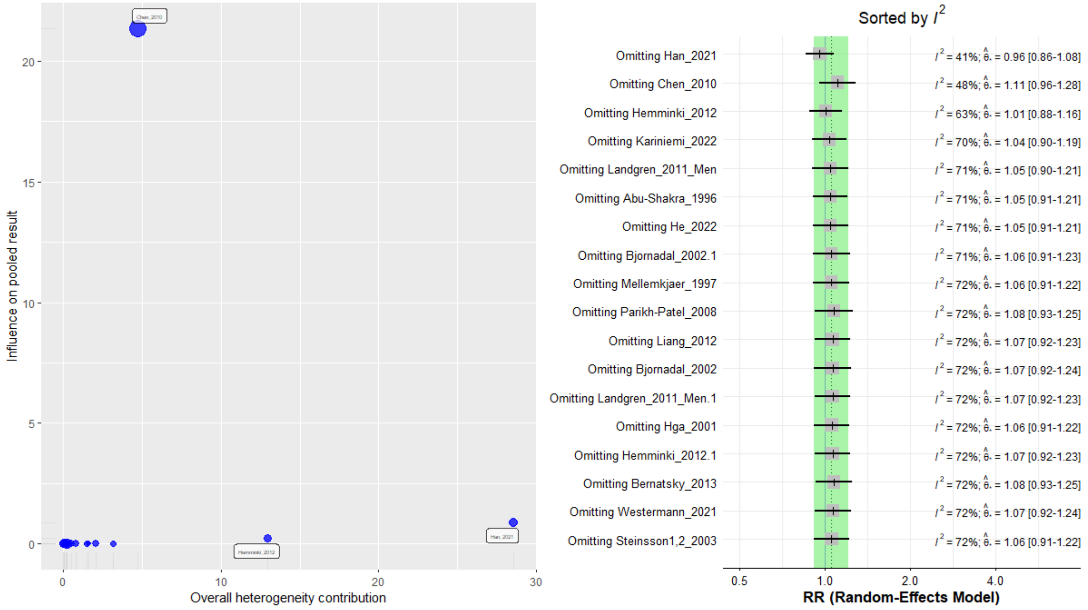

Lupus on Esophagus

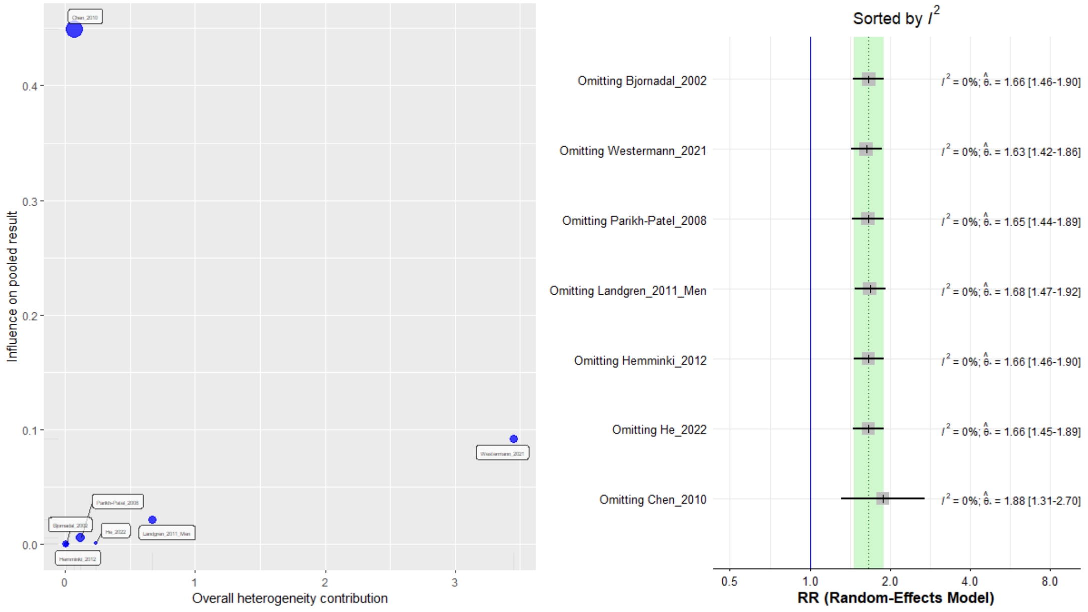

Lupus on Hepatobiliary

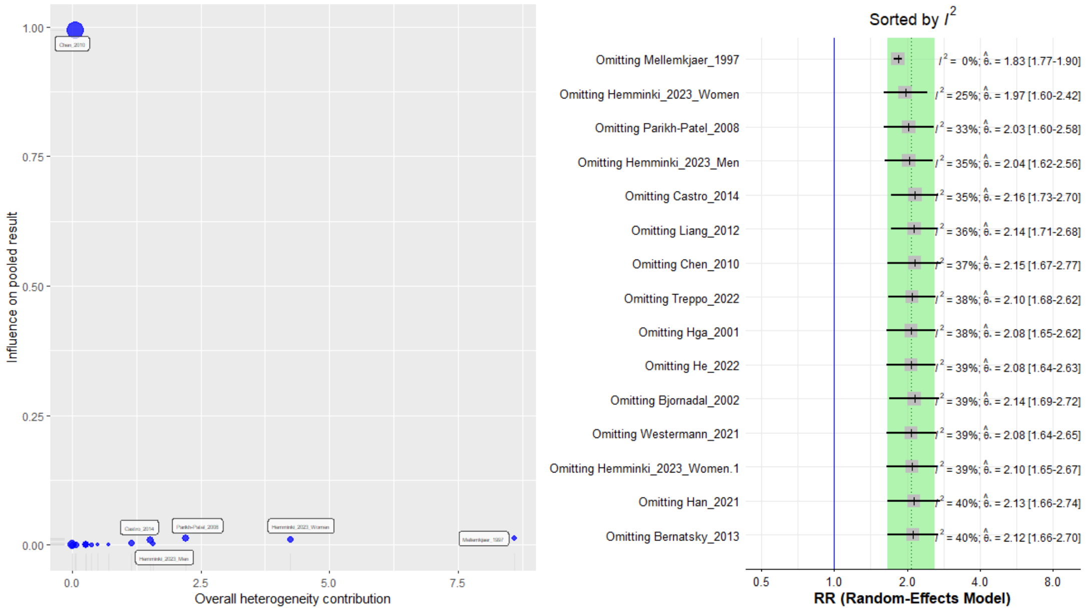

Lupus on Liver

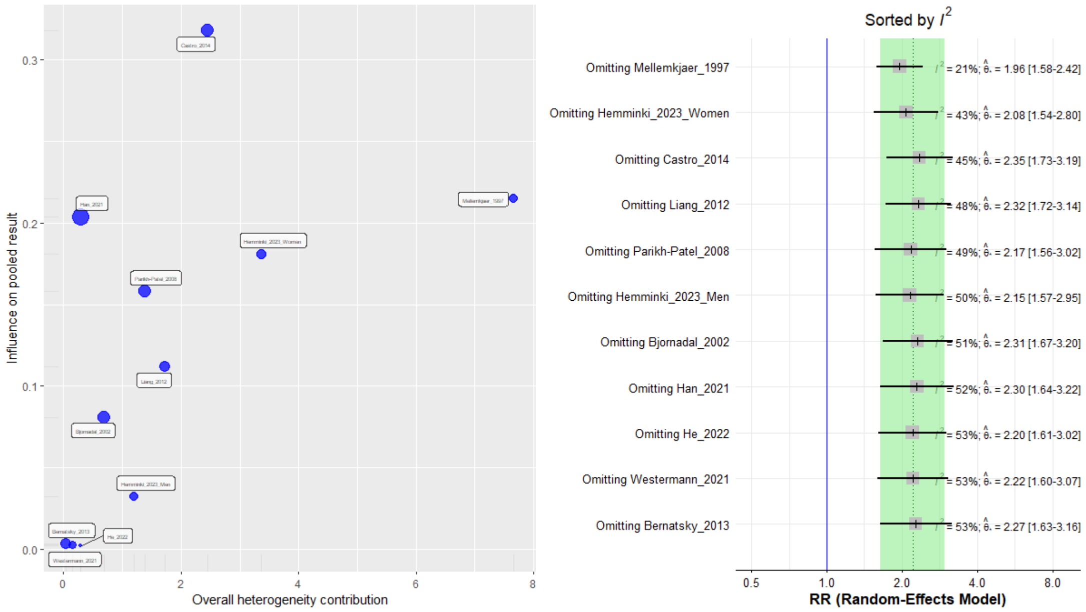

Lupus on Pancreas

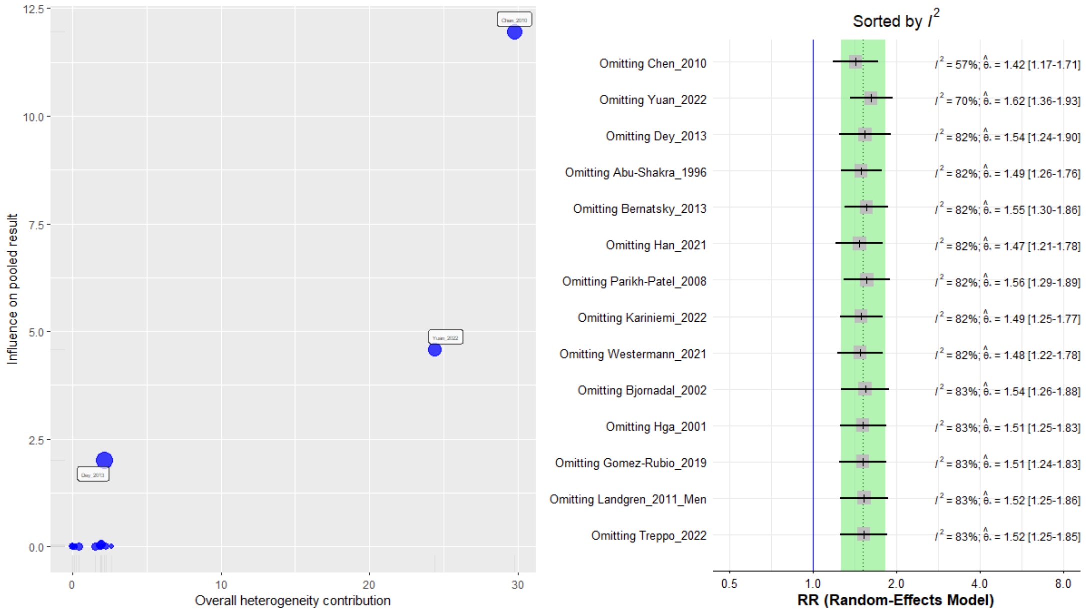

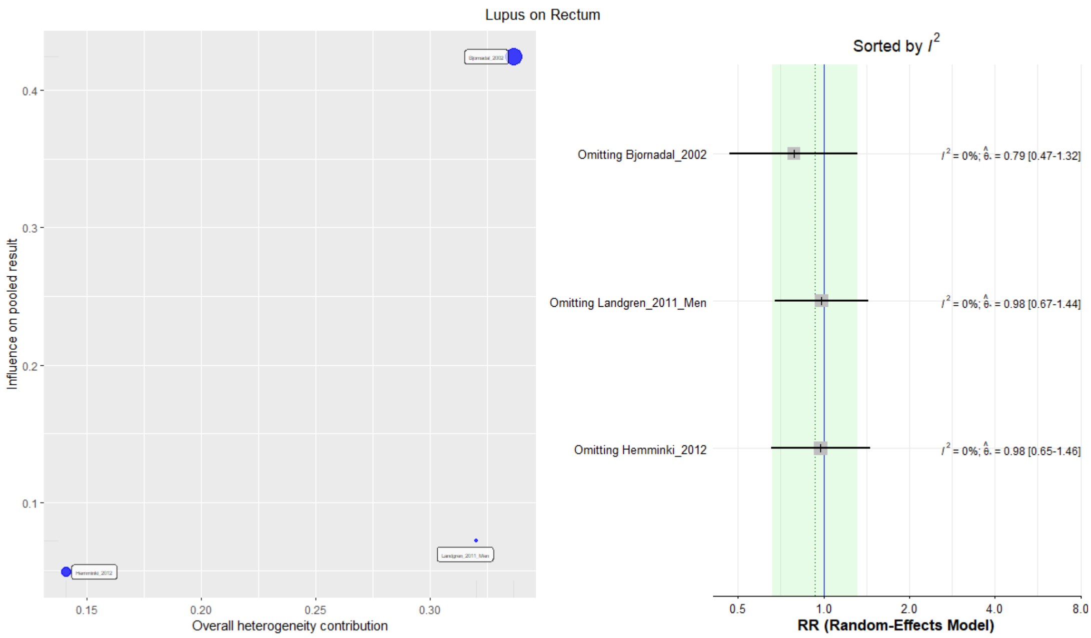

Figure 12: Influence analyses consisting of Baujat diagnostics and leave-one-out analyses (sorted by I^2) for the associations between systemic lupus erythematosus and different digestive system cancers.

Assessed associations are displayed above the respective plots.

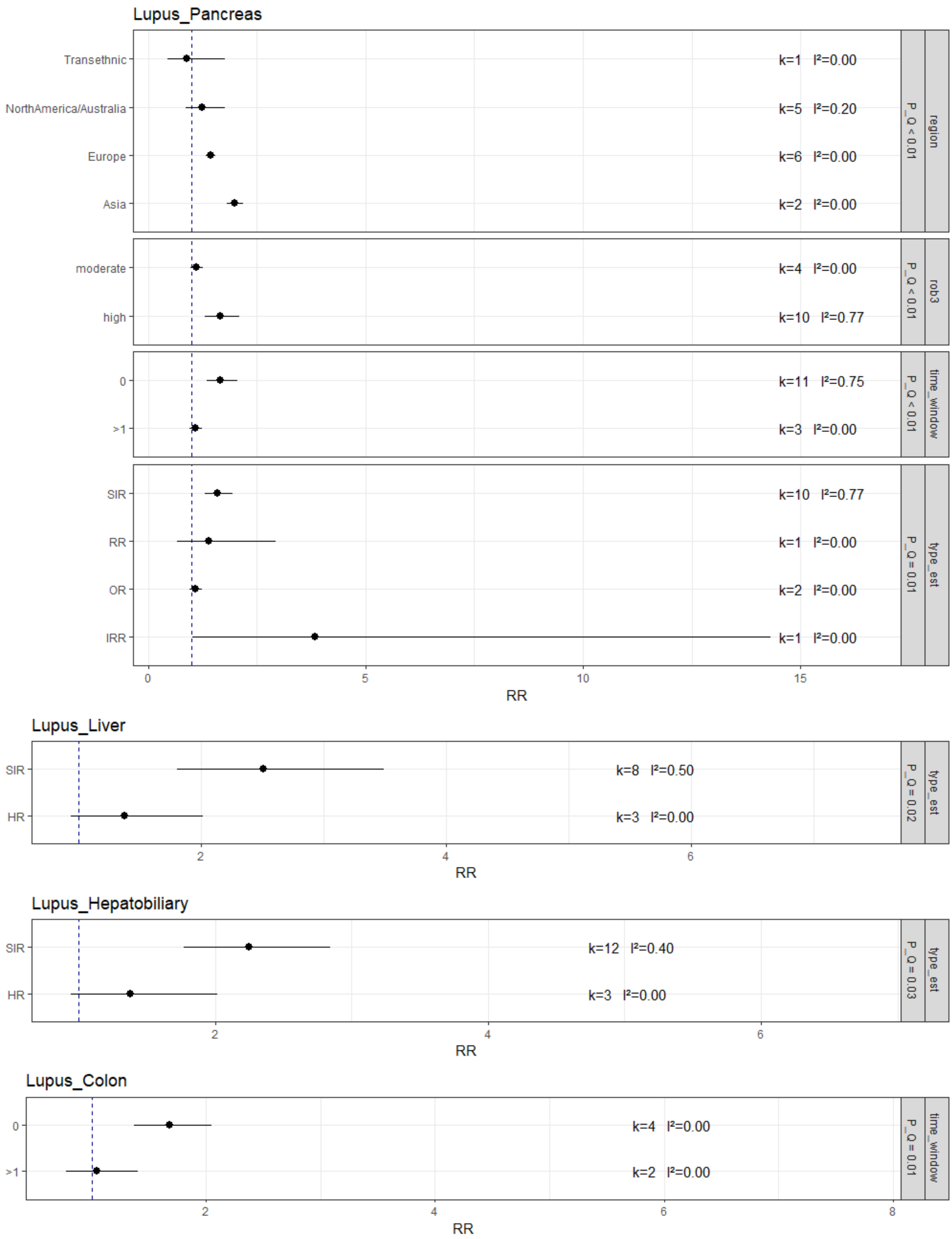

Figure 13: Subgroup analyses for the associations between systemic lupus erythematosus digestive system cancers

The time window indicates whether a study left a period of at least 1 year between the diagnosis of exposure and outcome.

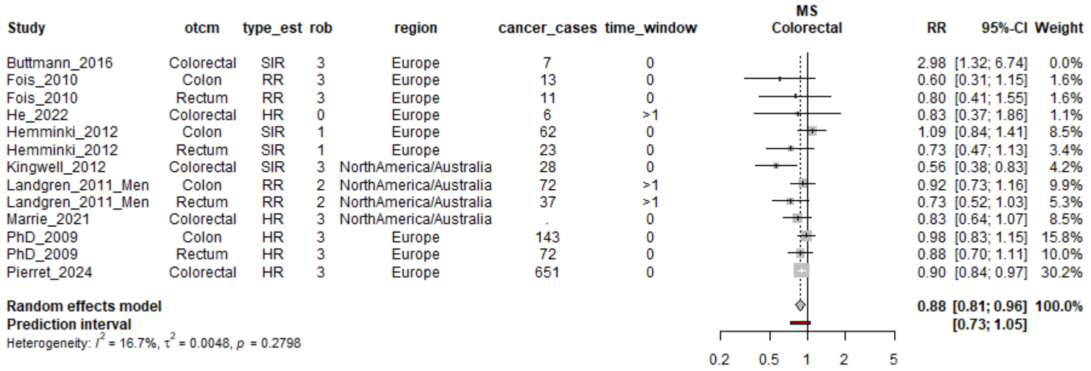

Figure 14: Outlier-adjusted meta-analyses for the associations between multiple sclerosis and different digestive system cancers.

Assessed associations are displayed above the respective forest plots. The time window indicates whether a study left a period of at least 1 year between the diagnosis of exposure and outcome. Cancer cases represent the number of incident cancer cases in the autoimmune disease group. Rob represents a weighted risk of bias score over the seven domains of ROBINS-E tool. Type_est shows the estimate-type used in the respective study (SIR=Standardized Incidence Ratio, RR=Relative Risk, HR=Hazard Ratio, OR=Odds Ratio, and IRR=Incidence Rate Ratio).

MS on Colon

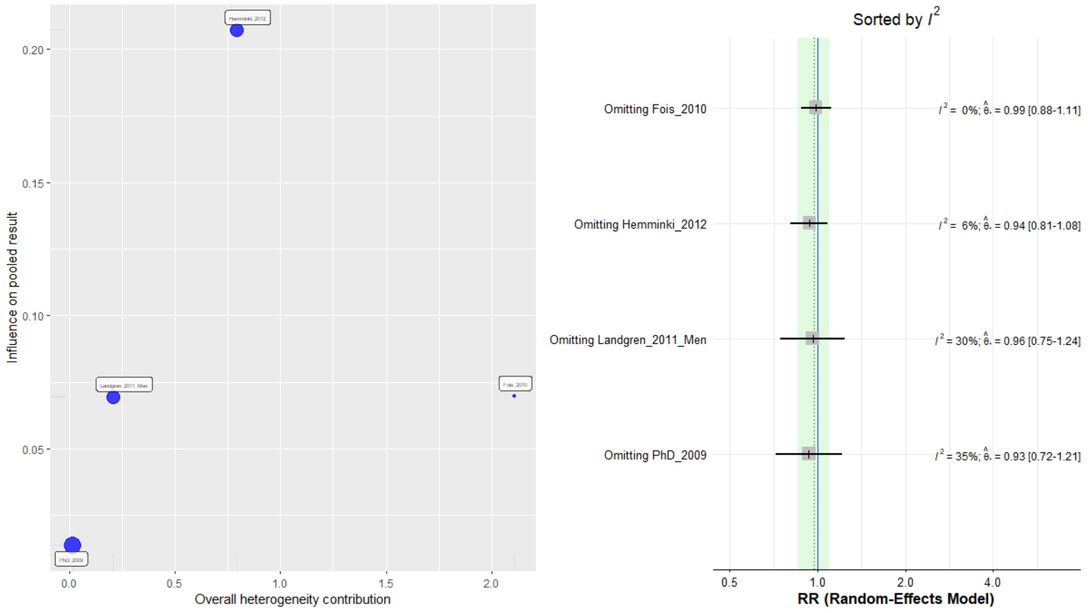

MS on Colorectal

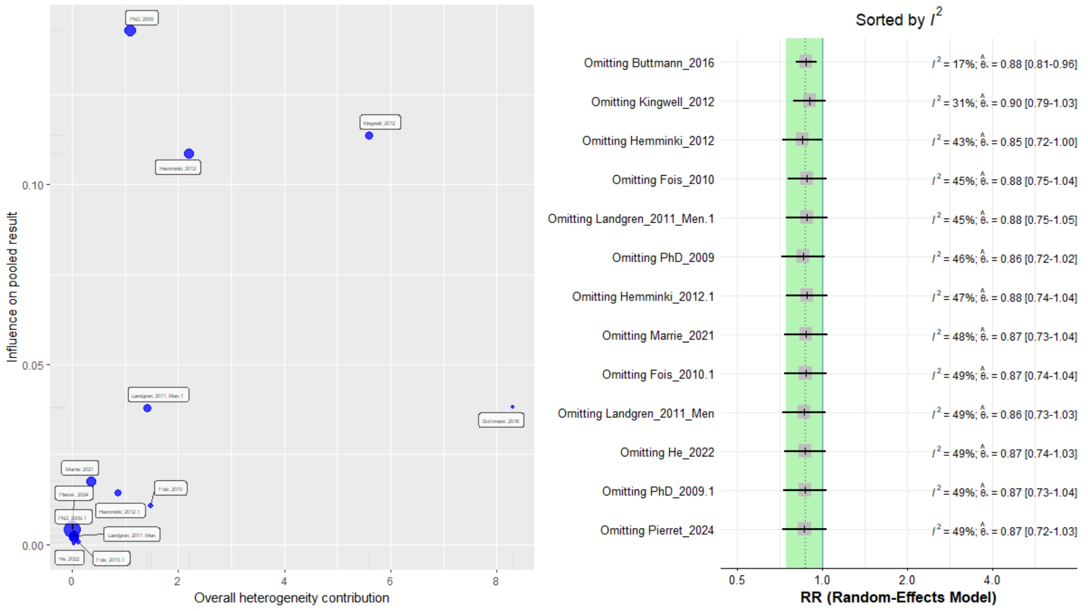

MS on Esophagus

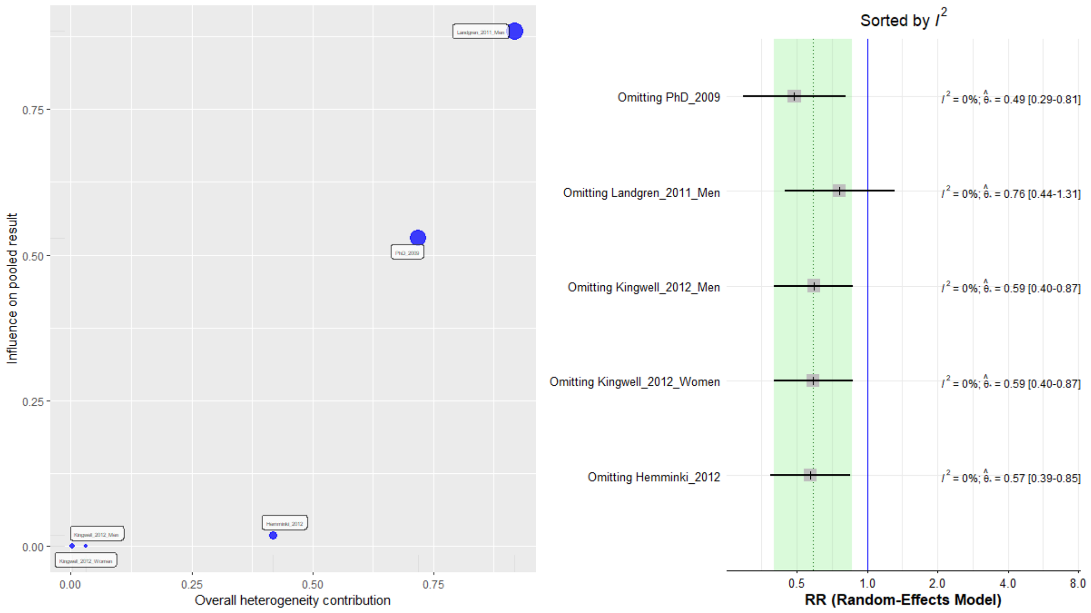

MS on Hepatobiliary

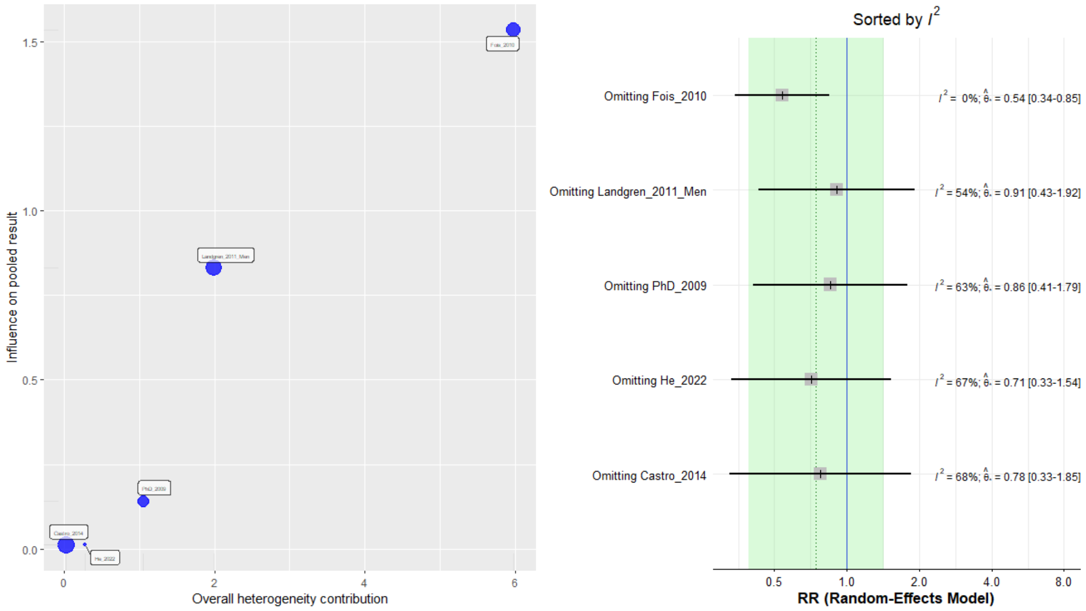

MS on Liver

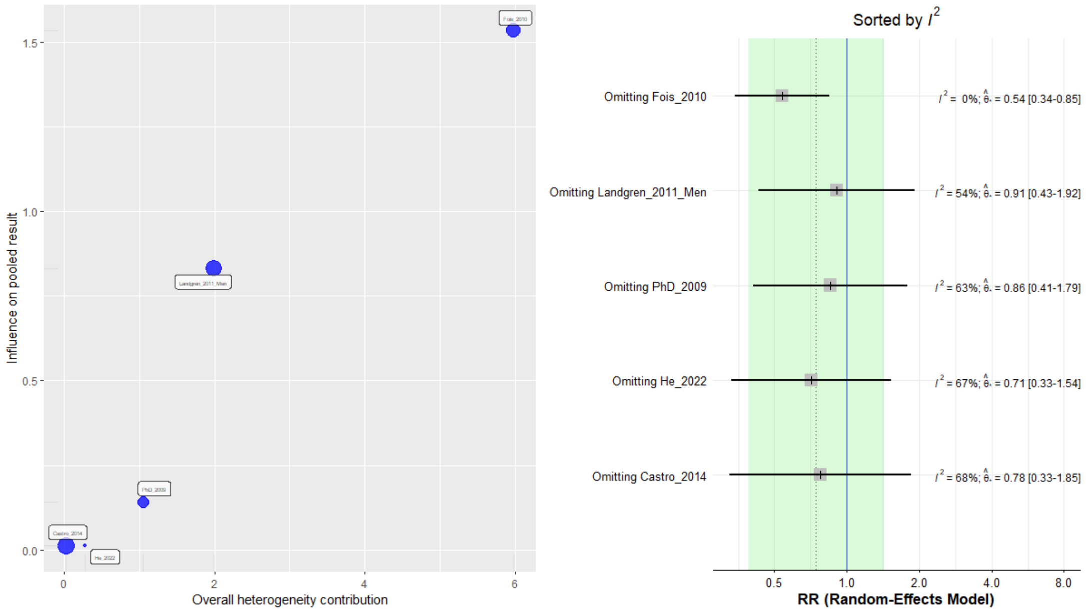

MS on Pancreas

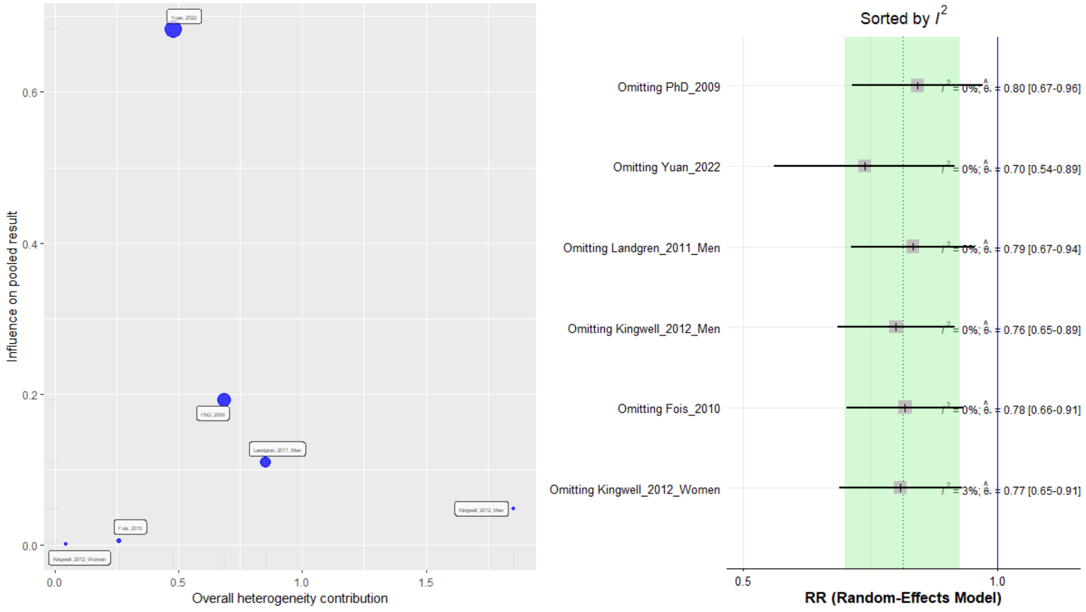

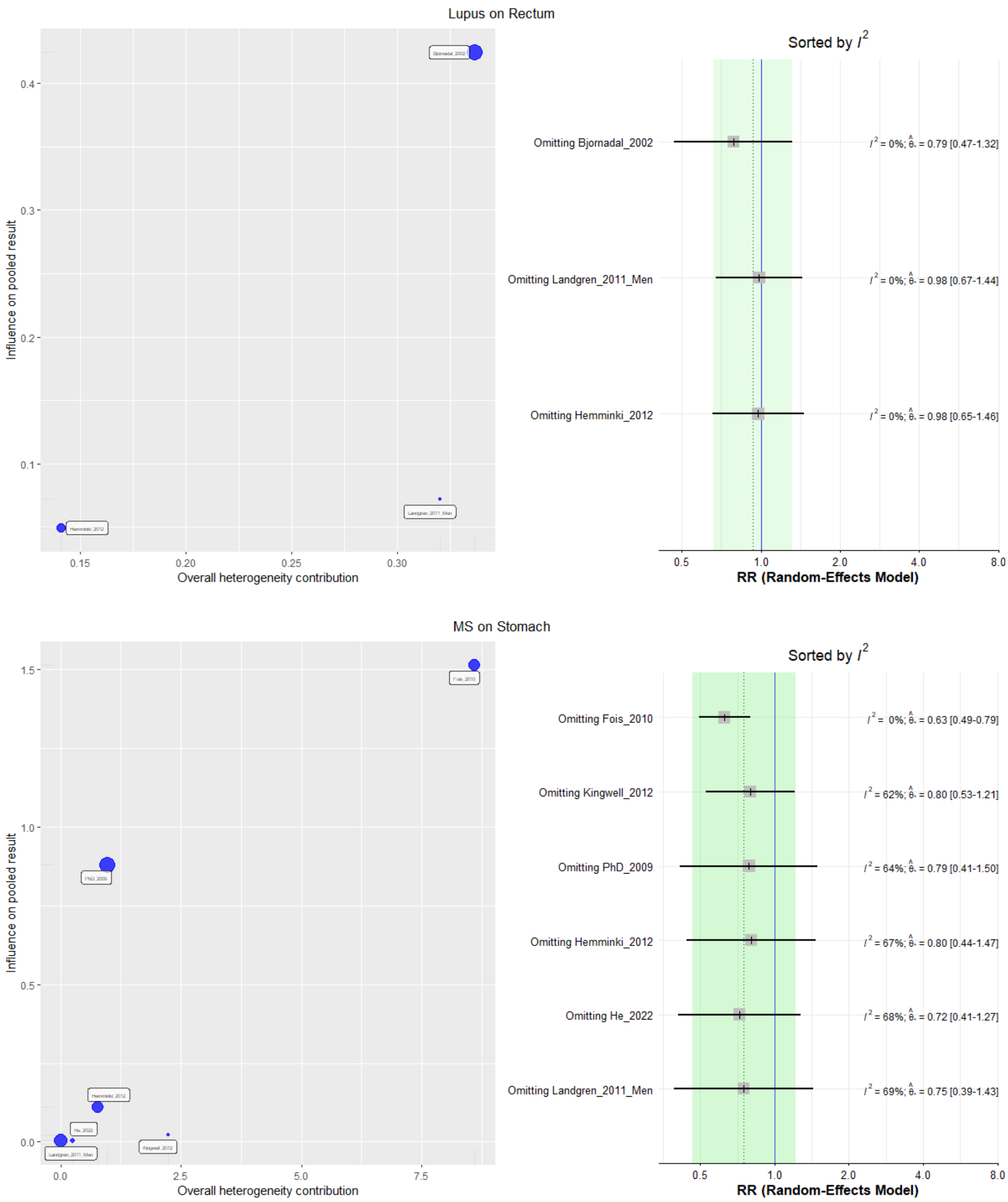

Figure 15: Influence analyses consisting of Baujat diagnostics and leave-one-out analyses (sorted by I^2) for the associations between multiple sclerosis and different digestive system cancers.

Assessed associations are displayed above the respective plots.

Study	otcm	type_est	rob	region	cancer_cases	time_window	RR	95%-CI	Weight
Carstensen_2016_Women	Colon	HR	5	Transethnic	483	0	1.06	[0.93; 1.21]	9.9%
Carstensen_2016_Men	Colon	HR	5	Transethnic	273	0	1.25	[1.11; 1.41]	10.6%
Carstensen_2016_Women	Rectum	HR	5	Transethnic	272	0	0.97	[0.81; 1.17]	8.0%
Carstensen_2016_Men	Rectum	HR	5	Transethnic	158	0	0.96	[0.82; 1.12]	9.1%
Carstensen_2016_Women	Colorectal	HR	5	Transethnic	876	0	1.09	[0.99; 1.21]	11.4%
Carstensen_2016_Men	Colorectal	HR	5	Transethnic	492	0	1.14	[1.04; 1.24]	11.9%
Harding_2015	Colorectal	SIR	3	NorthAmerica/Australia	237	0	1.21	[1.06; 1.38]	10.2%
He_2022	Colorectal	HR	1	Europe	28	>1	1.25	[0.85; 1.84]	3.2%
Hemminki_2012	Colon	SIR	2	Europe	4	0	0.96	[0.30; 3.03]	0.5%
Hemminki_2012	Rectum	SIR	2	Europe	5	0	2.06	[0.75; 5.62]	0.6%
Hsu_2015_Men	Colon	SIR	3	Asia	31	0	1.50	[1.04; 2.17]	3.5%
Hsu_2015_Women	Colon	SIR	3	Asia	39	0	1.95	[1.41; 2.70]	0.0%
Hsu_2015_Men	Rectum	SIR	3	Asia	27	0	1.64	[1.10; 2.43]	3.1%
Hsu_2015_Women	Rectum	SIR	3	Asia	15	0	1.07	[0.62; 1.84]	1.9%
Shu_2010	Colon	SIR	2	Europe	10	>1	1.18	[0.60; 2.33]	1.2%
Shu_2010	Rectum	SIR	2	Europe	5	>1	1.38	[0.51; 3.76]	0.6%
Swerdlow_2023	Colorectal	SIR	4	Europe	77	0	0.79	[0.63; 0.99]	0.0%
Valent_2015	Colon	HR	4	Europe	.	>1	1.81	[1.35; 2.43]	0.0%
Valent_2015	Rectum	HR	4	Europe	.	>1	1.89	[1.27; 2.82]	3.1%
Vicentini_2022	Colorectal	IRR	5	Europe	1	0	0.51	[0.07; 3.66]	0.2%
Zendehdel_2003	Colorectal	SIR	3	Europe	18	>1	1.10	[0.65; 1.85]	2.0%
Swerdlow_2005	Colorectal	SIR	4	Europe	14	0	0.71	[0.41; 1.24]	1.8%
Shin_2024	Colon	HR	2	Europe	100	0	1.36	[1.11; 1.68]	7.2%
**Random effects model**							**1.16**	**[1.07; 1.26]**	**100.0%**
**Prediction interval**								**[0.91; 1.48]**	

Heterogeneity: $I^2 = 41.1\%$, $\tau^2 = 0.0117$, $p = 0.0291$

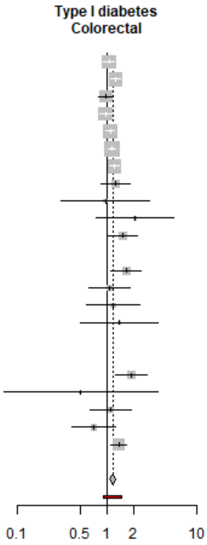

Study	otcm	type_est	rob	region	cancer_cases	time_window	RR	95%-CI	Weight
Carstensen_2016_Women	Liver	HR	5	Transethnic	154	0	1.55	[1.14; 2.10]	10.1%
Carstensen_2016_Men	Liver	HR	5	Transethnic	113	0	2.00	[1.67; 2.40]	12.5%
Harding_2015	Gallbladder	SIR	3	NorthAmerica/Australia	6	0	0.81	[0.36; 1.81]	3.7%
Harding_2015	Liver	SIR	3	NorthAmerica/Australia	53	0	2.76	[2.11; 3.62]	10.8%
He_2022	Liver	HR	1	Europe	10	>1	2.82	[1.43; 5.56]	4.7%
Hemminki_2023_Men	Liver	SIR	6	Europe	376	0	5.13	[4.63; 5.68]	0.0%
Hemminki_2023_Women	Liver	SIR	6	Europe	89	0	4.92	[3.98; 6.09]	0.0%
Hemminki_2023_Men	Gallbladder	SIR	6	Europe	28	0	2.22	[1.50; 3.28]	8.5%
Hemminki_2023_Women	Gallbladder	SIR	6	Europe	49	0	1.85	[1.38; 2.47]	10.4%
Hsu_2015_Men	Liver	SIR	3	Asia	92	0	1.63	[1.32; 2.01]	12.0%
Hsu_2015_Women	Liver	SIR	3	Asia	30	0	1.00	[0.69; 1.45]	8.8%
Shu_2010	Liver	SIR	2	Europe	3	>1	1.59	[0.40; 6.30]	1.5%
Swerdlow_2023	Liver	SIR	4	Europe	5	0	0.45	[0.17; 1.20]	0.0%
Swerdlow_2023	Gallbladder	SIR	4	Europe	2	0	0.52	[0.09; 2.88]	1.0%
Valent_2015	Liver	HR	4	Europe	.	>1	8.39	[6.57; 10.72]	0.0%
Valent_2015	Gallbladder	HR	4	Europe	.	>1	1.76	[0.73; 4.24]	3.2%
Vicentini_2022	Liver	IRR	5	Europe	1	0	2.00	[0.28; 14.29]	0.8%
Swerdlow_2005	Liver	SIR	4	Europe	1	0	0.70	[0.05; 9.80]	0.4%
Shin_2024	Liver	HR	2	Europe	90	0	1.39	[1.12; 1.73]	11.8%
**Random effects model**							**1.70**	**[1.42; 2.03]**	**100.0%**
**Prediction interval**								**[0.98; 2.96]**	

Heterogeneity: $I^2 = 61.7\%$, $\tau^2 = 0.0581$, $p = 0.0009$

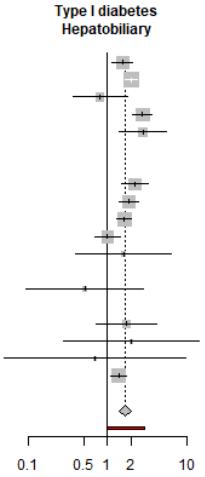

Study	otcm	type_est	rob	region	cancer_cases	time_window	RR	95%-CI	Weight
Carstensen_2016_Women	Liver	HR	5	Transethnic	154	0	1.55	[1.14; 2.10]	15.1%
Carstensen_2016_Men	Liver	HR	5	Transethnic	113	0	2.00	[1.67; 2.40]	21.1%
Harding_2015	Liver	SIR	3	NorthAmerica/Australia	53	0	2.76	[2.11; 3.62]	16.7%
He_2022	Liver	HR	1	Europe	10	>1	2.82	[1.43; 5.56]	5.5%
Hemminki_2023_Men	Liver	SIR	6	Europe	376	0	5.13	[4.63; 5.68]	0.0%
Hemminki_2023_Women	Liver	SIR	6	Europe	89	0	4.92	[3.98; 6.09]	0.0%
Hsu_2015_Men	Liver	SIR	3	Asia	92	0	1.63	[1.32; 2.01]	19.7%
Hsu_2015_Women	Liver	SIR	3	Asia	30	0	1.00	[0.69; 1.45]	0.0%
Shu_2010	Liver	SIR	2	Europe	3	>1	1.59	[0.40; 6.30]	1.6%
Swerdlow_2023	Liver	SIR	4	Europe	5	0	0.45	[0.17; 1.20]	0.0%
Valent_2015	Liver	HR	4	Europe	.	>1	8.39	[6.57; 10.72]	0.0%
Vicentini_2022	Liver	IRR	5	Europe	1	0	2.00	[0.28; 14.29]	0.8%
Swerdlow_2005	Liver	SIR	4	Europe	1	0	0.70	[0.05; 9.80]	0.4%
Shin_2024	Liver	HR	2	Europe	90	0	1.39	[1.12; 1.73]	19.2%
**Random effects model**							**1.84**	**[1.54; 2.20]**	**100.0%**
**Prediction interval**								**[1.17; 2.89]**	

Heterogeneity: $I^2 = 60.7\%$, $\tau^2 = 0.0305$, $p = 0.0091$

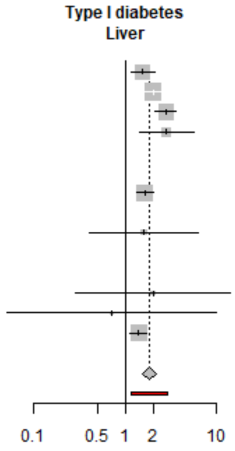

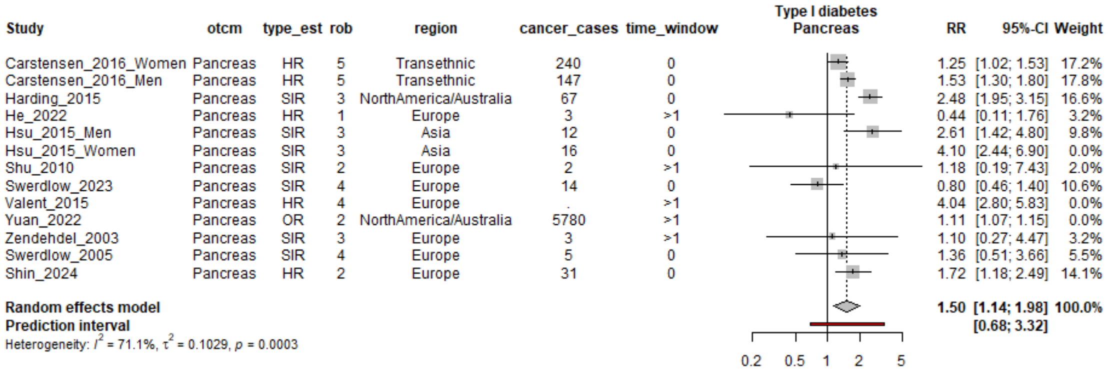

Figure 16: Outlier-adjusted meta-analyses for the associations between type 1 diabetes mellitus and different digestive system cancers.

Assessed associations are displayed above the respective forest plots. The time window indicates whether a study left a period of at least 1 year between the diagnosis of exposure and outcome. Cancer cases represent the number of incident cancer cases in the autoimmune disease group. Rob represents a weighted risk of bias score over the seven domains of ROBINS-E tool. Type_est shows the estimate-type used in the respective study (SIR=Standardized Incidence Ratio, RR=Relative Risk, HR=Hazard Ratio, OR=Odds Ratio, and IRR=Incidence Rate Ratio).

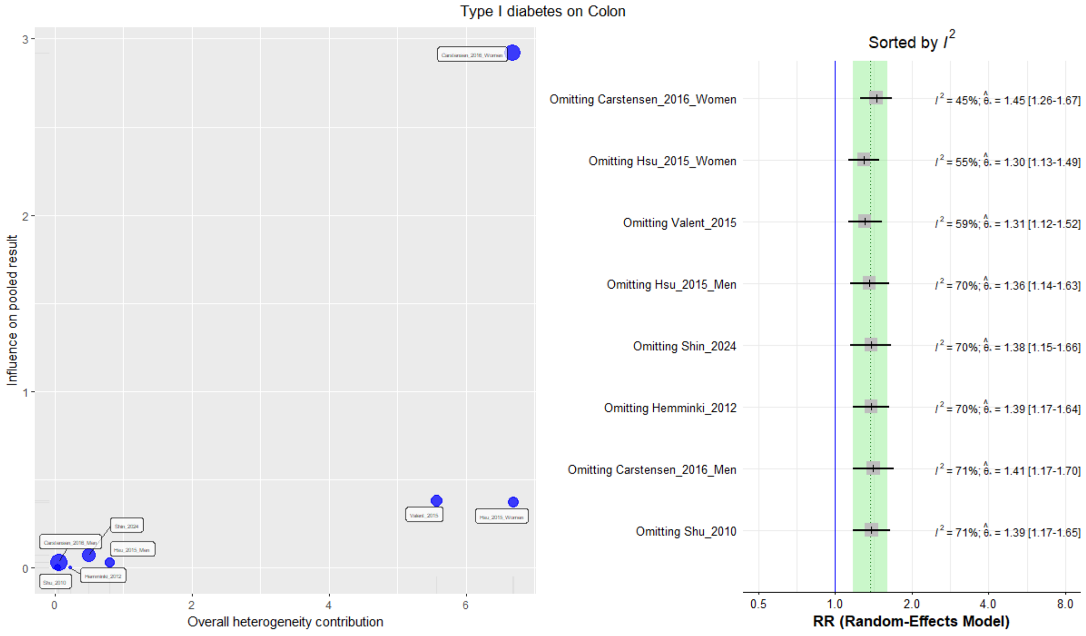

Type I diabetes on Colorectal

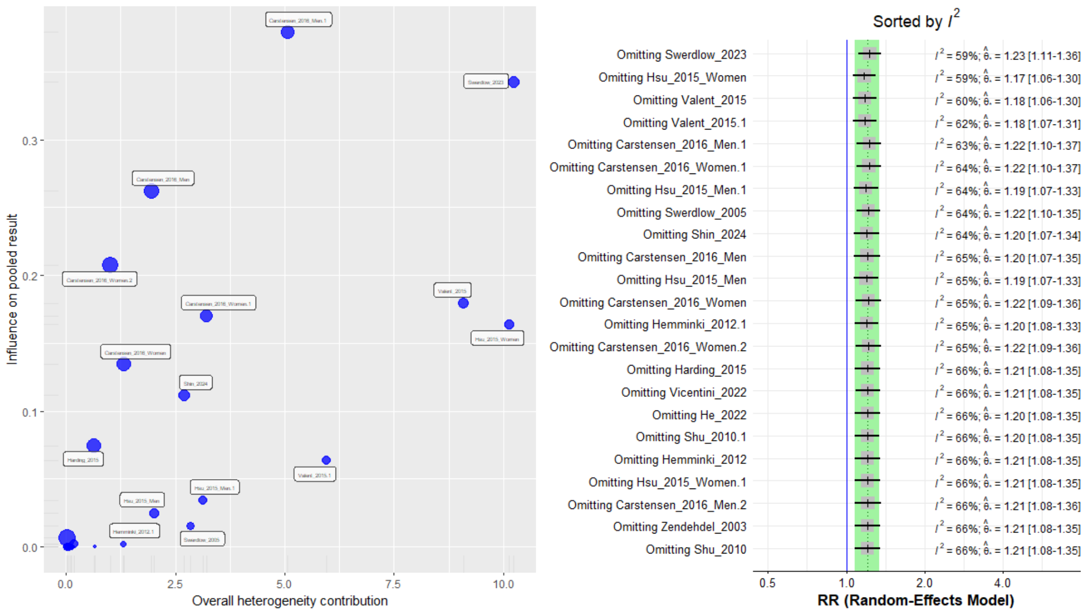

Type I diabetes on Esophagus

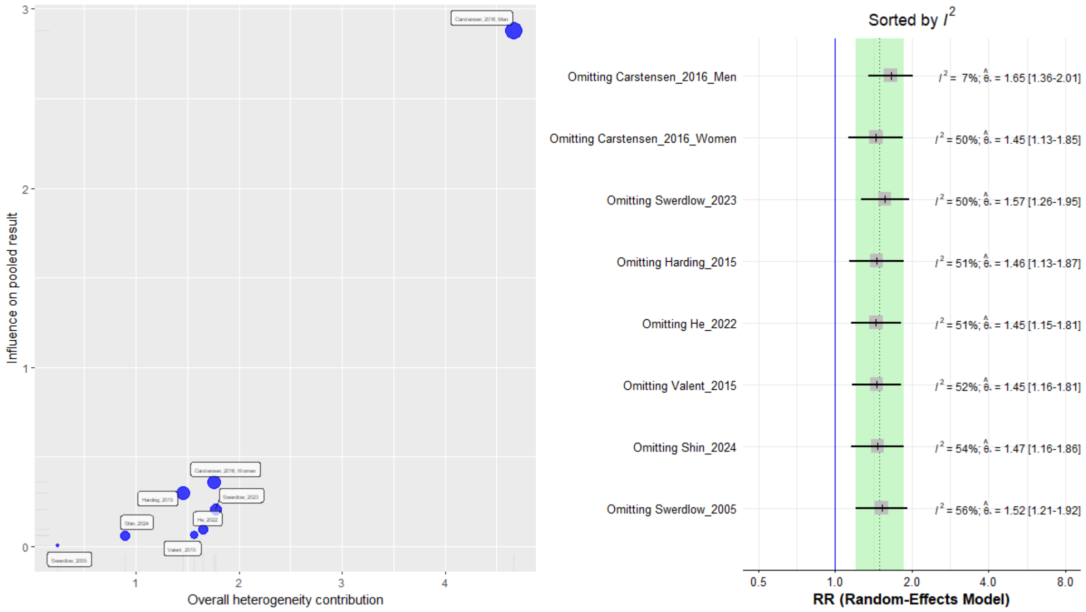

Type I diabetes on Gallbladder

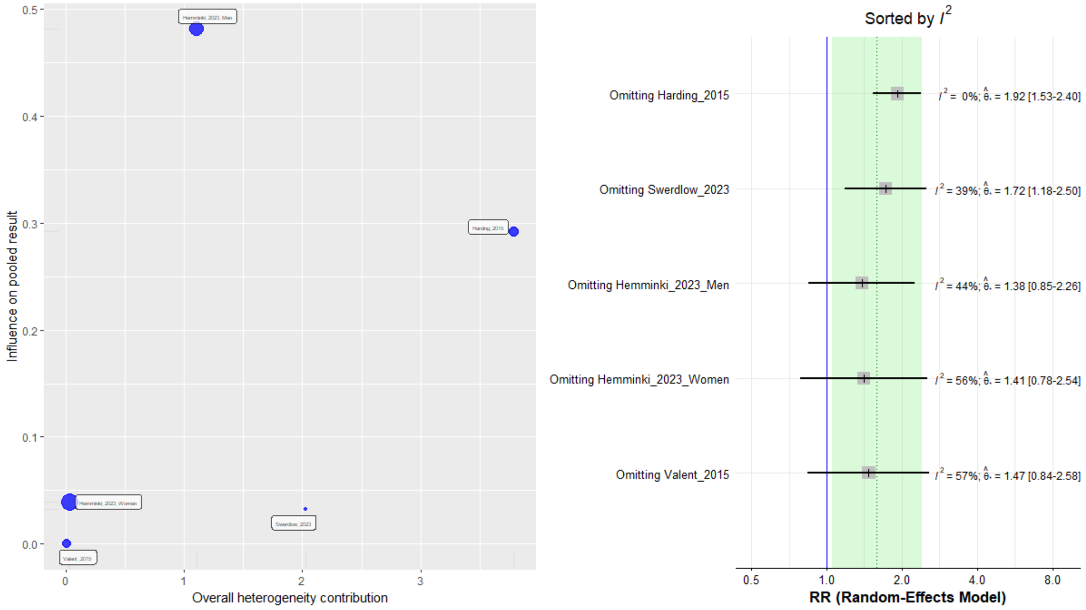

Type I diabetes on Hepatobiliary

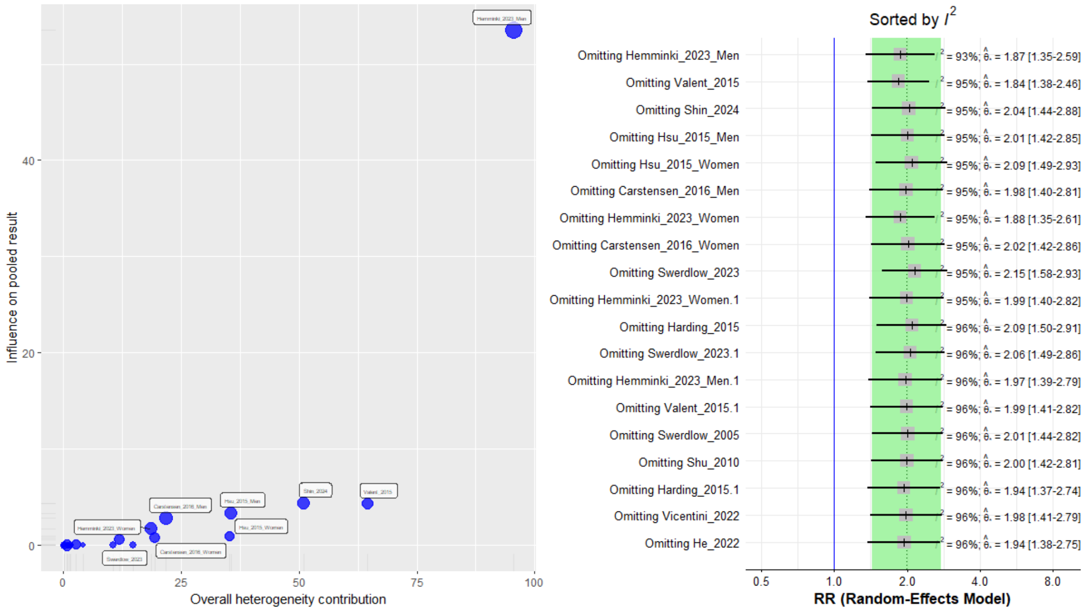

Type I diabetes on Liver

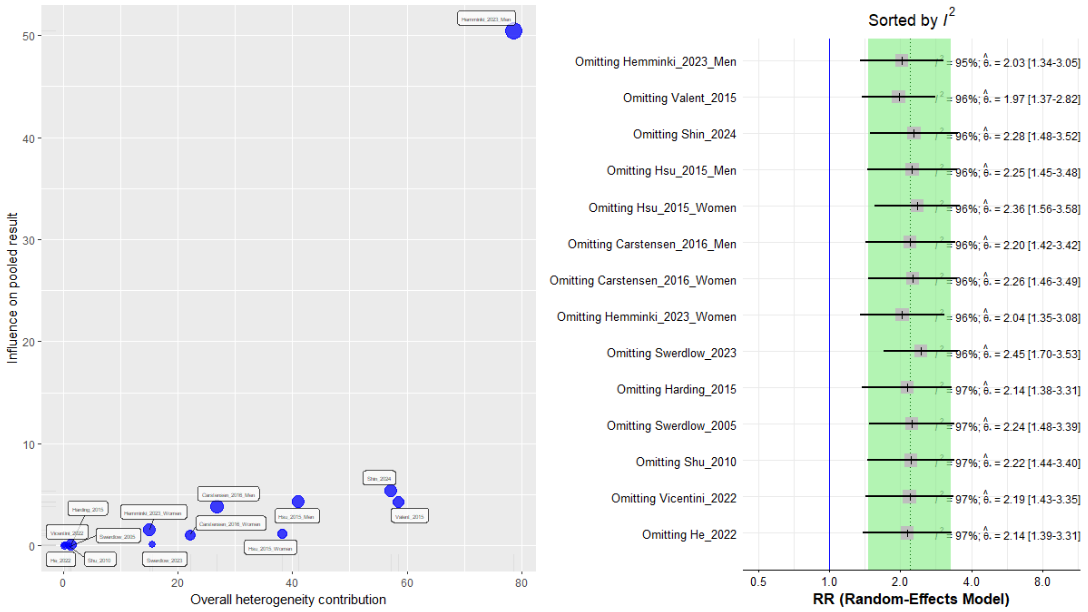

Type I diabetes on Pancreas

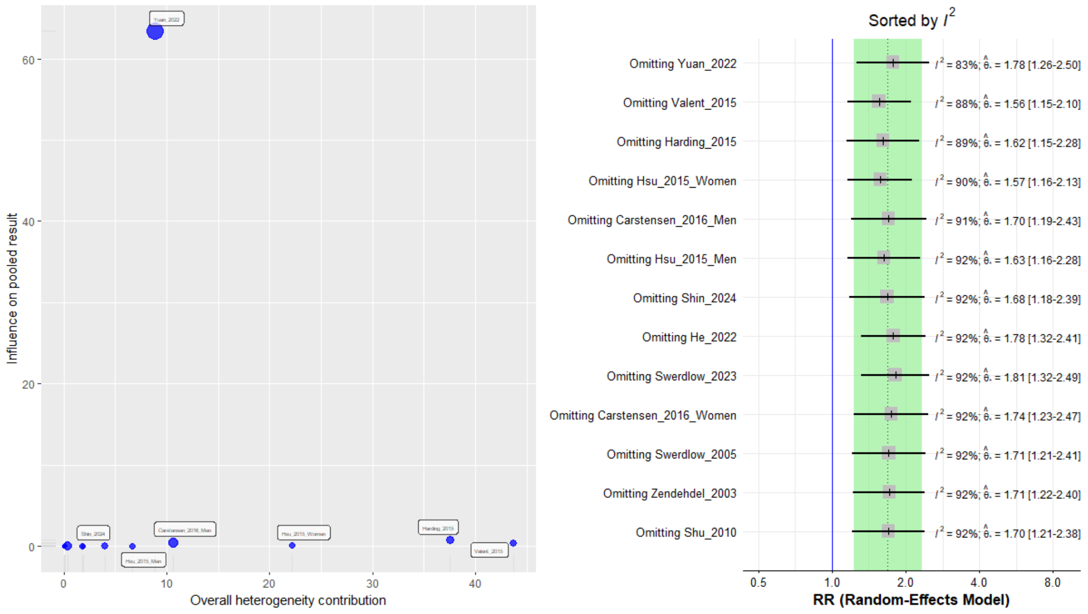

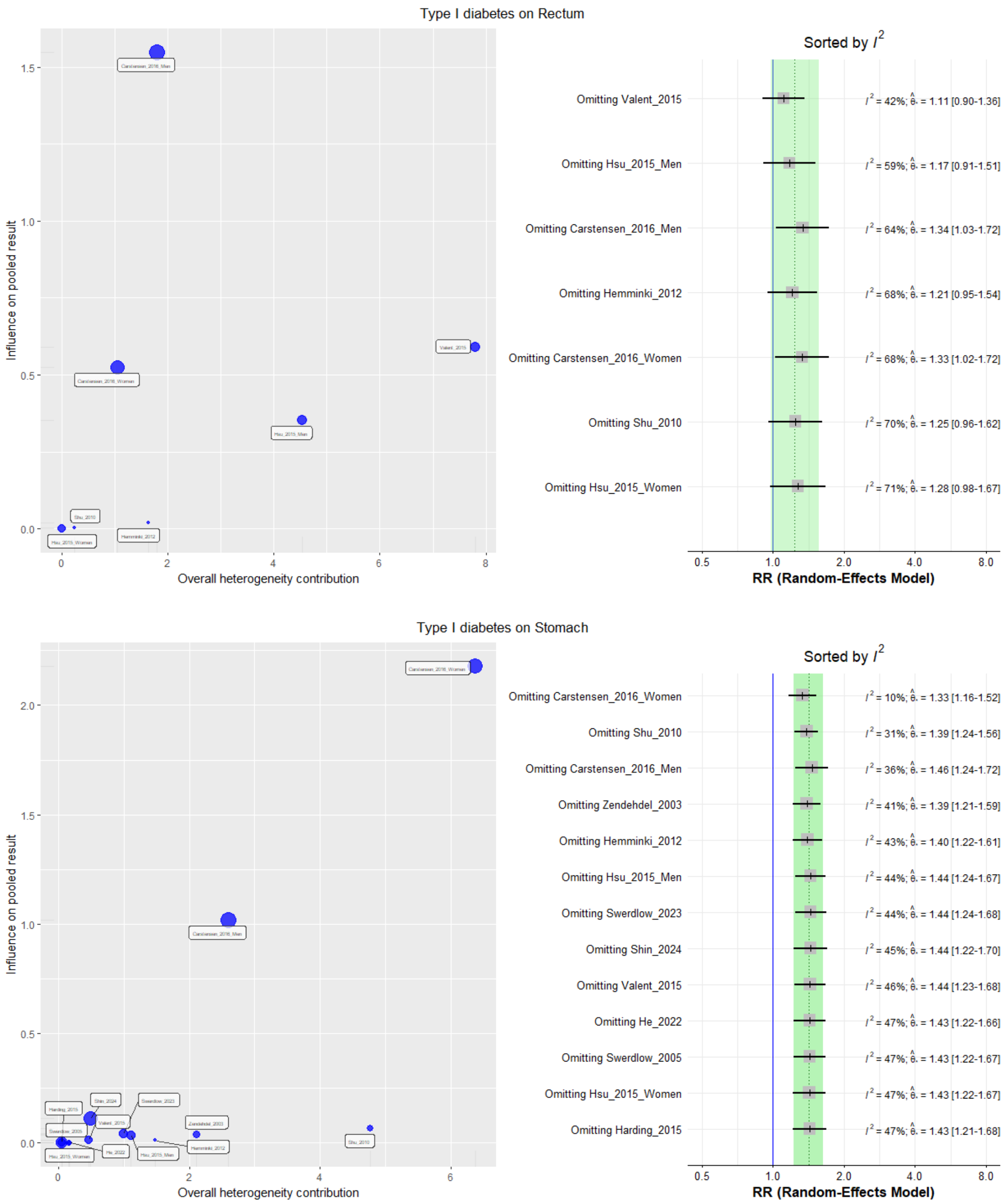

Figure 17: Influence analyses consisting of Baujat diagnostics and leave-one-out analyses (sorted by I^2) for the associations between type 1 diabetes mellitus and different digestive system cancers.

Assessed associations are displayed above the respective plots.

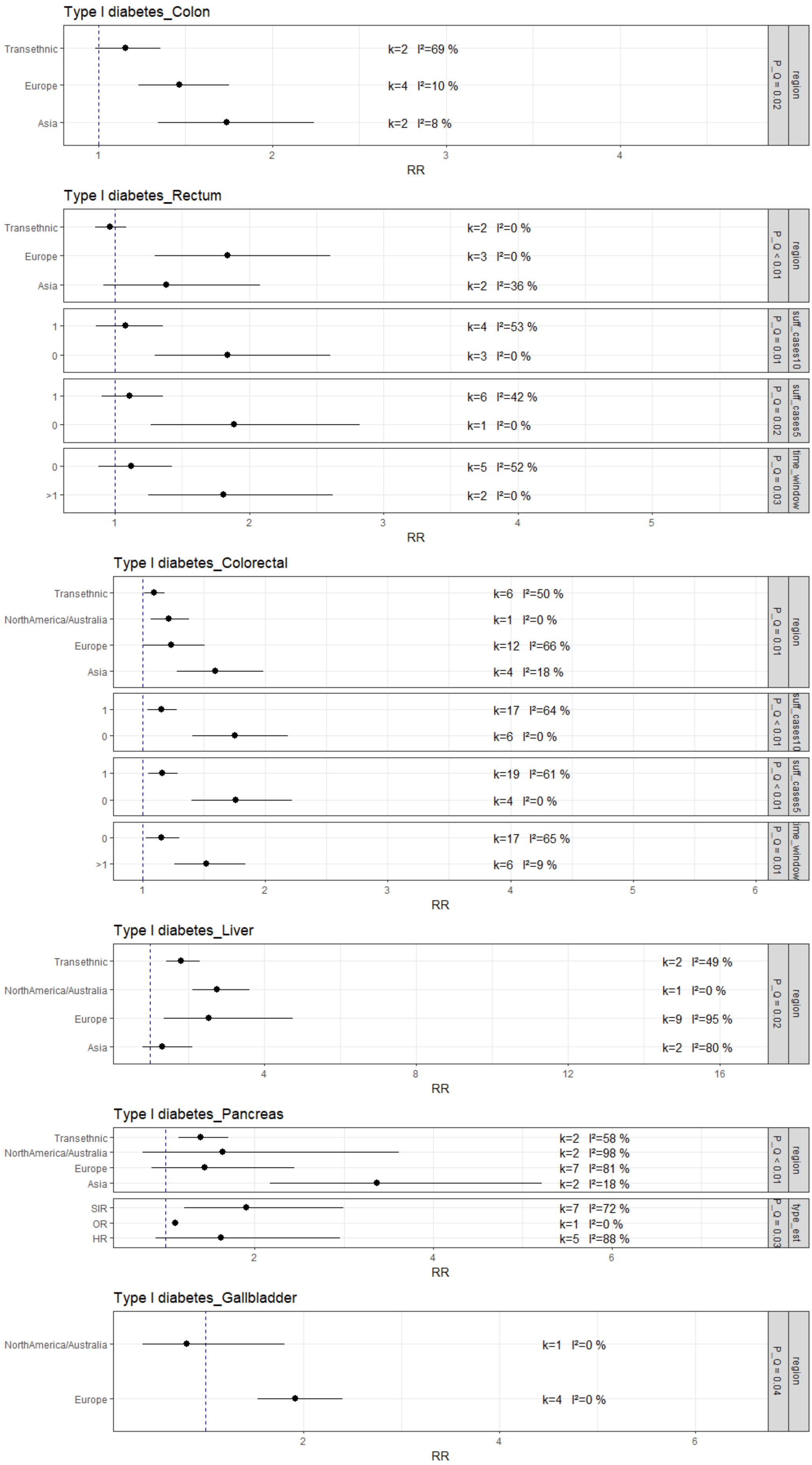

Figure 18: Subgroup analyses for the associations between type 1 diabetes mellitus and digestive system cancers

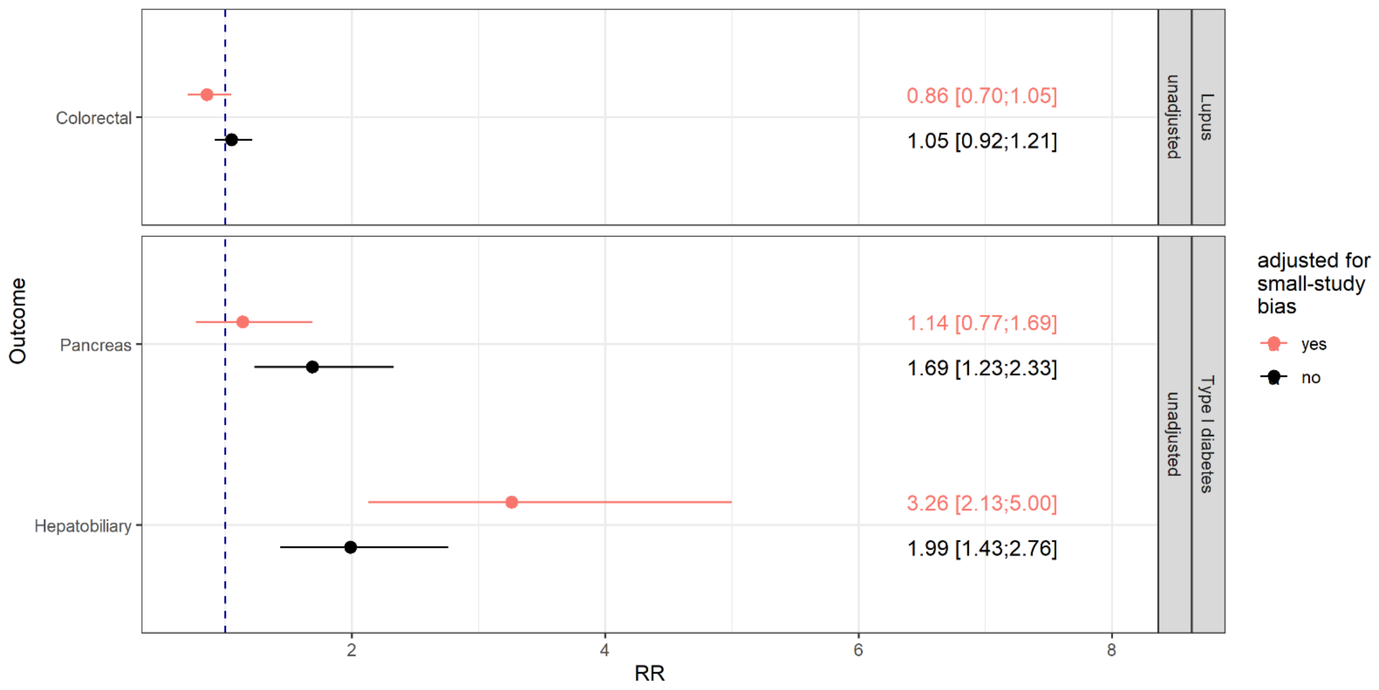

Figure 19: Associations adjusted for small-study bias

Small-study bias-corrected estimates were calculated using the trim-and-fill method and compared with the initial associations. Exposures and models used are listed on the right, outcomes on the left. Small-study bias could only be detected in models before outlier-adjustment.
